# Instruments for the assessment of suicide risk: A systematic review evaluating the certainty of the evidence

**DOI:** 10.1371/journal.pone.0180292

**Published:** 2017-07-19

**Authors:** Bo Runeson, Jenny Odeberg, Agneta Pettersson, Tobias Edbom, Ingalill Jildevik Adamsson, Margda Waern

**Affiliations:** 1 Department of Clinical Neuroscience, Karolinska Institutet, Stockholm, Sweden; 2 Centre for Psychiatry Research, Stockholm Health Care Services, Stockholm County Council, Stockholm, Sweden; 3 Swedish Agency for Health Technology Assessment and Assessment of Social Services, Stockholm, Sweden; 4 Östersund Hospital, Region Jämtland Härjedalen, Östersund, Sweden; 5 Department of Psychiatry and Neurochemistry, University of Göteborg, Göteborg, Sweden; Yokohama City University, JAPAN

## Abstract

**Background:**

Instruments have been developed to facilitate suicide risk assessment. We aimed to evaluate the evidence for these instruments including assessment of risk of bias and diagnostic accuracy for suicide and suicide attempt.

**Methods:**

PubMed (NLM), PsycInfo, Embase, Cinahl and the Cochrane Library databases were searched until December 2014. We assessed risk of bias with QUADAS-2. The average sensitivity and specificity of each instrument was estimated and the certainty of the evidence was assessed with GRADE. We considered instruments with a sensitivity > 80% and a specificity > 50% to have sufficient diagnostic accuracy.

**Results:**

Thirty-five relevant studies were identified but 14 were considered to have high risk of bias, leaving 21 studies evaluating altogether 15 risk assessment instruments. We could carry out meta-analyses for five instruments. For the outcome suicide attempt SAD PERSONS Scale had a sensitivity of 15% (95% CI 8–24) and specificity of 97% (96–98), and the Manchester Self-Harm Rule (MSHR) a sensitivity of 97% (97–97) and a specificity of 20% (20–21). ReACT, which is a modification of MSHR, had a similar low specificity, as did the Sodersjukhuset Self Harm Rule. For the outcome suicide, the Beck Hopelessness Scale had a sensitivity of 89% (78–95) and specificity of 42% (40–43).

**Conclusions:**

Most suicide risk assessment instruments were supported by too few studies to allow for evaluation of accuracy. Among those that could be evaluated, none fulfilled requirements for sufficient diagnostic accuracy.

## Introduction

At least 800 000 people around the world die by suicide every year (WHO 2014). Individuals with a history of suicidal behavior and coexisting mental disorder are at particular risk [1]. In western settings, approximately one third of suicide decedents had contact with mental health services at the time of death [2]. Inpatient care during the year prior to suicide was noted in about one fourth in a Swedish study [3]. In a recent British study, 0.7% of those who presented with self-harm died by suicide within an interval of 12 months [4].

Clinical assessment of persons who are at risk of fatal and non-fatal self-harm can in itself be the start of suicide preventive efforts. Guidelines have been developed to aid clinicians in this challenging endeavor [5, 6], and assessment instruments can supplement the clinical evaluation [7, 8]. Several recent reviews have examined the predictive validity of suicide assessment instruments, demonstrating poor performance in the prediction of subsequent suicide attempt and suicide [5, 9–11]. However, these reviews did not describe selection procedures in detail, and risk of bias was not considered. To the best of our knowledge, the GRADE procedure for rating the certainty of the evidence has yet to be applied to studies that evaluate the performance of suicide risk instruments.

In this systematic review of the literature, we aimed to estimate the diagnostic accuracy of suicide risk instruments with acceptable risk of bias. We assessed the certainty of the evidence for the estimates according to GRADE, and overall utility was determined. This review was carried out at the request of the Swedish Agency for Health Technology Assessment and Assessment of Social Services (SBU) and a Swedish version of the report can be found in S1 File. The relationship between the Swedish version and the current English version is described in S2 File and permission from the SBU is shown in S3 File.

## Methods

The systematic review was conducted in accordance with the PRISMA statement [12]; for PRISMA checklist see S4 File. The review used the following inclusion criteria: Studies should be prospective and set in psychiatric services or primary care. They should evaluate the sensitivity and specificity for suicidal acts with actual rates of suicide and suicide attempts at follow-up as reference standard. There was no upper limit for the follow-up time. The sample size should be 50 patients or more and studies should be published 1990 and later.

The databases PubMed (NLM), EMBASE (Elsevier), Cochrane Library (Wiley) and Cinahl (EBSCO) were searched until December 2014. Reference lists, books and websites were used to identify further studies. We provide the search strategies in S1 Table.

Two pairs of reviewers with clinical and research expertise (IJ and BR or TE and MW) screened titles and abstracts independently. We retrieved full text articles if either or both of the reviewers considered a study potentially eligible. Both reviewers read the full texts, and consensus was reached regarding eligibility. S2 Table provides the excluded articles and reasons for exclusion.

### Assessment procedures

The reviewers independently assessed each eligible study for risk of bias using a modified version of the checklist QUADAS- 2 [13]. Studies were scored as having either high (unacceptable) or acceptable risk of bias. We included only studies with acceptable risk of bias in the further analyses and meta-analyses. Data synthesis was carried out using MetaDiSc 1.4 (Meta-analysis of diagnostic and screening tests, version 1.4). A meta-analysis was performed if at least two studies with at least 200 individuals each were available for a specific instrument and a specific outcome (suicide or suicide attempt). Positive and negative predictive values (PPV and NPV) were calculated when prevalence data were available.

We assessed the certainty of evidence (high, moderate, low or very low) with GRADE [14]. In short, the certainty of the evidence was initially classified as high [14]. We thereafter analyzed to what extent the summary sensitivity and specificity measure might be affected by the five risk domains in GRADE. These are: overall risk of bias, degree of heterogeneity between studies (inconsistency), size of the confidence intervals for the summary measures (imprecision), risk for publication bias and risk that the results are not generalizable (indirectness). The final certainty of the evidence depended on whether there were severe deficiencies in any of the five risk domains. Thus, the resulting certainty of evidence could be high, moderate, low or very low. For the domain imprecision, we rated down one step if the 95% CI exceeded 10% units and two steps if the 95% CI exceeded 20% units of the summary sensitivity and specificity.

We applied a special rule for instruments evaluated in a single study. If the sample included 200–1000 participants, the certainty of the evidence was downgraded one step, as generalizability to the target population was deemed less likely. When fewer than 200 persons were included in a study, we rated the certainty of the evidence as very low.

### Assessment of utility

There is no consensus in the literature regarding acceptable values of sensitivity and specificity for the predictive performance of suicide assessment instruments. For the purpose of this paper, we compared sensitivity and specificity figures for a given instrument to predetermined benchmark values (sensitivity > 80%, specificity > 50%). These values were determined pragmatically to correspond to a minimum level of acceptable diagnostic accuracy. Lower sensitivity would imply that the instrument failed to identify more than one out of five with future suicidal behavior, which would be unacceptable given the seriousness of the outcome.

## Results

**Fig 1:**
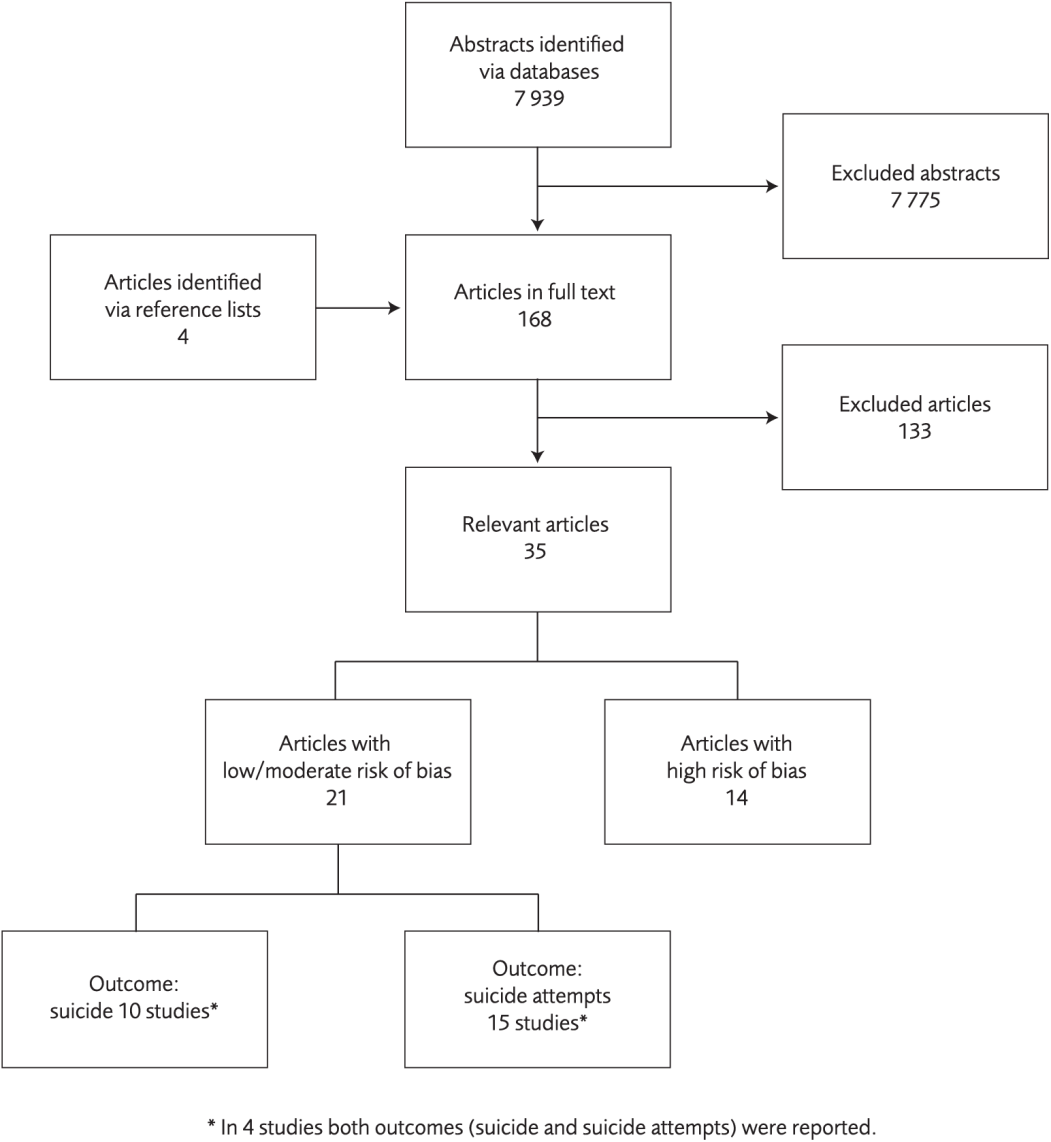
Flow of studies identified in literature search for systematic review on instrument for suicide risk assessment.

The database search yielded 7 939 abstracts; 172 articles were reviewed in full text, and 35 of these fulfilled inclusion criteria. We found high risk of bias in 14 studies [15–28], and these were excluded from further analyses. The remaining 21 studies [29–50] were deemed to have low or moderate risk of bias (Fig 1). Characteristics of the included studies are summarized in S3 Table.

### Diagnostic accuracy for suicide

**Fig 2:**
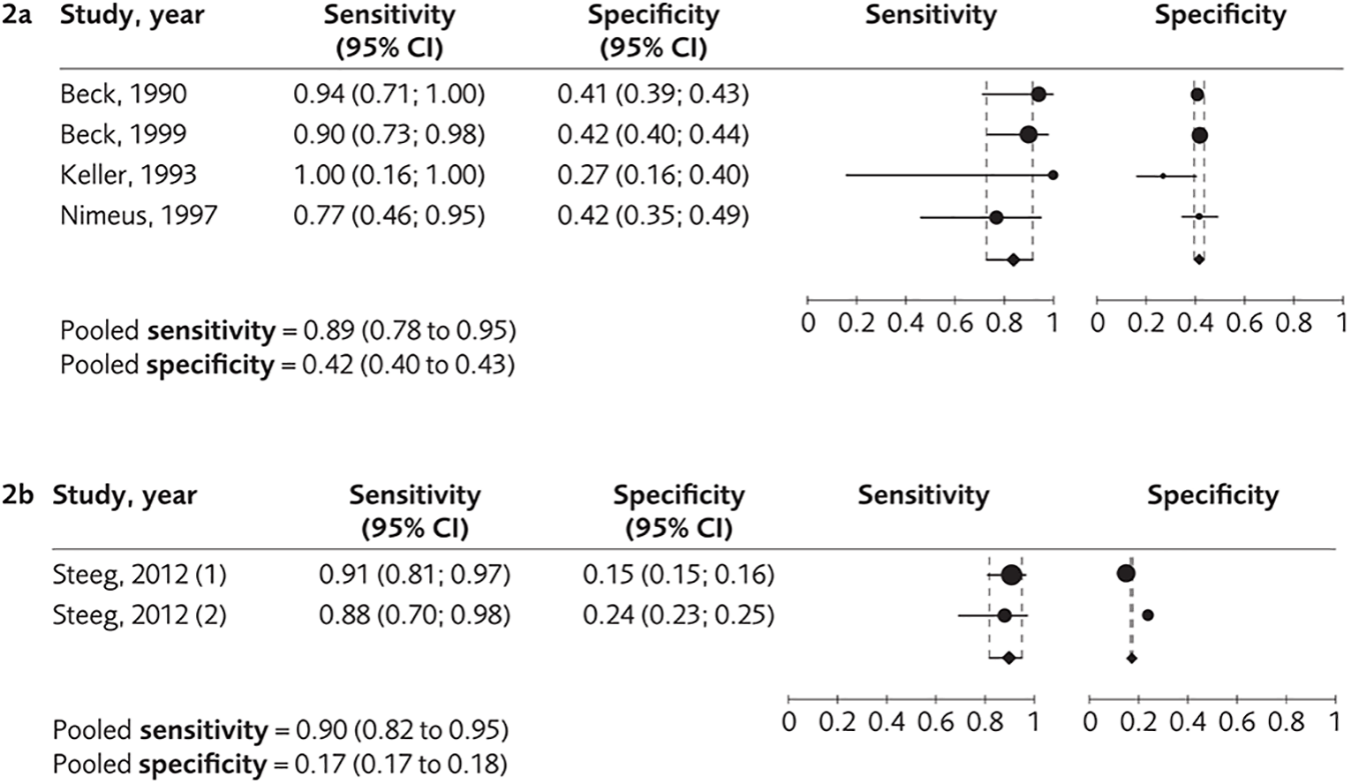
Meta-analyses for the diagnostic accuracy with regard to the suicide outcome. (a) The pooled sensitivity and specificity of the instrument BHS. (b) The pooled sensitivity of the instrument ReACT. In the study by Steeg et al, two sets of populations were included, here marked as (1) and (2).

Thirteen studies, mainly from Europe and North America, evaluated the diagnostic accuracy of eight instruments with regard to the suicide outcome. Follow-up intervals ranged from a few months to several years. All studies were conducted in psychiatric services with the exception of one primary care-based study which employed Patient Health Questionnaire (PHQ-9), which was set in primary care. Beck’s Hopelessness Scale, BHS, (when used for the population of first admission of psychosis) and Columbia Suicide Severity Rating Scale (C-SSRS) were each evaluated in a single small study and were not assessed further (Table 1). For five other instruments, Beck Depression Inventory (BDI), Scale for Suicide Ideation–Current (SSI-C), Scale for Suicide Ideation–Worst (SSI-W), Patient Health Questionnaire (PHQ-9) suicide item and Beck’s Suicide Intent Scale, SIS (tested in a population with self-harm), the studies showed that the sensitivity or lower limit of the confidence interval was below 80% (Table 2A). We present results of the meta-analyses for the diagnostic accuracy with regard to the suicide outcome in Fig 2A and 2B. For two instruments, BHS when used for a population with depression or anxiety disorder, and Recent Self-harm in the past year–Alone or homeless, Cutting used as a method, Treatment for a psychiatric disorder (ReACT) when used for a population with a self-harm act, the sensitivity was around 90% (Table 2A, Fig 2A and 2B). However, the specificity was low (42%) or very low (17%). Ratings of the certainty of the evidence for the diagnostic accuracy in accordance with GRADE are shown for each instrument in Table 2A.

**Table 1:** Instruments evaluated in studies with acceptable risk of bias.

Instrument	Reference	Population (No of participants)	Outcome
**Psychiatric specialist care**			
BDI	[31]	Depression and anxiety disorders, adults, (n = 1,958)	Suicide
BHS	[29–31, 37]	Depression and anxiety disorders, adults, (n = 5,932)	Suicide
BHS	[30]	Suicide attempt, all ages, (n = 61)	Suicide attempt
BHS	[42]	Depression, all ages, (n = 66)	Suicide attempt
BHS	[38]	Patients with first admission of psychosis, adults, (n = 414)	Suicide
C-SSRS	[48]	Depression, adolescents, (n = 124)	Suicide/suicide attempt
ERRS	[33]	Self-harm/suicide attempt, adults, (n = 1,317)	Suicide attempt
IAT	[45]	Self-harm/suicide attempt, age ≥17 years, (n = 127)	Suicide attempt
MINI ≥10	[46]	Acute psychiatric emergency departments, all ages, (n = 411)	Suicide attempt
SAD PERSONS Scale	[34, 43]	Self-harm/ psychiatric emergency departments, adults, (n = 2,972)	Suicide attempt
Modified SAD PERSONS Scale	[34]	Psychiatric emergency departments, adults, (n = 2,713)	Suicide attempt
SIS	[32]	Self-harm or deliberate self-harm, adolescents and adults, (N = 2,719)	Suicide
SIS	[49]	Serious suicide attempt, adolescents and adults, (n = 555)	Suicide
SPS	[50]	Children/adolescents, victims of abuse/ behavioural disorders, (n = 840)	Suicide attempt
SSI-C	[37]	Depression and anxiety disorders, adults, (n = 3,701)	Suicide
SSI-W	[37]	Depression and anxiety disorders, adults, (n = 3,701)	Suicide
SUAS	[40]	Suicide/suicide attempt, adults (n = 162)	Suicide/suicide attempt
**Emergency care**			
MSHR	[35, 36, 39, 44, 47]	Self-harm/suicide attempts/suicide, adults, (n = 29,772)	Suicide attempt
ReACT	[44]	Self-harm, adolescents and adults, (n = 16,680)	Suicide/suicide attempt
SoS-4	[35, 36]	Deliberate self-harm/suicide attempt, adults, (n = 1,849)	Suicide attempt
**Primary care**			
PHQ-9	[41]	Depression, adolescents and adults, (n = 84,418)	Suicide/suicide attempt

**Table 2:** Summary of findings, diagnostic accuracy for the outcomes a) suicide and b) suicide attempts.

Instrument (no. of studies) ^1^	Population	No of patients/ events	Sensitivity% (95% CI)	Specificity % (95% CI)	Certainty of evidence^2^	Comparison to bench mark
a. Suicide						
BHS (4 studies)	depression/ anxiety disorder	5 932 / 62	89 (78, 95)	42 (40, 43)	Moderate ^3^	Specificity too low
BDI	depression/ anxiety disorder	1 944/17	76 (50, 93)	62 (60,65)	Low ^4^	Sensitivity too low
SSI-C	depression/ anxiety disorder	3 701 / 30	53 (34, 72)	83 (82, 84)	Low ^4^	Sensitivity too low
SSI-W	depression/ anxiety disorder	3 701 /30	80 (61, 92)	78 (77, 79)	Moderate ^3^	Sensitivity probably too low
PHQ-9 suicide item	depression/ anxiety disorder	84 418 /46	80 (66, 91)	70 (70, 71)	Low ^5^	Sensitivity probably too low
ReACT	presenting after self-harm/suicide attempt	18 680 / 92	90 (82, 95)	17 (18, 18)	Moderate ^6^	Specificity too low
SIS ^**9**^	serious suicide attempt	555/22	59 (36, 79)	77 (74, 81)	Very Low^4^^,^^7^	Sensitivity too low
SIS ^**1**0^	presenting after self-harm/suicide attempt	2 719 / 54	76 (62, 87)	49 (47, 51)	Low ^3^^,^ ^7^	Sensitivity and specificity probably too low
b. Suicide attempt						
PHQ-9	patients with depression/ anxiety disorder	84 418 / 46	78 (74, 81)	70 (70, 71)	Moderate ^8^	Sensitivity probably too low
SAD PERSONS Scale (2 studies)	patients in psychiatric emergency care	2 972 /471	15 (8, 24)	97 (96, 98)	Strong	Sensitivity extremely low
Modified SAD PERSONS Scale	patients in psychiatric emergency care	2 713 /76	29 (19, 40)	89 (88, 90)	Low ^3^^,^^7^	Sensitivity too low
MINI Suicide module	patients in psychiatric emergency care	307 /64	61 (47, 73)	75 (69, 80)	Low ^3^^,^^7^	Sensitivity too low
MSHR (5 studies)	patients presenting after self-harm/suicide attempt	29 772/9523	97 (96, 97)	20 (20, 21)	Strong	Specificity too low
ERRS	presenting after self-harm/suicide attempt	1 317 /180	26 (20, 33)	84 (82, 86)	Moderate ^7^	Sensitivity too low
ReACT	presenting after self-harm/suicide attempt	18 680 /92	94 (93, 94)	24 (23, 25)	Moderate ^6^	Specificity too low
SPS	presenting after self-harm/suicide attempt	834 /29	48 /29, 67)	80 (77, 83)	Low ^3^^,^^7^	Sensitivity too low
SOS-4 (2 studies)	presenting after self-harm/suicide attempt	1 849 /389	90 (86, 93)	17 (15, 19)	Strong	Specificity too low

^1^ Instruments tested in one study unless otherwise specified

^2^ Evidence refers to the evaluation of strength of evidence in accordance with GRADE

^3^–1 precision, wide CI

^4^–2 precision (very wide CI)

^5^–1 precision (wide CI) and -1 bias (unclear reporting results)

^6^–1 heterogeneity

^7^–1 indirectness (only 1 study)

8–1 bias (unclear reporting results)

^9^ cut-off 19

^10^ cut-off 10

### Diagnostic accuracy for suicide attempt

**Fig 3:**
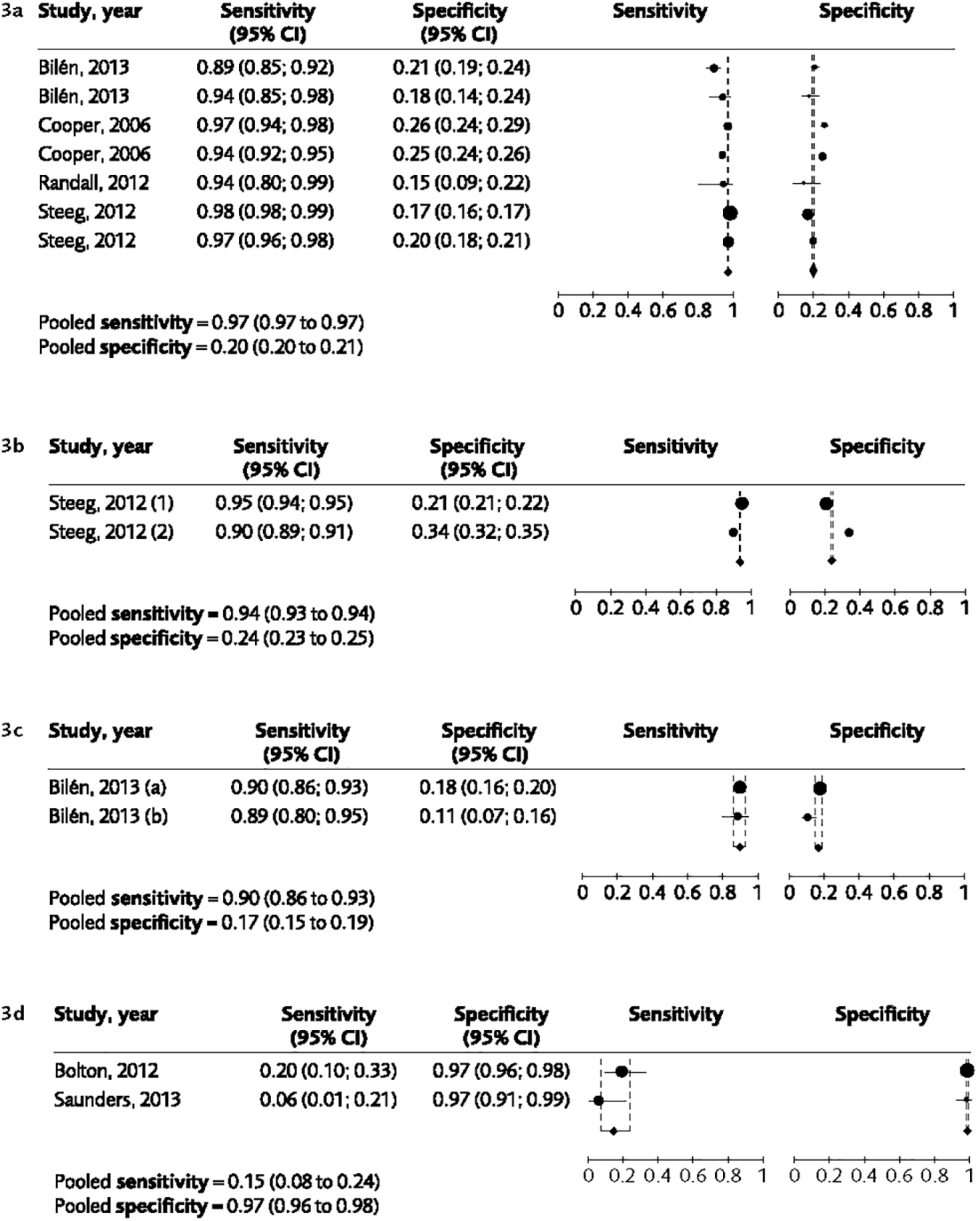
Meta-analyses for the diagnostic accuracy with regard to the outcome suicide attempts. (a) The pooled sensitivity and specificity of the instrument MSHR. (b) The pooled sensitivity and specificity of the instrument ReACT. In the study by Steeg et al, two sets of populations were included, here marked as (1) and (2). (c) The pooled sensitivity and specificity of the instrument SoS-4. (d) The pooled sensitivity and specificity of the instrument SAD PERSONS Scale.

Sixteen studies from Europe and North America evaluated the diagnostic accuracy for suicide attempts for 13 instruments with varying follow-up intervals. BHS, C-SSRS, Implicit Association Test (IAT) and Suicide Assessment Scale (SUAS) were only evaluated in one small study each and were not assessed further (Table 1). Three instruments, Manchester Self-Harm Rule (MSHR), ReACT and Sodersjukhuset Self-Harm Rule (SOS-4) had high sensitivity but specificities around 20% (Table 2B and Fig 3A–3C). SAD PERSONS Scale and modified SAD PERSONS Scale had very low sensitivity, around 20%, but high specificity (Table 2B and Fig 3D). The MINI–International Neuropsychiatric Interview suicide module, the Edinburg Risk of Repetition Scale (ERRS) and the Suicide Probability Scale (SPS) all showed low values for sensitivity (range for point estimate 26 to 61) but specificity of 75% or above. PHQ-9 evaluated in primary care had a sensitivity of 78% and specificity 70%. The certainty of the evidence according to GRADE for the diagnostic accuracy for the suicide attempt outcome is shown in Table 2B. The certainty of evidence was strong for the short scales MSHR and SOS-4.

### Assessment of utility

None of the instruments reached the predetermined benchmarks (80% sensitivity and 50% specificity) for the suicide outcome (Table 2A); the same was the case for the suicide attempt outcome (Table 2B). With few exceptions, low figures were observed for the positive predictive value (PPV) for the suicide outcome (1–13%). We observed higher values (7–40%, with a few outliers) for the suicide attempt outcome (S3 Table).

## Discussion

We examined the evidence for the diagnostic accuracy of suicide risk assessment tools in a systematic review. Out of the 15 instruments that qualified for assessment, none showed sufficient diagnostic accuracy, despite our relatively lenient limits for sensitivity and specificity. The GRADE procedure helped to distinguish between instruments that have been examined sufficiently and those that require further testing.

Two frequently used instruments, the Scale for Suicide Ideation and the Suicidal Intent Scale had too low sensitivity, but the certainty of evidence was low to moderate, which motivates further testing. We found strong evidence that the SAD PERSONS Scale has low diagnostic accuracy, and should thus not be used in its present form. Instruments for use in emergency units for triage to psychiatric care (the Manchester Self Harm Rule and Sodersjukhuset Self Harm Rule) had high sensitivity but very low specificity. These short scales were well studied in large samples and the certainty of evidence according to GRADE was strong, indicating that further studies will probably not change the results.

The low positive predictive value (PPV) of suicide risk assessment scales has previously been judged to disqualify them from use in prediction of subsequent suicidal behavior [5]. The present systematic review confirmed the particularly low PPV for predicting suicide and the only somewhat better PPV for predicting suicide attempt. Some researchers suggest that the use of suicide assessment instruments with high negative predictive value (NPV) may constitute a worthwhile clinical approach [10]. By enabling the identification of persons *not* at risk, resources could be more appropriately allocated to those at higher risk of fatal outcome. However, high NPV might reflect very sparse information about the subjects. An example could be the MSHR, where high specificity is based on the fact that a patient has none of the few risk factors captured by the instrument.

There were too few studies to assess the diagnostic accuracy of the Suicide Assessment Scale (SUAS) [40] and the Columbia—Suicide Severity Rating Scale (C-SSRS) [48]. Nor were there enough studies that investigated recent alternative assessment approaches such as the Implicit Association Test (IAT) [45].

Our analysis included only one study conducted in primary care. The certainty of the evidence for the predictive validity of the PHQ suicide item in this setting was rated as low, indicating a need for more research. Another commonly used scale, the Montgomery Asberg Depression Rating Scale (MADRS) [51], is also in need of testing. We found no studies that evaluated the predictive validity of the MADRS suicide item.

The instruments included in this systematic review were of varying length and character; some included just a few factors and others more than twenty variables. One instrument might indicate low risk while another involving other variables might show high risk for the same patient. This was obvious in a Finnish study that showed poor agreement when four different instruments were applied to identify suicidal thoughts in depressed subjects [52].

It should be pointed out that inclusion criteria varied widely from study to study. Also, even within a particular study there could be large variation in terms of psychopathology and previous history of suicidal behavior. The participation rate was not reported clearly in several of the studies, and the representativity of the study group was thus unknown.

### Strengths and limitations

The current review expands on findings of previous clinical reviews of suicide assessment instruments thanks to the stringent database search and the uniform, structured assessment of risk of bias and certainty of the evidence. Studies with high risk of bias were excluded from the meta-analyses in the current review, a procedure that can be expected to yield more conservative estimates of sensitivity and specificity. Bias has been shown to increase the risk of overestimating diagnostic accuracy [53].

A methodological consideration is that the choice of limit for sensitivity applied in the current review was arbitrary; 80% could be considered low. An instrument with a sensitivity under 80% would as mentioned above fail to detect one out of five patients with the studied outcome. A specificity exceeding 50% is also a low demand, only slightly better than a random result. However, we chose a lower limit considering the severity of the outcomes studied.

The fact that studies published after December 2014 are not included in the analyses is a limitation. A further consideration is that only some of the instruments had sufficient data to allow for a meta-analysis (Figs 2 and 3), and in most of these cases data were from two studies only, rendering it difficult to allow for adjustments. A larger number of studies would allow for stratification by treatment setting, which might be used as a proxy marker of risk level.

It is uncertain whether the results presented in this review are transferable to populations with lower risk. The latter is a relevant question from a public health standpoint as suicide decedents often visit their primary care providers during the period prior to suicide. For example, three out of four suicide decedents had contact with primary care providers in the US, UK and Scandinavia during the year preceding death [2].

Ideally, the diagnostic accuracy should be tested in controlled trials where patients randomly are assigned to risk assessment or no risk assessment. Such a design is not an option since patients who are potentially suicidal need to be evaluated and those identified as being at high risk are offered other interventions than their peers. This may in itself influence future risk of suicide, thus altering the instrument's predictive ability. The extent to which patients in the included studies were treated with specific suicide preventive interventions was not made clear in the sample descriptions, which also limits the interpretation of findings.

### Research implications

A large number of studies had to be excluded from the present systematic review due to high level of bias, highlighting the need for enhanced rigor in study design. Future studies on the prediction of suicide ought to better characterize their study groups, and be large enough to draw age- and diagnosis-specific conclusions about the predictive validity of the instrument. Assessment tools may need to be adjusted for diagnostic entities including major depression, substance use and personality disorders. In the future, analytic models more appropriate for rare outcomes could be applied [54]. This is important as suicide is, epidemiologically speaking, a fairly rare phenomenon, even though it is ranked 14 in the list of global causes of death (WHO) [55]. When planning new studies, researchers will need to aim for shorter, more clinically relevant follow-up times [10, 56]. Such studies will require sample sizes considerably greater than most of those included in the present review. Few of the included studies provided psychometric assessments. More research is needed to determine a given instrument’s ability to generate conceptually valid and consistent data.

### Clinical implications

Most of the studies included in this review were carried out in research settings, and it remains unclear whether suicide risk instruments might improve prediction when used as a *complement* to the global clinical assessment.

In the current review, we found no scientific support for the use of suicide risk instruments for predicting suicidal acts. However, suicide risk assessment instruments may still have some value as educational aides for less experienced staff and could thus be useful from a pedagogical perspective. Patients must be given the opportunity to share their own stories and understanding of the situation [57, 58]. The addition of an instrument in the suicide risk assessment may help to elicit more information, with relevant and uniform content, if integrated into a dialogue in which the clinician is able to provide ample space for the patient’s narrative.

## Supporting information

S1 File Report in Swedish. (PDF)

S2 File Explanation of the submission and its relation to the Swedish report. (DOCX)

S3 File Permission from the Swedish agency for health technology assessment. (PDF)

S4 File PRISMA 2009 checklist. (DOC)

S1 Table Search strategy. (DOCX)

S2 Table Excluded articles. (DOCX)

S3 Table Characteristics of the included studies. (DOCX)
