# Supplementary material for: Instruments for the assessment of suicide risk: A systematic review evaluating the certainty of the evidence
Source: PLoS One. 2017 Jul 19;12(7):e0180292. doi: 10.1371/journal.pone.0180292 (PMC5517300; doi:10.1371/journal.pone.0180292)
Supplement: S1 File — (PDF) [file pone.0180292.s001.pdf]

Instrument för bedömning av suicidrisk

En systematisk litteraturöversikt

September 2015

SBU • Statens beredning för medicinsk och social utvärdering

Swedish Agency for Health Technology Assessment and Assessment of Social Services

SBU utvärderar sjukvårdens och socialtjänstens metoder

SBU, Statens beredning för medicinsk och social utvärdering, är en statlig myndighet som utvärderar hälso- och sjukvårdens och socialtjänstens metoder. SBU analyserar metodernas nytta, risker och kostnader och jämför vetenskapliga fakta med praxis inom svensk vård och socialtjänst. Målet är att ge ett bättre beslutsunderlag för alla som avgör hur vården och omsorgen ska utformas.

Välkommen att läsa mer om SBU:s rapporter och verksamhet på www.sbu.se.

Denna utvärdering publicerades år 2015. Resultat som bygger på ett starkt vetenskapligt underlag fortsätter vanligen att gälla under en lång tid framåt. Andra resultat kan ha hunnit bli inaktuella. Det gäller främst områden där det vetenskapliga underlaget är otillräckligt eller begränsat.

Denna rapport (nr 242) kan beställas från Strömberg distribution
Telefon: 08-779 96 85 • Fax: 08-779 96 10 • E-post: sbu@strd.se

Grafisk produktion av Anna Edling, SBU
Tryckt av Elanders Sverige AB, Mölnlycke, 2015
Rapportnr: 242 • ISBN 978-91-85413-86-7 • ISSN 1400-1403

Citera denna rapport: SBU. Instrument för bedömning av suicidrisk. En systematisk litteraturöversikt. Stockholm: Statens beredning för medicinsk och social utvärdering (SBU); 2015. SBU-rapport nr 242. ISBN 978-91-85413-86-7.

Instrument för bedömning av suicidrisk

En systematisk litteraturöversikt

Projektgrupp

Sakkunniga

Bo Runeson (ordförande)
Tobias Edbom
Ingalill Jildevik Adamsson
Anna Lindblad
Margda Waern

SBU

Jenny Odeberg (projektledare)
Elisabeth Gustafsson (projektadministratör)
Harald Gyllensvärd (hälsoekonom)
Hanna Olofsson (informationsspecialist)
Agneta Pettersson (biträdande projektledare)

Externa granskare

Lil Träskman Bendz
Øivind Ekeberg
Sandra af Winklerfelt Hammarberg

SBU • Statens beredning för medicinsk och social utvärdering

Swedish Agency for Health Technology Assessment and Assessment of Social Services

Innehåll

SBU:s sammanfattning och slutsatser **9**

1. Inledning **17**
Syfte 17
Målgrupper 17

2. Bakgrund **19**
Begrepp och definitioner 19
Intervjuinstrument och skattningsskalor 22
Hur testar man tillförlitligheten hos ett instrument? 23
Beskrivning av de olika instrumenten 25

3. Metodbeskrivning **29**
Frågeställningar 29
Urvalskriterier 29
Avgränsning 31
Metodik för urval av studier 31
Litteratursökning 31
Granskning och kvalitetsbedömning av litteraturen 32
Metoder för sammanvägning av resultat 33
Instrumentens tillförlitlighet 34
Det vetenskapliga underlagets styrka 35
GRADE-bedömning – specifikt för detta projekt 37

4. Resultat av granskningen: suicid **39**
Sammanfattning av resultaten 40
Resultat 41
Instrument använda i psykiatrisk specialistvård 41
Instrument använt i akutmedicinsk vård för triagering till psykiatrisk bedömning 48
Instrument använt för depressionsskattning i primärvård 50

5. Resultat av granskningen: suicidförsök 53
Sammanfattning av resultaten 54
Resultat 56
Instrument använda i psykiatrisk specialistvård 56
Instrument använda i akutmedicinsk vård för triage till psykiatrisk bedömning 65
Primärvård 71

6. Resultat av granskningen: patientsäkerhet och patientens upplevelse 73
Blir patientsäkerheten bättre om bedömningsinstrument används? 73
Hur upplever patienter användningen av bedömningsinstrument? 74

7. Hälsoekonomi 75

8. Etiska aspekter 77
Relationen patient – bedömare kan påverkas 77
Värde för diagnostiken 79
Kvalitetssäkring 80
Konsekvens för vården 80

9. Praxis 81
Användning av suicidskattningsinstrument i Sverige 81
Analys av Lex Maria-anmälningar om suicid 81
Enkätundersökningar 83

10. Diskussion 87
Metodfrågor 87
Deltagare 87
Instrumenten 88
Utfall 89
Uppföljningstid 89
Krav på tillförlitlighet 89
Diskussion av fynden 90
Jämförelser med andra systematiska översikter 91

11. Konsekvenser och kunskapsluckor **93**
Praktiska konsekvenser 93
Forskningskonsekvenser 95

12. Tabeller **97**

13. Ordförklaringar och förkortningar **131**

14. Personer som medverkat till denna rapport **135**
Projektgrupp 135
Kansli 136
Externa granskare 136
Referensgrupp 137
Bindningar och jäv 137

15. Referenser **139**

Bilaga 1. Sökstrategier
Publicerad på www.sbu.se/242

Bilaga 2. Exkluderade studier
Publicerad på www.sbu.se/242

Bilaga 3. Bedömningsmallar
Publicerad på www.sbu.se/242

Bilaga 4. Studier av låg kvalitet
Publicerad på www.sbu.se/242

Bilaga 5. Praxisundersökning
Publicerad på www.sbu.se/242

SBU:s sammanfattning och slutsatser

SBU • Statens beredning för medicinsk och social utvärdering

Swedish Agency for Health Technology Assessment and Assessment of Social Services

SBU:s sammanfattning och slutsatser

I Sverige dör årligen cirka 1 500 personer i suicid, vilket är en medelhög nivå ur ett internationellt perspektiv. Psykosociala problem och psykiska störningar som till exempel depression eller alkoholberoende, är vanliga riskfaktorer. Det är viktigt att kunna identifiera vilka patienter som har förhöjd risk att suicidera men beteendet är komplext och svårt att förutsäga och forskare har därför utvecklat intervju- och skattningsinstrument för att underlätta och förbättra den kliniska bedömningen av risk för suicid.

Syftet med denna rapport har varit att utvärdera sådana instrument. För att ett instrument skulle anses vara tillräckligt känsligt, sattes en minimigräns på sensitivitet 80 procent men en lägre minimigräns för specificitet, 50 procent.

Slutsatser

- ▶ Det saknas vetenskapligt stöd för att något skattningsinstrument har tillräcklig tillförlitlighet för att användas för att förutsäga framtida suicid (80 % sensitivitet, 50 % specificitet). Instrument med hög sensitivitet kan dock fylla en funktion som pedagogiskt stöd för mindre erfarna kliniker.

- ▶ Det finns starkt vetenskapligt stöd för att skattningsinstrumentet SAD PERSONS Scale har mycket låg sensitivitet; en majoritet av personer som senare gör någon suicidhandling identifieras inte.

- ▶ Det behövs forskning som utvärderar om bedömningen av suicidrisk förbättras av att instrument används som komplement till den kliniska bedömningen. Idag saknas det studier.

- Det behövs mer forskning för att klarlägga vilken tillförlitlighet de vanligen använda skattningsinstrumenten SUAS och C-SSRS har.

- Det saknas studier som utvärderar om suicidfrågan i depressionsskalan MADRS kan predicera suicidhandling.

Metod för den systematiska översikten

Den systematiska översikten genomfördes enligt SBU:s metodik. Studiedeltagarna kunde vara personer i alla åldrar som sökt vård. Deltagarnas risk för framtida suicidhandling bedömdes med ett instrument och vid uppföljning analyserades i vilken utsträckning som instrumentet kunnat förutse vilka personer som skulle göra suicidförsök eller fullbordat suicid. Vid bedömning av det samlade vetenskapliga underlaget tillämpades GRADE, som klassificerar evidensen i fyra nivåer (starkt, måttligt starkt, begränsat och otillräckligt underlag).

Resultat

Totalt identifierades 13 instrument som bedömde risken för suicidförsök och 9 instrument som bedömde risken för suicid. Endast ett instrument, suicidfrågan i Patient Health Questionnaire-9 (PHQ- 9), hade utvärderats i primärvård. Studiepopulationerna för övriga instrument var ungdomar och vuxna som antingen just genomfört en självskadehandling eller som hade depression eller ångestsyndrom. Tabell 1 sammanfattar resultaten för de instrument där det fanns tillräckligt med studier för att kunna bedöma det vetenskapliga stödet. Som framgår av tabellen fanns det inte något instrument som uppfyllde de uppställda kraven på sensitivitet och specificitet.

För Suicide Assessment Scale (SUAS) och Columbia Suicide Severity Rating Scale (C-SSRS) fanns det inte tillräckligt med studier för att kunna bedöma tillförlitligheten, det vill säga det vetenskapliga underlaget var otillräckligt. Vi identifierade inga studier som hade utvärderat suicidfrågan i MADRS.

Tabell 1 Sammanfattande resultattabell.

Instrument	Utfall	Sensitivitet (95% KI)	Specificitet (95% KI)	Evidens-styrka	Använd-barhet
**Population: patienter med depression/ångestsjukdomar**					
BHS	Suicid	89 (78; 95)	42 (40; 43)	Måttligt stark ⊕⊕⊕○	För låg specificitet
SSI-C	Suicid	53 (34; 72)	83 (82; 84)	Begränsad ⊕⊕○○	För låg sensitivitet, risk att missa suicid-benägna personer
SSI-W	Suicid	80 (61; 92)	78 (77; 79)	Måttligt stark ⊕⊕⊕○	Det lägre konfidens-intervallet för sensitivitet är för lågt
PHQ-9<br><br>Frågan om suicid	Suicid	80 (66; 91)	70 (70; 71)	Begränsad ⊕⊕○○	Det lägre konfidens-intervallet för sensitivitet är för lågt
	Suicidförsök	78 (74; 81)	70 (70; 71)	Måttligt stark ⊕⊕⊕○	Något låg sensitivitet men gränsfall
**Population: patienter vid psykiatrisk akutvård**					
SAD PERSONS Scale	Suicidförsök*	15 (8; 24)	97 (96; 98)	Stark ⊕⊕⊕⊕	För låg sensitivitet, hög risk att missa suicid-benägna
Modified SAD PERSONS Scale	Suicidförsök	29 (19; 40)	89 (88; 90)	Begränsad ⊕⊕○○	För låg sensitivitet, hög risk att missa suicid-benägna
MINI Suicidmodul	Suicidförsök	61 (47; 73)	75 (69; 80)	Begränsad ⊕⊕○○	För låg sensitivitet

Tabellen fortsätter på nästa sida

Tabell 1 fortsättning

Instrument	Utfall	Sensitivitet (95% KI)	Specificitet (95% KI)	Evidens-styrka	Använd-barhet
**Population: patienter som skadat sig själva/utfört ett suicidförsök**					
MSHR	Suicidförsök	97 (96; 97)	20 (20; 21)	Stark ⊕⊕⊕⊕	För låg specificitet
ReACT	Suicid	90 (82; 95)	17 (18; 18)	Måttligt stark ⊕⊕⊕○	För låg specificitet
	Suicidförsök	94 (93; 94)	24 (23; 25)	Måttligt stark ⊕⊕⊕○	För låg specificitet
SoS-4	Suicidförsök	90 (86; 93)	17 (15; 19)	Stark ⊕⊕⊕⊕	För låg specificitet
SIS	Suicid	76 (62; 87)	49 (47; 51)	Begränsad ⊕⊕○○	Något låg specificitet

* En av studierna genomfördes i en population med självskadebeteende.

MSHR = Manchester self harm rule; **ReACT** = Recent self-harm in the past year – Alone or homeless, Cutting used as a method of harm, Treatment for a psychiatric disorder; **SIS** = Beck's suicide intent scale; **SoS-4** = Södersjukhuset self-harm rule

Det finns tre aspekter som kan påverka tillförlitligheten i resultaten. Instrumenten är utvärderade i en population, vanligen personer som begått självskade- eller suicidhandling och därmed har hög risk att upprepa beteendet. Det är osäkert om resultaten är överförbara till populationer med lägre risk. Ytterligare en aspekt är de långa uppföljningstiderna, ofta flera år medan det är kliniskt mera relevant med resultat från kort uppföljningstid. Dessutom kommer de personer som identifierats ha hög risk för suicid att få andra insatser än de som identifierats ha låg risk, vilket i sig kan påverka framtida suicid och därmed instrumentets sensitivitet/specificitet.

Etiska aspekter

Även om det saknas evidens för de granskade instrumentens prediktionsförmåga, kan de vara användbara ur en pedagogisk synvinkel. Om de integreras i ett samtal där intervjuaren framför allt är angelägen om att ge utrymme för patientens beskrivning och uppfattning av situationen kan de fungera som ett hjälpmedel för att få mer information med kvalitetssäkrat innehåll.

Konsekvenser

Det finns inte några instrument som har tillräcklig tillförlitlighet för att användas för att predicera suicidhandlingar. Granskningen visar också att instrumentet SAD PERSONS Scale inte är tillförlitligt och inte bör användas i dess nuvarande form. Instrumenten kan ha ett visst värde som pedagogiskt stöd för att lära ny eller mindre erfaren personal att fråga efter viktiga riskfaktorer för suicidhandling i patientsamtalet.

Man bör betona att resultaten inte ifrågasätter värdet av att man gör en klinisk suicidriskbedömning. Det är oklart om skattningsinstrument kan förbättra prediktionen av suicidhandlingar när instrumenten används som komplement till klinisk bedömning, då det idag saknas studier. Det är angeläget med forskning för att klarlägga tilläggsvärdet med instrument. De kan behövas konstrueras så att de är tillämpliga för olika ålders-, köns- eller diagnosgrupper.

Eftersom det inte går att bedöma tillförlitligheten hos SUAS och C-SSRS som tillhör de ofta rekommenderade instrumenten i Sverige behövs studier som utvärderar dem.

1. Inledning

Suicidriskbedömningar ingår som en viktig del i omhändertagandet av patienter i akut psykiatrisk vård, men också vid fortsatt slutenvård och uppföljning i öppenvård. I akutmedicinsk vård är det också viktigt att värdera risken för suicid hos personer som inkommer efter någon form av självskadehandling.

I kliniska riktlinjer samt regionala och lokala vårdprogram rekommenderas ofta särskilda intervjuinstrument eller skattningsskalor. Vid tillsynsbedömningar enligt Lex Maria efterfrågas bedömning och dokumentation av suicidrisk med dessa instrument. Men evidensen är oklar i den betydelsen att det inte har kunnat bevisas om dessa instrument kan förutsäga suicidförsök eller fullbordat suicid. Flera av dessa instrument har använts i decennier och kunskapen efterfrågas bland kliniskt verksamma.

Syfte

Syftet med denna rapport är att bistå hälso- och sjukvården med kunskap om intervjuinstrument och skattningsskalor för suicidriskbedömning samt att påvisa forskningsluckor och utvecklingsbehov.

Målgrupper

Rapporten vänder sig till personal och beslutsfattare inom landstingens och kommunernas hälso- och sjukvård samt till berörda myndigheter.

2. Bakgrund

Begrepp och definitioner

Suicid är ett globalt folkhälsoproblem som har uppmärksammats av världshälsoorganisationen, WHO. Någonstans i världen avlider en person till följd av suicid var 40:e sekund [1]. Förekomsten av suicid i Sverige ligger på en medelhög nivå i ett internationellt perspektiv. Ungefär 1 500 människor årligen avlider till följd av suicid vilket motsvarar drygt 20 per 100 000 invånare.

Antalet suicid i Sverige minskade relativt kraftigt under 1980 och 90-talet, men har därefter legat på ungefärligt oförändrad nivå (Figur 2.1). Denna minskning gäller dock inte för åldersgruppen ungdomar och unga vuxna (15–24 år). Antalet suicidförsök minskade kraftigt under senare hälften av 1990-talet, men ökade sedan igen under 2000-talets början. Bland de yngre ökade antalet suicidförsök mellan 1990-talets slut och fram till 2008, för att senare minska.

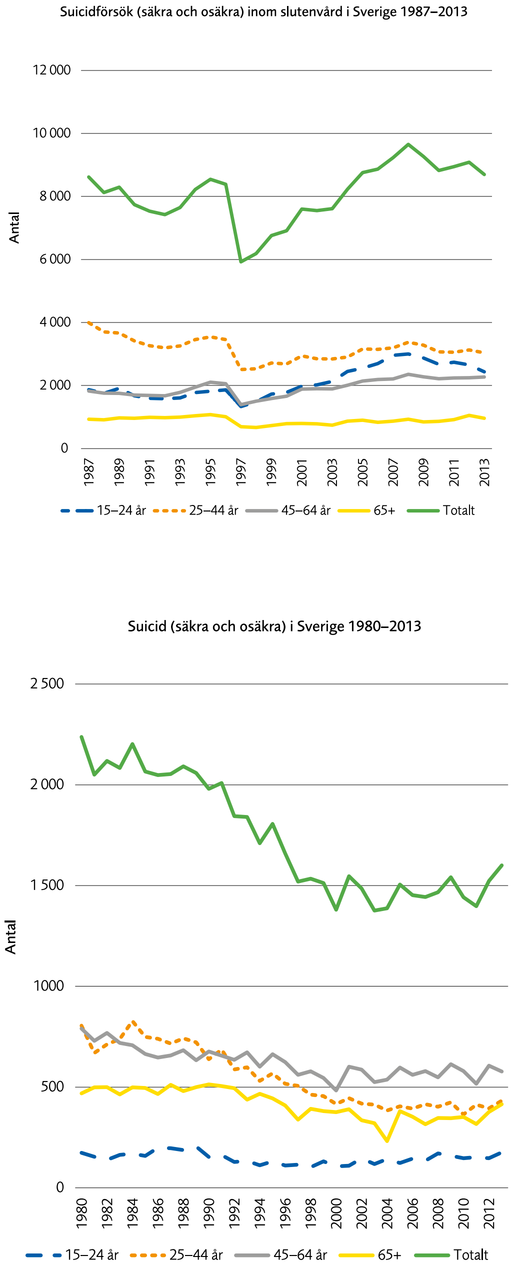

Figur 2.1 Statistik över antalet suicidförsök och suicid i Sverige, fördelat inom olika åldersgrupper samt totalt (information inhämtad från NASP:s webbplats).

I Sverige är suicid drygt två gånger vanligare hos män än hos kvinnor. Bland unga och yngre medelålders män (15–44 år) är suicid den vanligaste dödsorsaken och bland kvinnor den näst vanligaste efter tumörsjukdomar. Äldre män har höga suicidtal både i Sverige och internationellt.

Sjukvården utgör en viktig arena för suicidpreventiva insatser, då personer som tar sina liv ofta har sökt vård en kort tid dessförinnan. Framför allt sker dessa kontakter inom primärvård och medicinsk specialistvård [1]. Personer som vårdas i samband med suicidförsök är en grupp med särskilt förhöjd risk för att senare utföra suicid. Risken beror i hög utsträckning på om det samtidigt finns psykisk störning och särskilt hög är risken vid depression, bipolär sjukdom och schizofreni [2]. En fjärdedel av de som avlider genom suicid i Sverige har varit inlagda på psykiatrisk klinik under det sista levnadsåret [3].

En viktig del i det initiala skedet av behandlingen är bedömningen om det finns en suicidal avsikt och risk för suicidhandling. Störst klinisk relevans har bedömningen om den sker i omedelbar anslutning eller kort tid efter patientmötet. Detta är en svår bedömning i en ofta krisartad patientsituation, där förmågan att skapa förtroende hos patienten är viktig. Bedömningen kan vara en del av en kortare intervju eller en längre mer djupgående intervju där kvaliteten av bedömningen påverkas av bedömarens utbildning, erfarenhet och engagemang. Suicidriskbedömningen har dock en avgörande betydelse för den fortsatta vården och behandlingen. Den styr hur vården ska planeras i flera avseenden; kräver situationen att patienten får vård inneliggande? bör extra försiktighetsåtgärder som särskild tillsyn vidtas? om uppföljning sker i öppenvård, hur tät kontakt behövs?

Faktaruta 2.1 Begrepp och definitioner.

Suicid (självmord) avser självtillfogad skada som leder till döden.

Suicidalt beteende är ett samlingsbegrepp som omfattar självskadehandlingar, suicidförsök och suicid, men används ibland så att det också omfattar suicidtankar.

Suicidförsök (självmordsförsök) avser självtillfogade skador som inte leder till döden, men där det kan ha funnits en suicidavsikt. Termen används också för handlingar utan suicidavsikt.

Självskada motsvaras i brittisk terminologi av "deliberate self-harm" (DSH). En bred term som omfattar självskadehandlingar oavsett suicidavsikt eller inte. I detta begrepp ingår även förgiftningar och självskadehandlingar med andra metoder.

Icke-suicidal självskada motsvaras i amerikansk terminologi av "non-suicidal self injury" (NSSI). Det är en självskada utan suicidal avsikt som oftast gäller skärskador eller försök att bränna sig eller på annat sätt orsaka skador i huden.

Suicidnära avser personer som nyligen (under det senaste året), har utfört en självskada eller haft allvarliga suicidtankar och där suicidrisk bedöms föreligga under den närmaste tiden. Begreppet omfattar även personer som utan att ange allvarliga suicidtankar på grund av omständigheter, bedöms vara i farozonen för suicid.

Suicidal process beskriver utvecklingen från den första allvarliga tanken på suicid till eventuellt suicidförsök och fullbordat suicid.

Intervjuinstrument och skattningsskalor

Suicidalt beteende är komplext och svårt att förutsäga. I syfte att underlätta och förbättra kliniska bedömningar har forskare utvecklat och testat ett antal olika intervjuinstrument och riskbedömningsskalor. Det finns flera olika typer av instrument och några av de vanligast förekommande presenteras i Tabell 2.1.

Tabell 2.1 Olika typer av instrument.

Instrument för suicidriskbedömning, (används framför allt inom psykiatrisk specialistvård)	Instrument som används inom akutmedicinsk vård för triagering till psykiatrisk bedömning	Depressionsinventorier (används bland annat inom primärvård)
SAD PERSONS Scale	Manchester Self Harm Rule (MSHR)	Patient Health Questionnaire (PHQ-9)
International Neuropsychiatric Interview (MINI)	Recent self-harm in the past year – Alone or homeless, Cutting used as a method of harm, Treatment for a psychiatric disorder (ReACT)	Montgomery Åsberg Depression Rating Scale (MADRS)
Suicide Assessment Scale (SUAS)	Södersjukhuset Self-Harm Rule (SoS-4)	Beck Depression Inventory (BDI)
Beck's Suicide Intent Scale (SIS)		
Beck's Scale for Suicide Ideation (SSI-C och SSI-W)		
Columbia Suicide Severity Rating Scale (C-SSRS)		

Hur testar man tillförlitligheten hos ett instrument?

Forskare har genom åren testat olika instruments förmåga att identifiera personer som kommer att göra ett suicidförsök eller fullbordat suicid. Ett bra skattningsinstrument ska vara tillräckligt känsligt för att missa så få som möjligt av dem som senare kan komma att göra ett suicidförsök/suicid; det ska ha hög sensitivitet. Samtidigt ska instrumentet utlösa så få ”falska alarm” som möjligt, det vill säga så att individer med låg risk kan identifieras med hög träffsäkerhet; instrumentet ska ha så hög specificitet som möjligt. Eftersom sensitivitet och specificitet är relaterade till varandra är det oftast svårt att uppnå både hög sensitivitet och hög specificitet samtidigt. Tröskelvärdet (cut-off), för ett instrument definierar gränsen mellan ”sjuk” (suicidal) och ”frisk” (icke-suicidal). Ett lågt tröskelvärde innebär att många uppfyller kriteriet och att sensitiviteten blir hög, men om tröskelvärdet höjs minskar sensitiviteten medan specificiteten ökar (Faktaruta 2.2).

Sensitivitet och specificitet mäts i relation till en referensmetod som i så hög grad som möjligt ska kategorisera friska och sjuka korrekt. Det finns flera typer av referensmetoder (t ex andra instrument), som är väl validerade. För suicidriskbedömning saknas validerade instrument som kan fungera som referensmetod. Alternativet blir då att göra en uppföljande undersökning av studiedeltagarna samt att beräkna i hur hög grad resultaten från skattningen kan förutsäga suicidhandling (suicidförsök eller fullbordat suicid).

Faktaruta 2.2 Termer inom diagnostiska tester.

Sant positiva = sjuka klassificeras som sjuka (a)
Sant negativa = friska klassificeras som friska (d)
Falskt positiva = friska klassificeras som sjuka (b)
Falskt negativa = sjuka klassificeras som friska (c)

Ovanstående utfall brukar redovisas i en fyrfältstabell (Figur 2.2) och från dessa värden beräknas sensitivitet och specificitet.

Sensitivitet = Sannolikheten för positivt testresultat när man har sjukdomen = a/(a+c).

Specificitet = Sannolikheten för negativt testresultat när man är frisk = d/(b+d).

Prediktivt värde
Positivt prediktivt värde (PVV) = Sannolikheten för att ett positivt testresultat stämmer.

Andelen sant positiva av samtliga positiva resultat (a/(a+b)).

Negativt prediktivt värde (PVV) = Sannolikheten för att ett negativt testresultat stämmer.

Andelen sant negativa av samtliga negativa resultat (d/(c+d)).

Hur stort det prediktiva värdet är beror inte bara på diagnosmetodens prestanda utan också på hur vanlig sjukdomen/utfallet är.

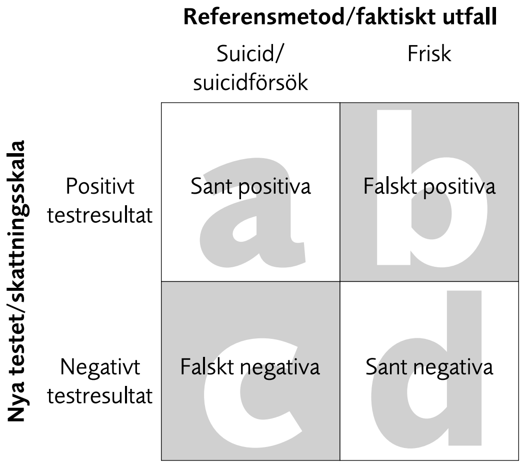

Figur 2.2 Fyrfältstabell.

Beskrivning av de olika instrumenten

I denna rapport identifierades 16 olika instrument. Nedan följer en kortfattad beskrivning av de instrument som ingår i översikten.

Instrument för suicidriskbedömning (använda inom psykiatrisk specialistvård)

- **Beck's Hoplessness Scale (BHS)** utvecklat för vuxna mellan 17 och 80 års ålder och består av 20 sant eller falskt-frågor. Instrumentet mäter de tre specifika komponenterna motivation, affektiva och kognitiva faktorer hos deprimerade patienter.

- **Beck's Suicide Intent Scale (SIS)** används för bedömning efter ett suicidförsök. Skalan inkluderar 15 frågor. De första åtta berör objektiva omständigheter kring försöket inklusive förberedelser. Återstående sju frågor berör patientens egna tankar och känslor i samband med försöket. Varje fråga graderas 0–2 poäng med en högsta totalsumma på 30 poäng.

- **Columbia Suicide Severity Rating Scale (C-SSRS)** består av fyra subskalor. Suicidtankarnas svårighetsgrad mäts på en 5-gradig ordinalskala (1 = dödsönskan, 2 = ospecifika suicidtankar, 3 = suicidtankar med metod, 4 = suicidal avsikt (intention), 5 = suicidal avsikt (intention) med en plan). Suicidtankarnas intensitet beskrivs med fem frågor, där varje mäts på en 5-gradig ordinalskala: frekvens, duration, kontrollerbarhet, om det finns avskräckande faktorer och vilket skäl som finns till suicidtankarna. Faktiska beteenden beskrivs på den tredje delskalan som är en nominalskala som omfattar 1) faktiskt försök, 2) uppgivet försök, 3) avbrutet försök, 4) förberedande beteende och 5) självskada utan suicidal intention. Den medicinska risken att skadas eller dö vid faktiska försök graderas med en 6-gradig ordinalskala. Om ingen skada inträffade värderas den potentiella medicinska skadan vid använd suicidförsöksmetod på en 3-gradig ordinalskala.

- **Edinburgh Risk of Repetition Scale (ERRS)** är en skala för både kliniskt bruk och forskning som består av 11 frågor. Frågorna ger både sociodemografisk och klinisk information. Skalan avser att identifiera hög, medel och låg risk för upprepade suicidförsök inom ett år.

- **Implicit Association Test (IAT)** är ett kort datorbaserat test, vilket mäter personers underförstådda associationer till suicid, död och självskada.

- **MINI - International Neuropsychiatric Interview** är en standardiserad diagnostisk intervju med syftet att fastställa psykiatriska störningar i enlighet med DSM- och ICD-systemen. I den diagnostiska intervjun finns ett avsnitt (B) om suicidtankar och suicidhandlingar som rör både den närmaste månaden samt livstid.

- **SAD PERSONS Scale** utvecklades i början av 1980-talet som ett stöd i suicidriskbedömningar för läkarstudenter och icke-psykiatrisk personal. Tio välkända riskfaktorer för suicid hos vuxna ingår. Vissa är objektiva (t ex patientens ålder och kön), medan andra är subjektiva (t ex förlust av rationellt tänkande).

- **Modified SAD PERSONS Scale (MOD SAD)** är en modifierad version av SAD PERSONS Scale där frågan om sjukdom ersatts med rapporterad suicidavsikt. Dessutom använder skalan ett modifierat poängsystem (poängsumma 0–14), där vissa faktorer viktas.

- **Scale for Suicide Ideation – Current/Worst (SSI-C/SSI-W)** är skattningsskalor som består av 19 frågor uppdelade på fyra delskalor: patientens attityd till att leva eller dö; suicidtankarnas karaktär; det tänkta försökets karaktär samt förberedelser inför försöket. SSI-C mäter aktuella suicidtankar medan SSI-W mäter suicidtankar när de varit som mest uttalade under livet.

- **Suicide Assessment Scale (SUAS)** utvecklades för att mäta suicidrisk över tid. Tjugo frågor ingår vilka graderas 0–4, vilket ger en summa på 0–80 poäng. Skalan täcker fem områden: affekter; kroppsliga tillstånd; kontroll och coping; emotionell reaktivitet; suicidtankar och -beteenden. Högre poängsumma indikerar högre allvarlighetsgrad. Skalan finns även i självskattningsversion (SUAS-S).

- **Suicide Probability Scale (SPS)** mäter ångest i 20 olika sociala prestationssituationer. Skalan är av Likerttyp med fem skalsteg som sträcker sig från 0 (stämmer inte alls) till 4 (stämmer precis).

Instrument använda inom akutmedicinsk vård för triagering till psykiatrisk bedömning

- **Manchester Self Harm Rule (MSHR)** är ett kort screeninginstrument för att identifiera patienter med möjlig suicidrisk inom somatisk akutvård. Instrumentet består av fyra frågor vilka fokuserar på om det finns eller funnits tidigare självskada, tidigare psykiatrisk behandling, om bensodiazepin använts vid det aktuella försöket och om det finns pågående psykiatrisk kontakt. Om en av dessa föreligger bedöms det finnas skäl för psykiatrisk bedömning.

- **ReACT** (Recent self-harm in the past year – Alone or homeless, Cutting used as a method of harm, Treatment for a psychiatric disorder), är ett instrument utformat som ett beslutsstöd och omfattar

fyra faktorer för att identifiera risk för suicid eller suicidförsök. Dessa faktorer är: en aktuell självskada (senaste året); att vara ensamboende eller hemlös; att skärning används som självskademetod eller att ha behandling för pågående psykisk störning. Om en av dessa faktorer föreligger bedöms det finnas skäl för en psykiatrisk bedömning.

- **Södersjukhuset Self-Harm Rule (SoS-4)** är en modifikation av screeninginstrumentet MSHR och består av fyra frågor. De ingående faktorerna är kön, om patienten har antidepressiv behandling, om det finns tidigare självskada och om patienten har en pågående psykiatrisk kontakt. Instrumentet klassificerar patienter i hög eller låg risk för upprepade suicidförsök.

Depressionsinventorier (använda bl a inom primärvård)

- **Patient Health Questionnaire (PHQ-9)** är ett bedömningsformulär till stöd för diagnostisering av depression (”riktad screening”) samt för att värdera svårighetsgraden av depressionen. Instrumentet består av nio frågor varav en fråga belyser döds- och suicidtankar. Patienten får själv skatta sin sinnesstämning.

- **Beck Depression Inventory (BDI)** är ett bedömningsinstrument som mäter grad av depressivitet. Instrumentet innehåller 21 flervalsfrågor där patienten själv får uppskatta sin sinnesstämning under den senaste veckan, vilka skattas på en fyrgradig skala (0–3 poäng). BDI mäter grad av depressivitet.

3. Metodbeskrivning

En systematisk översikt innebär att man identifierar, samlar in och väger samman forskningsresultat från flera olika studier. Processen kännetecknas av systematik och öppenhet för att minska risken att resultaten snedvrids. Målet är att säkerställa tillförlitliga resultat. I detta kapitel beskrivs hur vi gått tillväga när underlaget för översikten tagits fram. En mer detaljerad beskrivning av SBU:s projektprocess återfinns i handboken Utvärdering av metoder i hälso- och sjukvården [4].

Frågeställningar

- Finns det bedömningsinstrument som på ett tillförlitligt sätt kan förutsäga nya suicidala handlingar hos individer som vårdas för suicidförsök?
- Finns det bedömningsinstrument som på ett tillförlitligt sätt kan förutsäga suicidala handlingar hos individer vid annan vårdkontakt?
- Blir patientsäkerheten bättre om bedömningsinstrument används?
- Hur upplever patienter användningen av bedömningsinstrument för suicidrisk?
- Vilka etiska faktorer finns det att beakta?

Urvalskriterier

Följande kriterier fastställdes för urval av studier:

- **Population:** Kliniska populationer (personer som sökt vård) i alla åldrar, där man utvärderar risken för suicidhandling eller suicid. Screening i den allmänna befolkningen ingår inte.

- **Indextest:** Bedömningsinstrument som är avsedda att värdera intention eller att bedöma risk för suicidhandling. Enskilda frågor från andra bedömningsinstrument som är avsedda att karakterisera graden av suicidrisk till exempel MADRS, MINI, MINI-KID. Neurokognitiva test för att karakterisera graden av suicidrisk, till exempel IAT (death–suicide implicit association task). Instrument som identifieras via litteratursökningen eller som används i Sverige. Inga begränsningar avseende tröskelvärden.

- **Referenstest:** Det faktiska utfallet suicid eller suicidförsök/handling.

- **Utfallsmått:** Grad av överensstämmelse mellan bedömningsinstrumentets resultat och faktiskt utfall suicid och suicidhandling: sensitivitet och specificitet.

  Där det är möjligt ska resultatet särredovisas för kön, ålder och bakomliggande sjukdom. Studier där resultaten redovisas bristfälligt, vilket omöjliggör metaanalys, exkluderas.

- **Studiedesign:** Prospektiva longitudinella studier (kohortstudier) för frågan om tillförlitlighet och prediktionsförmåga. Randomiserade kontrollerade studier, RCT, för frågan om patientsäkerheten förbättras. Kvalitativa studier för patientupplevelser. Inga begränsningar i studiedesign för övriga frågor.

Avgränsning

Uppföljningstid och studiestorlek. Både korta och långa uppföljningstider. Minst 50 individer för kvantitativa studier.

Metodik för urval av studier

Med stöd av urvalskriterierna identifierade vi och valde ut relevanta studier i tre steg:

(1) litteratursökning, (2) urval efter bedömning av studiernas sammanfattning (abstract) samt (3) urval efter att ha läst artiklarna i sin helhet.

Litteratursökning

Med utgångspunkt från projektets frågeställningar gjordes systematiska sökningar i bibliografiska databaser i nära samarbete mellan SBU:s informationsspecialist och experterna i projektgruppen. Sökningarna gjordes i PubMed (NLM), Embase (Elsevier), Cochrane Library (Wiley) och Cinahl (EBSCO). Ytterligare studier har sökts via de vetenskapliga artiklarnas referenslistor samt i översiktsartiklar. Endast studier publicerade från 1990 och framåt inkluderades. Databassökningarna gjordes i december 2013 med en uppdatering december 2014. Sökstrategierna redovisas i detalj i Bilaga 1.

Granskning och kvalitetsbedömning av litteraturen

Granskning och kvalitetsbedömning gjordes i tre faser enligt Figur 3.1.

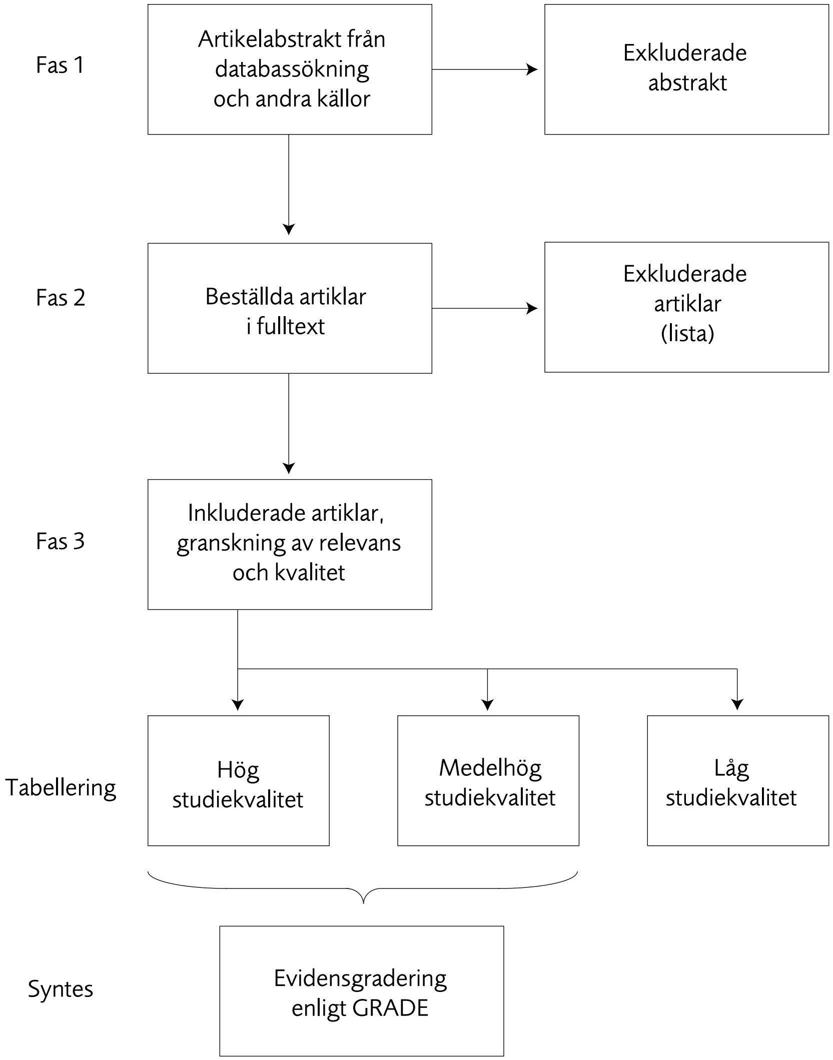

Figur 3.1 Granskningsprocessen.

Fas 1

Granskningen av artikelsammanfattningarna (abstract) gjordes av två personer, oberoende av varandra. Endast de artiklar som med säkerhet inte uppfyllde kriterierna exkluderades. Om en av granskarna rekommenderade en studie blev den inkluderad och beställd i fulltext.

Fas 2

Samma personer som i fas 1 granskade sedan fulltextartiklarna, oberoende av varandra med avseende på urvalskriterierna. Vid oenighet fördes en diskussion. Artiklar som ingen av granskarna bedömde som relevanta exkluderades (Bilaga 2). Endast studier som tydligt uppfyllde samtliga kriterier togs med.

Fas 3

Studiernas vetenskapliga kvalitet bedömdes med stöd av en modifierad version av QUADAS 2 (Bilaga 3). Endast studier med låg eller medelhög risk för bias var aktuella för att ingå i evidensbasen, det vill säga som underlag för slutsatserna. Studier med hög risk för bias (låg vetenskaplig kvalitet) redovisas därför inte i tabeller eller i figurer. Kvalitetsgranskningen genomfördes först av projektgruppens sakkunniga parvis och oberoende av varandra. Därefter diskuterades de artiklar man var oenig om, först inom läsparet och därefter i hela projektgruppen vid behov. Frågor av principiell betydelse diskuterades i hela projektgruppen. Det var inte tillåtet att bedöma egna artiklar.

Metoder för sammanvägning av resultat

Endast studier med låg eller medelhög risk för bias användes för att bedöma diagnostisk tillförlitlighet. När mer än en studie utgjorde underlag utfördes metaanalys för att väga samman studierna, dock måste studierna ha använt samma tröskelvärde för att kunna sammanvägas. För diagnostiska studier behövs två forest plots, en för sensitivitet och en för specificitet. Metaanalys innebär att man räknar fram en slags genomsnittlig effekt från de inkluderade studierna. Oftast brukar man ta hänsyn till studiernas storlek. Detta betyder att en stor studie

procentuellt sett betyder mer än en liten studie för det genomsnitt som räknas fram. Metaanalyserna i Kapitel 4 och 5 utfördes med hjälp av RevMan 5.4 (Cochrane) och Meta-DiSc 1.4 (Meta-analysis of diagnostic and screening tests, version 1.4).

Instrumentens tillförlitlighet

Det finns inga vedertagna krav (varken internationellt eller nationellt) för hur hög sensitiviteten och specificiteten måste vara för att ett instrument för suicidriskbedömning ska anses vara tillräckligt tillförlitligt för att vara användbart. Idealet är att instrumentet ska ha så hög känslighet så att alla suicidala patienter identifieras. Men då instrumentet enbart är ett komplement till den kliniska bedömningen, bygger beslutet inte enbart på formuläret och därför kan det vara acceptabelt att instrumentet missar ett litet antal patienter. Vi bedömde att det var rimligt att sätta en gräns för lägsta acceptabla sensitivitet på mer än 80 procent, vilket inkluderar ett 95-procentigt konfidensintervall. Att ställa ett lägre krav skulle medföra att instrument missade mer än var femte individ som under uppföljning hade utfallet av suicid eller suicidförsök.

Vi accepterade lägre värden för specificitet vid screening. En låg specificitet innebär att många patienter som inte är suicidala klassificeras som sådana, det vill säga är falskt positiva. Vi bedömde att en lägsta gräns på specificitet inte var nödvändig att definiera om instrumentet skulle användas i primärvård.

För att ett instrument skulle anses användbart vid prediktion inom akutmedicinsk eller psykiatrisk vård satte vi en nedre gräns på mer än 50 procent för specificiteten, vilket kan anses vara ett lågt krav. Värden högre än 50 procent specificitet innebär att instrumentet är något bättre än slumpen i att identifiera individer som inte har det angivna utfallet av suicid eller suicidförsök under uppföljningstiden.

Vid screening kan man dock även vid ett lågt värde på specificiteten värdera en skala som användbar om den har ett högt positivt prediktivt värde (PPV), då detta innebär att hög sannolikhet för att ett positivt

testresultat stämmer. Ett relativt lågt värde på specificiteten kan också accepteras om utfallet är mycket allvarligt som vid risk att dö.

Vi konsulterade en extern expert på skattningsinstrument, Simon Gilbody vid University of York i England, för att bekräfta att våra värden för lägsta sensitivitet och specificitet var rimliga.

Det vetenskapliga underlagets styrka

SBU använder det internationellt utarbetade systemet Grading of Recommendations Assessment Development and Evaluation (GRADE) för att bedöma hur tillförlitliga de sammanvägda resultaten är (Faktaruta 3.1). Tillförlitligheten uttrycks med hjälp av evidensstyrka. Ju högre slutlig evidensstyrka, desto större sannolikhet att det sammanvägda resultatet är stabilt över tid och inte påverkas av nya forskningsrön.

Faktaruta 3.1 Studiekvalitet, evidensstyrka och slutsatser.

Studiekvalitet avser den vetenskapliga kvaliteten hos en enskild studie och dess förmåga att besvara en viss fråga på ett tillförlitligt sätt.

Evidensstyrka är ett mått på hur tillförlitligt resultatet är. SBU tillämpar det internationellt utarbetade evidensgraderingssystemet GRADE. För varje effektmått utgår man i den sammanlagda bedömningen från studiernas design. Därefter kan evidensstyrkan påverkas av förekomsten av försvagande faktorer som studiekvalitet, samstämmighet, överförbarhet, precision i data och risk för publikationsbias.

Evidensstyrkan graderas i fyra nivåer:

- **Starkt vetenskapligt underlag** (⊕⊕⊕⊕).
  Bygger på studier med hög eller medelhög kvalitet utan försvagande faktorer vid en samlad bedömning.
- **Måttligt starkt vetenskapligt underlag** (⊕⊕⊕○).
  Bygger på studier med hög eller medelhög kvalitet med förekomst av försvagande faktorer vid en samlad bedömning.
- **Begränsat vetenskapligt underlag** (⊕⊕○○).
  Bygger på studier med hög eller medelhög kvalitet med kraftigt försvagande faktorer vid en samlad bedömning.
- **Otillräckligt vetenskapligt underlag** (⊕○○○).
  När studier saknas, tillgängliga studier har låg kvalitet eller där studier av likartad kvalitet visar motsägande resultat, anges det vetenskapliga underlaget som otillräckligt.

Ju starkare evidens desto mindre sannolikt är det att redovisade resultat kommer att påverkas av nya forskningsrön inom överblickbar framtid.

Slutsatser
I SBU:s slutsatser görs en sammanfattande bedömning av nytta, risker och kostnadseffektivitet.

Evidensgraderingen görs i två steg. I ett första steg ges varje sammanvägt resultat en preliminär evidensstyrka som bestäms av de ingående studierna design. För studier om diagnostisk tillförlitlighet kan även välgjorda observationsstudier utgå från en hög preliminär evidensstyrka (⊕⊕⊕⊕), som i detta fall. I nästa steg bedöms i vilken utsträckning som underlaget har svagheter beroende på metodologiska brister, heterogenitet, bristande överförbarhet till svenska förhållanden, bristande precision i det sammanvägda resultatet och risk för publikationsbias.

GRADE-bedömning – specifikt för detta projekt

Precision

Vi gjorde ett avdrag för precision om konfidensintervallet (KI) var större än 0,1 från punktestimatet och två avdrag om KI >0,2 från punktestimatet.

Överförbarhet

En studie: SBU:s bedömning är generellt att en enda studie av begränsad omfattning är otillräckligt för att bedöma evidensen. Punktestimatet är då mycket osäkert. För diagnostiska tillförlitlighetsstudier kan en enda studie dock vara tillräcklig. I detta projekt har vi gjort följande bedömning: om underlaget endast bestod av en studie och populationen utgjordes av 200 eller färre blev det vetenskapliga underlaget automatiskt otillräckligt (⊕○○○). Om däremot populationen var fler än 200 personer, gjordes avdrag för överförbarhet med ett steg. När populationen var >1 000 gjordes inget avdrag för att det endast var en studie, då populationsstorleken ansågs kompensera denna brist.

Uppföljningstid: Ju längre tid det är mellan bedömning av suicidrisk och uppföljning desto större är risken att faktorer i omgivningen har påverkat benägenhet för suicidförsök eller suicid, vilket skulle kunna påverka överförbarheten. Vi beslutade att ändå inte göra några avdrag även om studierna hade långa uppföljningstider.

4. **Resultat av granskningen: suicid**

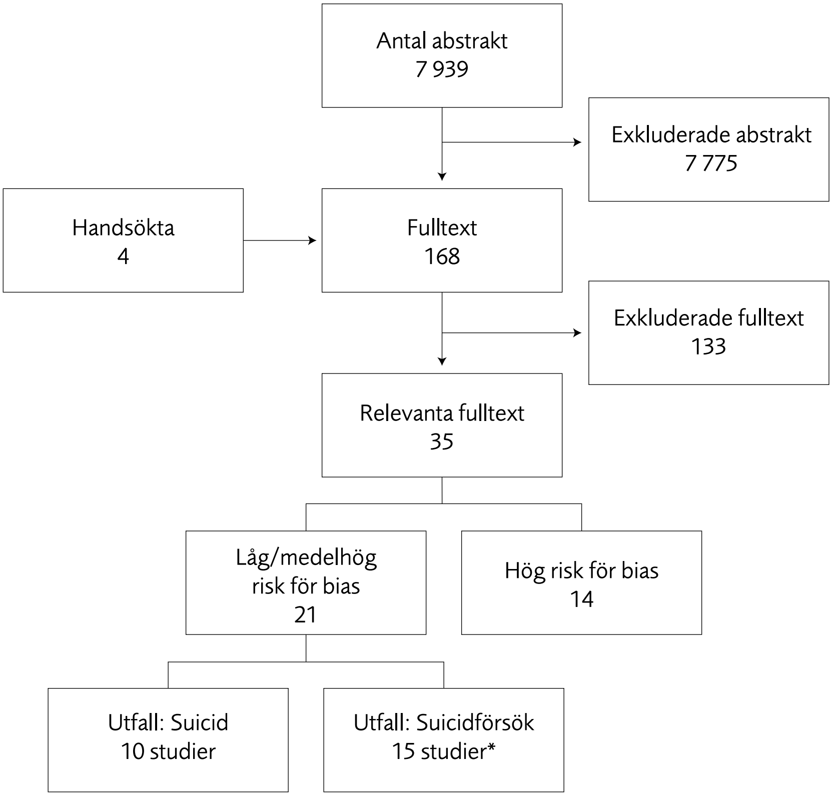

* Fyra studier hade både suicid och suicidförsök som utfall.

Figur 4.1 Flödesschema över litteratursökning. Med handplockade menas studier som tillkommit utanför litteratursökning.

Av 168 artiklar som beställdes i fulltext uppfyllde sammanlagt 35 artiklar våra urvalskriterier medan 133 artiklar exkluderades (Bilaga 2). Av de 35 relevanta studierna hade 14 hög risk för bias, och ingår därmed inte i analysen (Bilaga 4). Det var 10 av 21 studier som hade utfallsmåttet suicid och som ingår i detta kapitel.

Sammanfattning av resultaten

Resultaten är uppdelade på specialistpsykiatri, akutmedicinsk vård och primärvård. För skalor inom specialistpsykiatri har BHS och SSI-W måttligt starkt stöd (⊕⊕⊕○) för att de inte är tillförlitliga då de inte uppfyllde kraven på sensitivitet >80 procent och specificitet >50 procent (Tabell 4.1). För övriga instrument inom psykiatrisk vård är resultatet otillräckligt (⊕○○○) eller begränsat (⊕⊕○○). För det korta instrumentet för akutmedicinsk vård, ReACT, fanns ett måttligt starkt stöd (⊕⊕⊕○) för att instrumentet inte var tillförlitligt för prediktion av suicid eftersom kravet på specificitet inte var uppfyllt (Tabell 4.1). För primärvård identifierades endast en studie vilken utvärderade PHQ-9-frågan suicid (Tabell 4.1) [5]. Granskningen visade att det finns ett begränsat stöd (⊕⊕○○) för att patienter som svarar ja på denna fråga har en ökad risk för suicid under en uppföljningstid på ett år.

Tabell 4.1 Sammanfattande resultattabell över identifierade skattningsskalor och evidensstyrka för psykiatrisk specialistvård, akutmedicinsk vård och primärvård.

Instrument	Population	Sensitivitet (95% KI)	Specificitet (95% KI)	Evidensstyrka
**Psykiatrisk specialistvård**				
BDI* [6]	Depression och ångestsjukdomar, vuxna	76 (50; 93)	62 (60; 65)	Otillräcklig ⊕○○○
BHS [6–9]	Depression och ångestsjukdomar, vuxna	89 (78; 95)	42 (40; 43)	Måttligt stark ⊕⊕⊕○
BHS [10]	Nyinsjuknande med psykossjukdom, vuxna	33 (4; 78)	88 (84; 91)	Otillräcklig ⊕○○○
C-SSRS* [11]	Depression, ungdomar	100%	96%	Otillräcklig ⊕○○○
SIS [12]	Självskadehandling, ungdomar och vuxna	76 (62; 87)	49 (47; 51)	Begränsad ⊕⊕○○
SIS [13]	Allvarliga suicidförsök, ungdomar och vuxna	59 (36; 79)	77 (74; 81)	Otillräcklig ⊕○○○
SSI-C [7]	Depression och ångestsjukdomar, vuxna	53 (34; 72)	83 (82; 84)	Begränsad ⊕⊕○○

Tabellen fortsätter på nästa sida

Tabell 4.1 fortsättning

Instrument	Population	Sensitivitet (95% KI)	Specificitet (95% KI)	Evidensstyrka
SSI-W [7]	Depression och ångest-sjukdomar, Vuxna	80 (61; 92)	78 (77; 79)	Måttligt stark ⊕⊕⊕○
**Akutmedicinsk vård**				
ReACT [14]	Självskadehandling, ungdomar och vuxna	90 (82; 95)	17 (18; 18)	Måttligt stark ⊕⊕⊕○
**Primärvård**				
PHQ-9 [5]	Depression, ungdomar och vuxna	80 (66; 91)	70 (70; 71)	Begränsad ⊕⊕○○

* Endast en studie och antalet deltagare var lägre än 200. Detta gör att det vetenskapliga underlaget automatiskt blir otillräckligt (⊕○○○).

BDI = Beck depression inventory; **BHS** = Beck's hoplessness scale; **C-SSRS** = Columbia suicide severity rating scale; **PHQ-9** = Patient health questionnaire; **ReACT** = Recent self-harm in the past year – alone or homeless, cutting used as a method of harm, treatment for at psychiatric disorder; **SIS** = Beck's suicide intent scale; **SSI-C/SSI-W** = Scale for suicide ideation – Current/Worst

Resultat

Instrument använda i psykiatrisk specialistvård

BHS – Beck's Hopelessness Scale

Beskrivning av ingående studier

Fem studier genomförda i Sverige, USA och Tyskland mellan åren 1990 och 2012 [6–10] ingick i analysen. Studiepopulationen bestod av totalt 6 346 vuxna personer. Medelåldern varierade i studierna mellan 29 och 47 år och könsfördelningen kvinnor var mellan 57 och 66 procent. I de aktuella studierna rekryterades populationen från olika specialist-mottagningar för depression och ångestsjukdomar [6,7,9], från en slutenvårdsavdelning för patienter med depression [8] samt från en slut-envårdsavdelning för patienter nyinsjuknade i psykossjukdom [10]. Uppföljningstiden varierade från 1 månad upp till 19 år. I studierna hade majoriteten depression och ångestsjukdom. Övriga diagnoser spände över krisreaktion, beteendestörning, missbruk samt schizofreni/psykos.

Bedömning av sensitivitet och specificitet

Fyra av studierna kunde vägas samman [6–9]. Dessa visade för suicid en sensitivitet på 89 procent och specificitet på 42 procent (Figur 4.2). Studien av Klonsky och medarbetare bedömdes skilja sig från övriga studier på grund av patientpopulationen och ingick därför inte i den sammanvägda analysen [10]. Här var sensitiviteten 33 procent och specificiteten 88 procent för personer med psykossjukdom.

Studie, år, referens	Sensitivitet (95% KI)	Specificitet (95% KI)
Beck 1990 [6]	0,94 (0,71; 1,00)	0,41 (0,39; 0,43)
Beck 1999 [7]	0,90 (0,73; 0,98)	0,42 (0,40; 0,44)
Keller 1993 [8]	1,00 (0,16; 1,00)	0,27 (0,16; 0,40)
Nimeus 1997 [9]	0,77 (0,46; 0,95)	0,42 (0,35; 0,49)

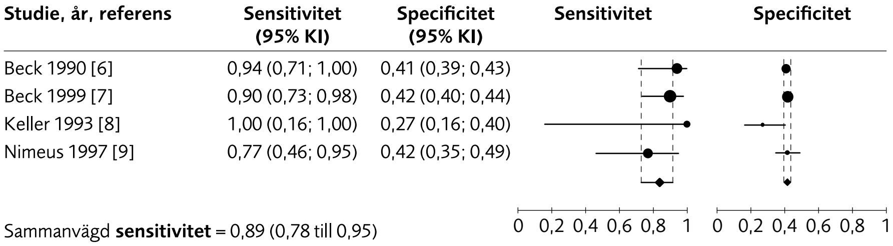

Sammanvägd **sensitivitet** = 0,89 (0,78 till 0,95)
Sammanvägd **specificitet** = 0,42 (0,40 till 0,43)

Figur 4.2 Sensitiviteten och specificiteten för skattningsskalan BHS.

Bedömning av vetenskapligt stöd.

Vi fann inga faktorer som motiverade avdrag i GRADE när det gällde de fyra sammanvägda studierna (Tabell 4.2) [6–9]. Det vetenskapliga underlaget blev därför starkt för att sensitiviteten och specificiteten var 89 procent (95% KI, 78; 95) respektive 42 procent (95% KI, 40; 42).

Vid bedömning av suicidrisk hos patienter med psykossjukdom bedömdes det sammanlagda vetenskapliga underlaget för sensitivitet och specificitet som otillräckligt för skattningsinstrumentet BHS [10]. Vi gjorde avdrag för precision på grund av mycket brett konfidensintervall för sensitiviteten. Det blev även avdrag för stort bortfall (studiekvalitet) samt bristande överförbarhet då det endast var en studie. Otillräckligt underlag betyder att resultaten inte bedöms vara tillförlitliga.

Tabell 4.2 Sammanfattande tabell för instrumentet BHS.

Patient population		Antal patienter (studier)	Antal händelser	Poolat estimat (95% KI)	Veten-skapligt underlag	Avdrag
Depression och ångest-sjukdomar [6–9]	Sensitivitet	5 932 (4)	62	89 (78; 95)	Starkt ⊕⊕⊕⊕	
	Specificitet	5 932 (4)	62	42 (40; 43)	Starkt ⊕⊕⊕⊕	
	Sammanlagd evidens				Starkt ⊕⊕⊕⊕	
Nyinsjuknade i psykos-sjukdom [10]	Sensitivitet	414 (1)	6	33 (4; 78)	Otillräckligt ⊕○○○	–1 Studie-kvalitet –1 Över-förbarhet –2 Preci-sion
	Specificitet	414 (1)	6	88 (84; 91)	Begränsat ⊕⊕○○	–1 Studie-kvalitet –1 Över-förbarhet
	Sammanlagd evidens				Otillräckligt ⊕○○○	

BHS = Beck's hoplessness scale; **KI** = Konfidensintervall

Konsekvens av våra resultat

För att ett instrument enligt våra krav ska anses tillförlitligt, måste sensitiviteten vara mer än 80 procent och specificiteten mer än 50 procent. Konsekvensen blir därför att det finns starkt vetenskapligt stöd för att BHS som screeninginstrument inte är användbart för denna population inom specialistvård, då specificiteten är för låg.

När det gäller patienter med psykossjukdom var det vetenskapliga underlaget otillräckligt, vilket innebär att vi inte kan bedöma instrumentets tillförlitlighet för denna patientgrupp.

SIS – Beck's Suicide Intent Scale

Beskrivning av ingående studier

Två studier från 1990-talet, en från Sverige (555 patienter) [13] och en från England (2 719 patienter) [12] ingick. Deltagarna i den engelska studien bestod av patienter (15 år eller äldre) som sökt akutvård på sjukhus efter en episod av självskadehandling såsom alkohol-, tablettförgiftning eller annan självförvållad skada [12].

Populationen i den svenska studien bestod till största delen av vuxna (medelålder 39 år), men åldersintervallet var 15–92 år [13]. Tjugosex patienter (73 % män) hade gjort allvarliga suicidförsök, och 246 patienter (32 % män) hade gjort upprepade suicidförsök. Patienterna bedömdes kort tid efter suicidförsök, oftast inneliggande på medicinsk intensivvårdsklinik.

Bedömning av sensitivitet och specificitet

Då studierna hade olika tröskelvärden, 19 [13] respektive 10 [12], kan studierna inte vägas samman. Studien av Niméus och medarbetare, med tröskelvärde på 19, visade en sensitivitet på 59 procent och specificitet på 77 procent [9]. Med ett tröskelvärde på 10 var sensitiviteten 76 procent och specificiteten 49 procent [12].

Bedömning av vetenskapligt stöd

I studien av Harriss och medarbetare bedömdes det sammanlagda vetenskapliga underlaget för sensitiviteten och specificiteten som begränsat för personer med självskadehandlingar inom akut specialistvård [12]. Avdrag gjordes för överförbarhet då underlaget endast utgjordes av en studie samt för precision på grund av brett konfidensintervall för sensitiviteten (Tabell 4.3).

I studien av Niméus och medarbetare bedömdes det sammanlagda vetenskapliga underlaget för sensitivitet och specificitet som otillräckligt [13]. Två avdrag gjordes för precision på grund av mycket brett konfidensintervall samt på grund av överförbarhet då underlaget endast utgjordes av en studie.

Tabell 4.3 Sammanfattande tabell för instrumentet SIS.

Population	Tröskelvärde		Antal patienter (studier)	Antal händelser	Estimat (95% KI)	Vetenskapligt underlag	Avdrag
Självskada [12]	10	Sensitivitet	2 719 (1)	54	76 (62; 87)	Begränsat ⊕⊕○○	–1 Överförbarhet –1 Precision
		Specificitet	2 719 (1)	54	49 (47; 51)	Måttligt starkt ⊕⊕⊕○	–1 Överförbarhet
		Sammanlagd evidens				Begränsat ⊕⊕○○	
Suicidförsök [13]	19	Sensitivitet	555 (1)	22	59 (36; 79)	Otillräckligt ⊕○○○	–1 Överförbarhet –2 Precision
		Specificitet	555 (1)	22	77 (74; 81)	Måttligt starkt ⊕⊕⊕○	–1 Överförbarhet
		Sammanlagd evidens				Otillräckligt ⊕○○○	

BHS = Beck's hoplessness scale; **KI** = Konfidensintervall

Konsekvens av resultat

För patienter som gjort suicidförsök var det vetenskapliga underlaget otillräckligt, vilket gör att vi inte kan bedöma instrumentets tillförlitlighet för denna grupp.

För personer som utfört självskadehandling var det vetenskapliga underlaget begränsat, vilket innebär att det finns en stor osäkerhet kring instruments tillförlitlighet för att förutsäga nya suicidförsök. Fler studier behövs för att få kunskap om instruments tillförlitlighet.

SSI-C och SSI-W

Beskrivning av ingående studier

I en studie med 3 701 patienter (57 % kvinnor), rekryterades deltagare från ett center för kognitiv psykoterapi i USA [7]. Deltagarna var övervägande öppenvårdspatienter, där 65 procent hade tidigare sjukhistoria med suicidförsök. Studien genomfördes åren 1975–1994. I början av studietiden användes DSM-III för diagnossättning men efter 1980 övergick man till DSM-III-R. Alla diagnoser översattes senare till DSM-IV. Samtliga deltagare bedömdes med instrumenten SSI-C och SSI-W. Trettio personer avled till följd av suicid under uppföljningstiden som var 15 år. Ett observandum är den varierande tiden till utfall.

Bedömning av sensitivitet och specificitet

Studien visar en sammanvägd sensitivitet på 53 procent och specificitet på 83 procent för SSI-C och en sensitivitet på 80 procent och specificitet på 78 procent för SSI-W.

Bedömning av vetenskapligt stöd

Det sammanlagda vetenskapliga underlaget för sensitivitet och specificitet för SSI-C bedömdes som begränsat. Vi gjorde två poängs avdrag för precision för sensitiviteten då konfidensintervallet var mycket brett (Tabell 4.4).

Det sammanlagda vetenskapliga underlaget för sensitiviteten och specificiteten för SSI-W blev måttligt starkt. Ett poängs avdrag gjordes för brett konfidensintervall för sensitiviteten (Tabell 4.5).

Tabell 4.4 Sammanfattande tabell för SSI-C för öppenvårdspatienter, tröskelvärde >2 [7].

	Antal patienter (studier)	Antal händelser	Estimat (95% KI)	Vetenskapligt underlag	Avdrag
Sensitivitet	3 701 (1)	30	53 (34; 72)	Begränsat ⊕⊕○○	–2 Precision
Specificitet	3 701 (1)	30	83 (82; 84)	Starkt ⊕⊕⊕⊕	
Sammanlagd evidens				Begränsat ⊕⊕○○	

KI = Konfidensintervall; **SSI-C** = Scale for suicide ideation – Current

Tabell 4.5 Sammanfattande tabell för SSI-W, för öppenvårdspatienter, tröskelvärde ≥16.

	Antal patienter (studier)	Antal händelser	Estimat (95% KI)	Vetenskapligt underlag	Avdrag
Sensitivitet	3 701	30	0,80 (0,61; 0,92)	Måttligt starkt ⊕⊕⊕○	–1 Precision
Specificitet	3 701	30	0,78 (0,77; 0,79)	Starkt ⊕⊕⊕⊕	
Sammanlagd evidens				Måttligt starkt ⊕⊕⊕○	

KI = Konfidensintervall; **SSI-W** = Scale for suicide ideation – Worst

Konsekvens av resultat

SSI-C har för låg sensitivitet för att den ska anses användbar. För SSI-W har sensitiviteten för stort konfidensintervall, varav den undre gränsen är 61 procent. Detta gör att instrumentet inte kan anses vara användbart.

Instrument använt i akutmedicinsk vård för triagering till psykiatrisk bedömning

ReACT

Beskrivning av ingående studier

Vi identifierade en studie som omfattade 29 571 tillfällen av självskada gjorda av 18 680 individer [14]. Den var genomförd vid fem akutmottagningar på sjukhus i Storbritannien under 2003–2007. Uppföljningstiden var minst sex månader. Under uppföljningstiden gjorde männen 2 984 (25 %) nya självskadehandlingar och kvinnorna 5 125 (29 %). I samma studie gjordes separata beräkningar för två olika geografiska områden (benämnd 1 och 2 i Figur 4.3) vilket resulterar i två kalkyler av sensitivitet och specificitet.

Bedömning av sensitivitet och specificitet

Studien visade en sammanvägd sensitivitet på 90 procent med ett konfidensintervall mellan 82 och 95 procent och specificitet på 17 procent med ett konfidensintervall mellan 17 och 18 procent (Figur 4.3).

Studie, år, referens	Sensitivitet (95% KI)	Specificitet (95% KI)
Steeg et al 2012 [14]	0,91 (0,81; 0,97)	0,15 (0,15; 0,16)
Steeg et al 2012 [14]	0,88 (0,70; 0,98)	0,24 (0,23; 0,25)

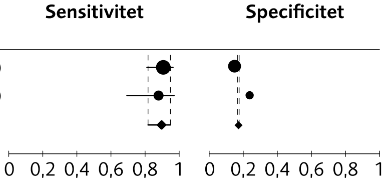

Sammanvägd **sensitivitet** = 0,90 (0,82 till 0,95)
Sammanvägd **specificitet** = 0,17 (0,17 till 0,18)

Figur 4.3 Sensitivitet och specificitet för skattningsskalan ReACT.

Bedömning av vetenskapligt stöd

ReACT är baserat på statistiska riskfaktorer. Det finns inget tröskelvärde angivet. En eller fler av följande riskfaktorer ger ökad risk: tidigare självskada (det senaste året) (Re), att vara ensamstående eller bostadslös (A), skära sig som metod för självskada (C), pågående behandling av psykiatrisk sjukdom (T). Det sammanlagda vetenskapliga underlaget för sensitivitet och specificitet blev måttlig starkt (Tabell 4.6). Avdrag gjordes för bristande samstämmighet avseende specificitet.

Tabell 4.6 Sammanfattande tabell för instrumentet ReACT.

**Patient-population**		**Antal patienter (studier)**	**Antal händelser**	**Estimat (95% KI)**	**Veten-skapligt underlag**	**Avdrag**
Personer med självskade-handling	Sensitivitet	18 680 (1)	92	90 (82; 95)	Starkt ⊕⊕⊕⊕	
	Specificitet	18 680 (1)	92	17 (17; 18)	Måttligt starkt ⊕⊕⊕○	–1 Sam-stämmighet
	Sammanlagd evidens				Måttligt starkt ⊕⊕⊕○	

KI = Konfidensintervall; **ReACT** = Recent self-harm in the past year – alone or homeless, cutting used as a method of harm, treatment for at psychiatric disorder

Konsekvens av våra resultat

Instrumentet hade en låg specificitet (17–18 %) och uppfyllde alltså inte det uppställda kravet på en specificitet mer än 50 procent. Det positiva prediktiva värdet var också mycket lågt (<1 %). Konsekvensen blir därför att ReACT inte är tillämpbart för prediktion av suicid för personer med självskadehandling.

Instrument använt för depressionsskattning i primärvård

PHQ-9 – Patient Health Questionnaire-9

Beskrivning av ingående studier

Denna studie bygger på ett mycket stort patientmaterial från USA med cirka 84 400 patienter, vilken genomfördes i ett integrerat sjukvårdssystem med primärvård/psykiatrisk öppenvård under perioden 2007–2011 [5]. Populationen bestod av ungdomar och vuxna som behandlades för depression.

Bedömning av sensitivitet och specificitet

Studien visade för suicid en sensitivitet på 80 procent och specificitet på 70 procent för personer med depression.

Bedömning av vetenskapligt stöd

Det sammanlagda vetenskapliga underlaget för sensitivitet och specificitet blev begränsat (Tabell 4.7). Trots att det endast fanns en studie gjorde vi inget avdrag då antalet deltagare var mycket stort. Avdrag gjordes för precision på grund av brett konfidensintervall, och för oklar resultatredovisning (studiekvalitet). Studien hade inte gjort subgruppsanalyser. Begränsat vetenskapligt underlag betyder att resultaten är osäkra. Det är möjligt att ny forskning kan visa andra resultat.

Tabell 4.7 Sammanfattande tabell för instrumentet PHQ-9 för patienter med depression inom primärvården. Tröskelvärde: flera dagar under de sista 2 veckorna.

	**Antal patienter (studier)**	**Antal händelser**	**Estimat (95% KI)**	**Vetenskapligt underlag**	**Avdrag**
Sensitivitet	84 418 (1)	46	80 (66; 91)	Begränsat ⊕⊕○○	–1 Studiekvalitet<br>–1 Precision
Specificitet	84 418 (1)	46	70 (70; 71)	Måttligt starkt ⊕⊕⊕○	–1 Studiekvalitet
Sammanlagd evidens				Begränsat ⊕⊕○○	

PHQ-9 = Patient health questionnaire

Konsekvens av våra resultat

Då det nedre konfidensintervallet ligger under våra krav för att anses tillförlitligt (dvs sensitivitet >80 %) kan vi inte avgöra om instrumentet är användbart för att bedöma suicidrisk i klinisk praxis.

5. Resultat av granskningen: suicidförsök

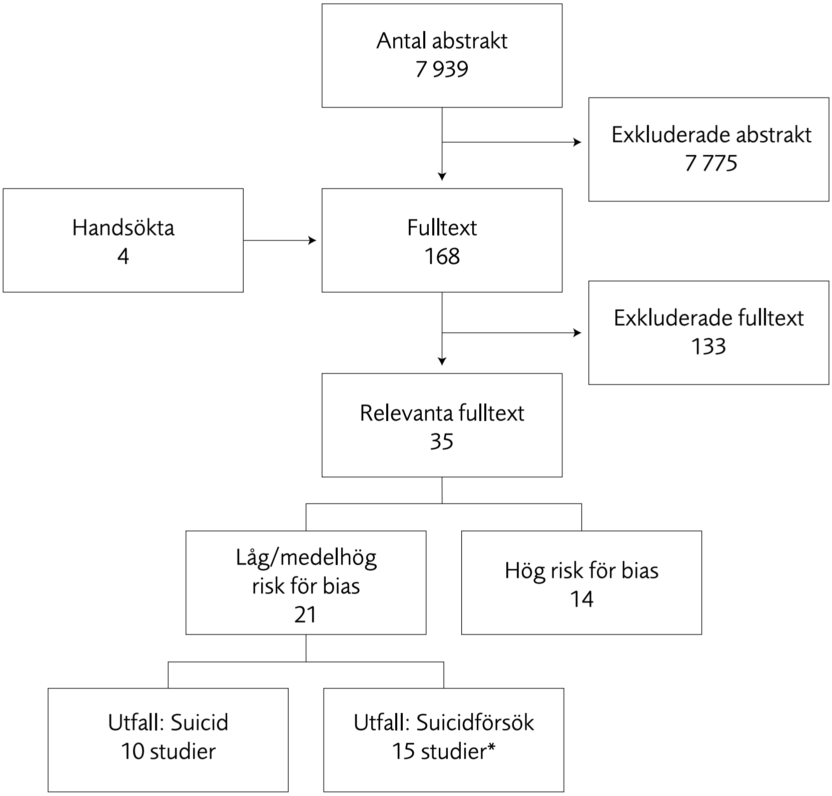

* Fyra studier hade både suicid och suicidförsök som utfall.

Figur 5.1 Flödesschema över litteratursökningen. Med handsökta menas studier som tillkommit utanför litteratursökning.

Av 168 artiklar som beställdes i fulltext uppfyllde sammanlagt 35 artiklar våra urvalskriterier medan 133 (se Figur 5.1) artiklar exkluderades (Bilaga 2). Av de 35 relevanta studierna hade 14 hög risk för bias, och ingår därmed inte i analysen (Bilaga 4). Det var 15 av 21 studier som hade utfallsmåttet suicidförsök och som ingår i detta kapitel.

Sammanfattning av resultaten

Resultaten är uppdelade på specialistpsykiatri, akutmedicinsk vård och primärvård. För samtliga instrument studerade inom psykiatrisk vård är resultatet otillräckligt (⊕○○○) eller begränsat (⊕⊕○○). För skalor inom akutmedicinsk vård har MSHR, ReACT, SoS-4, starkt eller måttligt starkt stöd för att de inte är tillförlitliga för prediktion då de inte uppfyllde kraven på sensitivitet >80 procent samt specificitet >50 procent (Tabell 5.1).

För primärvård identifierade vi endast en studie [5]. Den utvärderade den fråga om döds- och suicidtankar som ingår i PHQ-9 (Tabell 5.1). Granskningen visade att det finns ett måttligt starkt stöd (⊕⊕⊕○) för att patienter som bejakar denna fråga har en ökad risk för suicidförsök under en uppföljningstid på ett år.

Tabell 5.1 Sammanfattande resultattabell över identifierade skattningsskalor för psykiatrisk specialistvård, akutmedicinsk vård och primärvård.

Skala	Population	Sensitivitet (95% KI)	Specificitet (95% KI)	Evidensstyrka
**Psykiatrisk specialistvård**				
BHS [8,15]	Suicidförsök	60 (26; 88)	91 (80; 97)	Otillräcklig ⊕○○○
	Depression	80 (44; 97)	27 (16; 42)	Otillräcklig ⊕○○○
C-SSRS* [11]	Suicidförsök, vuxna	100	96	Otillräcklig ⊕○○○
ERRS [16]	Självskada/suicidförsök	26 (20; 33)	84 (82; 86)	Måttligt stark ⊕⊕⊕○
IAT* [17]	Självskada/suicidförsök	43 (26; 63)	79 (68; 87)	Otillräcklig ⊕○○○
MINI ≥10 [18]	Akut psykiatrisk vårdavdelning	61 (47; 73)	75 (69; 80)	Begränsad ⊕⊕○○
SAD PERSONS Scale [19,20]	Självskada/psykiatriska akutmottagningar	15 (8; 24)	97 (96; 98)	Stark ⊕⊕⊕⊕

Tabellen fortsätter på nästa sida

Tabell 5.1 fortsättning

Skala	Population	Sensitivitet (95% KI)	Specificitet (95% KI)	Evidensstyrka
Modified SAD PERSONS Scale [19]	Psykiatriska akutmottagningar	29 (19; 40)	89 (88; 90)	Begränsad ⊕⊕○○
SPS [21]	Barn/ungdomar utsatta för övergrepp/ med beteendestörningar	48 (29; 67)	80 (77; 83)	Begränsad ⊕⊕○○
SUAS* [22]	Vuxna, suicid/ suicidförsök	20 (14; 47)	93(83; 98)	Otillräcklig ⊕○○○
**Akutmedicinsk vård**				
MSHR [14,23–26]	Självskada/suicidförsök/ suicid	97	20 (20; 21)	Stark ⊕⊕⊕⊕
ReACT [14]	Självskada/suicidförsök	94 (93; 94)	24 (23; 25)	Måttligt stark ⊕⊕⊕○
SoS-4 [25,26]	Självskada/suicidförsök	90 (86; 93)	17 (15; 19)	Stark ⊕⊕⊕⊕
**Primärvård**				
PHQ-9 [5]	Depression, 13 år eller äldre	78 (74; 81)	70 (70; 71)	Måttligt stark ⊕⊕⊕○

* Endast en studie och antalet deltagare var lägre än 200. Detta gör att det vetenskapliga underlaget automatiskt blir otillräckligt (⊕○○○).

IAT = Implicit association test; **MINI** = International neuropsychiatric interview; **MSHR** = Manchester self harm rule; **PHQ-9** = Patient health questionnaire-9; **ReACT** = Recent self-harm in the past year – alone or homeless, cutting used as a method of harm, treatment for at psychiatric disorder; **SAD PERSONS scale** = SAD (sex, age, depression) PERSONS previous attempt, ethanol abuse, rational thinking loss, social support lacking, organised plan, no spouse, sickness); **SoS-4** = Södersjukhuset self-harm rule; **SPS** = Suicide probability scale; **SUAS** = Suicide assessment scale

Resultat

Instrument använda i psykiatrisk specialistvård

BHS – Beck's Hopelessness Scale

Beskrivning av ingående studier

Två kohortstudier med totalt 299 vuxna patienter genomförda i Storbritannien och Tyskland åren 1986–2010 ingick [8,15]. Uppföljningstiden var ett år. Patienterna rekryterades från akutmottagningar [15] samt från en depressionsavdelning [8] och medelåldern varierade mellan 34 och 47 år. Inkluderade diagnoser hos studiedeltagarna var bland annat depression, bipolär sjukdom, dystymi eller krisreaktion.

Tabell 5.2 Resultattabell för instrumentet BHS.

Population	Tröskelvärde	Sensitivitet (95% KI)	Specificitet (95% KI)
Personer med depression och bipolär sjukdom [8]	≥9	80 (44; 97)	26 (16; 42)
Suicidförsök [15]	≥2	58 (37; 78)	87 (73; 96)

KI = Konfidensintervall

Bedömning av sensitivitet och specificitet

Studierna kunde inte sammanvägas på grund av skilda tröskelvärden. Resultaten för sensitivitet och specificitet för respektive studie redovisas i Tabell 5.2.

Bedömning av vetenskapligt stöd

Det sammanlagda vetenskapliga underlaget för sensitivitet och specificitet var otillräckligt då det endast fanns en studie för varje använt tröskelvärde och då studiekohorterna var små (Tabell 5.3).

Tabell 5.3 Sammanfattande tabell för BHS.

Population	Tröskelvärde		Antal patienter (studier)	Antal händelser	Estimat (95% KI)	Vetenskapligt underlag	Avdrag
Personer med depression och bipolär sjukdom [8]	≥9	Sensitivitet	61 (1)	8	80 (44; 97)	Otillräckligt ⊕○○○	1 studie
		Specificitet	61 (1)	8	26 (16; 42)	Otillräckligt ⊕○○○	1 studie
		Sammanlagd evidens				Otillräckligt ⊕○○○	
Suicidförsök [15]	>2	Sensitivitet	66 (1)	25	58 (37; 78)	Otillräckligt ⊕○○○	1 studie
		Specificitet	66 (1)	25	87 (73; 96)	Otillräckligt ⊕○○○	1 studie
		Sammanlagd evidens				Otillräckligt ⊕○○○	

BHS = Beck's hoplessness scale

Konsekvens

Det går inte att bedöma instrumentens tillförlitlighet.

ERRS – Edinburgh Risk of Repetition Scale

Beskrivning av ingående studier

ERRS är utvärderat i en prospektiv studie från 1990-talet [16]. Studien genomfördes på ett sjukhus för patienter med självskadebeteende under 1990-talet. Deltagarna, som i genomsnitt var 33 år, kom till sjukhuset efter självskada genom förgiftning. Sextiotvå procent av deltagarna var kvinnor.

Bedömning av sensitivitet och specificitet

Studien visade en sensitivitet på 26 procent med ett konfidensintervall mellan 20 till 33 procent och specificitet på 84 procent med ett konfidensintervall mellan 82 till 86 procent, för patienter med senare upprepat självskadebeteende.

Bedömning av vetenskapligt stöd

Det sammanlagda vetenskapliga underlaget för sensitiviteten och specificiteten blev måttligt starkt. Vi gjorde avdrag för överförbarhet då underlaget endast utgjordes av en studie (Tabell 5.4).

Tabell 5.4 Sammanfattande tabell för instrumentet ERRS. Tröskelvärde: män≥8, kvinnor≥6. Population: självskadebeteende.

	**Antal patienter (studier)**	**Antal händelser**	**Estimat (95% KI)**	**Vetenskapligt underlag**	**Avdrag**
Sensitivitet	1 317 (1)	180	26 (20; 33)	Måttligt starkt ⊕⊕⊕○	–1 Överförbarhet
Specificitet	1 317 (1)	180	84 (82; 86)	Måttligt starkt ⊕⊕⊕○	–1 Överförbarhet
Sammanlagd evidens				Måttligt starkt ⊕⊕⊕○	

ERRS = Edinburgh risk of repetition scale; **KI** = Konfidensintervall

Konsekvens

Instrumentet ERRS är prövat vid akutbedömning av individer som gjort suicidförsök. Den enda av ingående faktorer som hade någon prediktiv betydelse för upprepning av suicidhandling var om personen vid en föregående episod också förekommit självskada. Instrumentet har ingen klinisk relevans då sensitiviteten är för låg (20–33 %) och det vetenskapliga underlaget är måttligt starkt, vilket innebär att det inte är troligt att nya studier kommer att ändra resultatet.

MINI – International Neuropsychiatric Interview

Beskrivning av ingående studier

En studie från Norge uppfyllde studiekriterierna. I studien ingick 489 personer från en akut psykiatrisk vårdavdelning och utfördes under åren 2006–2008 [18]. Med det diagnostiska instrument MINI:s Suicidal Scale undersöktes om det gick att förutsäga suicidförsök inom 12 månader. Sammanlagt gjorde 37 procent en självskada med eller utan suicidal intention under uppföljningen.

Bedömning av sensitivitet och specificitet

Resultatet visade för suicidförsök en sensitivitet på 61 procent med ett konfidensintervall mellan 47 och 73 procent och specificitet på 75 procent med ett konfidensintervall mellan 69 till 80 procent.

Bedömning av vetenskapligt stöd

Det sammanlagda vetenskapliga underlaget för sensitiviteten och specificiteten var begränsat (Tabell 5.5). Vi gjorde avdrag för överförbarhet då det bara fanns en mindre studie. Avdrag gjordes också för bristande precision när det gällde sensitivitet på grund av ett brett konfidensintervall.

Tabell 5.5 Sammanfattande tabell för MINI Suicidal Scale, tröskelvärde ≥10/33 [18].

	**Antal patienter (studier)**	**Antal händelser**	**Estimat (95% KI)**	**Vetenskapligt underlag**	**Avdrag**
Sensitivitet	307 (1)	64	61 (47; 73)	Begränsat ⊕⊕○○	–1 Överförbarhet<br>–1 Precision
Specificitet	307 (1)	64	75 (69; 80)	Måttligt starkt ⊕⊕⊕○	–1 Överförbarhet
Sammanlagd evidens				Begränsat ⊕⊕○○	

KI = Konfidensintervall; **MINI** = International neuropsychiatric interview

Konsekvens

Sensitiviteten uppfyller inte de kriterier vi angav för att tillämpas i psykiatrisk akutvård. MINI:s Suicidal Scale har i denna studie inte visats kunna identifiera en tillräcklig andel av de patienter som senare gör ett suicidförsök. Eftersom underlaget är begränsat behövs det flera studier för att man ska kunna värdera delskalan. I nuläget kan vi inte uttala oss om värdet av MINI:s Suicidal Scale.

SAD – SAD PERSONS Scale

Beskrivning av ingående studier

Skalan består utav 10 faktorer som sedan länge ansetts vara riskfaktorer för suicid. Skalan har studerats i två kohortstudier med sex månaders uppföljning från Kanada och Storbritannien [19,20]. I den kanadensiska studien rekryterades 2 846 patienter, konsekutiva fall som hade remitterats till psykiatriska akutmottagningar vid två allmänna sjukhus [19]. Inom sex månader gjorde 80 individer en självskada. Den andra avsevärt mindre studien gjordes i Storbritannien, och patienterna rekryterades av ett speciellt "self-harm"-team på ett allmänt sjukhus, och inkluderade 126 konsekutiva patienter, 57 procent kvinnor [20]. Man inkluderade patienter som gjort en självskadehandling oavsett om de haft ett suicidalt syfte eller inte. Åttioen procent av populationen hade tagit en överdos (förgiftning), 11 procent hade skurit sig och 8 procent hade annan typ av självskada.

Bedömning av sensitivitet och specificitet

Studierna visade en sammanvägd sensitivitet på 15 procent och specificitet på 97 procent (Figur 5.2), vid tröskelvärdet >7.

Studie, år, referens	Sensitivitet (95% KI)	Specificitet (95% KI)
Bolton et al 2012 [19]	0,20 (0,10; 0,33)	0,97 (0,96; 0,98)
Saunders et al 2013 [20]	0,06 (0,01; 0,21)	0,97 (0,91; 0,99)

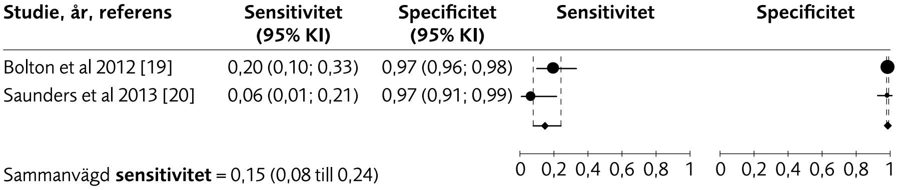

Sammanvägd **sensitivitet** = 0,15 (0,08 till 0,24)
Sammanvägd **specificitet** = 0,97 (0,96 till 0,98)

Figur 5.2 Sensitivitet och specificitet för SAD PERSONS Scale.

Bedömning av vetenskapligt stöd

Det sammanlagda vetenskapliga underlaget för sensitiviteten och specificiteten bedömdes som starkt då inga avdrag gjordes (Tabell 5.6). Detta innebär att vi är mycket säkra på att sensitiviteten och specificiteten ligger runt 18 procent respektive 97 procent, det vill säga att det är osannolikt att nya studier förändrar utfallet, tröskelvärde >7.

Tabell 5.6 Sammanfattande tabell för SAD PERSONS Scale, tröskelvärde >7.

	**Antal patienter (studier)**	**Antal händelser**	**Poolat estimat (95% KI)**	**Vetenskapligt underlag**	**Avdrag**
Sensitivitet	2 972 (2)*	471	15 (8; 24)	Starkt ⊕⊕⊕⊕	
Specificitet	2 972 (2)*	471	97 (96; 98)	Starkt ⊕⊕⊕⊕	
Sammanlagd evidens				Starkt ⊕⊕⊕⊕	

* [19,20].

KI = Konfidensintervall; **SAD PERSONS scale** = SAD (sex, age, depression) PERSONS previous attempt, ethanol abuse, rational thinking loss, social support lacking, organised plan, no spouse, sickness)

Konsekvens

SAD PERSONS Scale är inte ett kliniskt användbart skattningsinstrument inom specialistvården då det har alldeles för låg sensitivitet. Detta innebär att det blir för många falskt negativa utfall, det vill säga att personer med hög risk för att göra suicidförsök inte upptäcks.

MOD SAD – Modified SAD PERSONS Scale

Beskrivning av ingående studier

Fyra frågor vägdes dubbelt i denna modifierade SAD PERSONS Scale, där även frågan för sjukdom byttes ut mot en ny fråga; rapporterad suicidavsikt. En kohortstudie från Kanada gällde 2 713 konsekutiva patienter som rekryterades mellan åren 1999 och 2010, med en uppföljningstid på 6 månader [19]. Patienterna rekryterades från två psykiatriska akutmottagningar. Modified SAD PERSONS Scale skattades av ST-läkare under handledning av specialister. Suicidförsök definierades som en självskadehandling med åtminstone någon önskan om att dö. Suicidförsök observerades hos 76 individer under sex månaders uppföljning.

Bedömning av sensitivitet och specificitet

Studierna visade för de akutpsykiatriska patienterna en sensitivitet på 29 procent och en specificitet på 89 procent för suicidförsök inom 6 månader.

Bedömning av vetenskapligt stöd

Det vetenskapliga underlaget sammanlagt för sensitivitet och specificitet bedömdes som begränsat (Tabell 5.7). Då underlaget endast utgjordes av en studie innebär detta en osäkerhet och därför gjordes ett avdrag i överförbarhet för både sensitivitet och specificitet. Vi gjorde även avdrag för precision på grund av brett konfidensintervall för sensitiviteten. Begränsat vetenskapligt underlag betyder att resultaten är osäkra. Det är troligt att ny forskning kan visa andra resultat.

Tabell 5.7 Sammanfattande tabell för instrumentet Modified SAD PERSONS Scale, tröskelvärde ≥16.

	**Antal patienter (studier)**	**Antal händelser**	**Estimat (95% KI)**	**Veten-skapligt underlag**	**Avdrag**
Sensitivitet	2 713 (1)	76	29 (19; 40)	Begränsat ⊕⊕○○	–1 Överförbarhet<br>–1 Precision
Specificitet	2 713 (1)	76	0,89 (88; 90)	Måttligt starkt ⊕⊕⊕○	–1 Överförbarhet
Sammanlagd evidens				Begränsat ⊕⊕○○	

KI = Konfidensintervall; **Modified SAD PERSONS scale** = Modifierad SAD (sex, age, depression) PERSONS previous attempt, ethanol abuse, rational thinking loss, social support lacking, organised plan, no spouse, sickness)

Konsekvens

För att ett instrument ska vara tillförlitligt måste sensitiviteten vara >80 procent. Konsekvensen blir därför att instrumentet Modfied SAD PERSONS Scale, inte är kliniskt användbart då sensitiviteten var så låg som 29 procent.

SPS – Suicide Probability Scale

Beskrivning av ingående studie

Det fanns en studie från USA som uppfyllde studiekriterierna [21]. Populationen bestod av barn och ungdomar (552 pojkar och 277 flickor i åldern 8–18 år) som placerats i särskilda behandlingshem på grund av att de utsatts för övergrepp eller uppvisat beteendestörningar under perioden 1988–1991. Uppföljningstiden varierade från 4 dagar till 42 månader. Tjugonio ungdomar genomförde minst ett suicidförsök under denna period.

Bedömning av sensitivitet och specificitet

Det sammanvägda resultatet visade en sensitivitet på 48 procent och en specificitet på 80 procent.

Bedömning av vetenskapligt stöd

Det vetenskapliga underlaget sammanlagt för sensitivitet och specificitet bedömdes som begränsat (Tabell 5.8). Vi gjorde avdrag för överförbarhet då underlaget endast utgjorde av en mindre studie. Vi gjorde även avdrag för precision på grund av brett konfidensintervall när det gällde sensitivitet.

Tabell 5.8 Sammanfattande tabell för SPS, tröskelvärde ≥78 [21].

	**Antal patienter (studier)**	**Antal händelser**	**Estimat (95% KI)**	**Veten-skapligt underlag**	**Avdrag**
Sensitivitet	834 (1)	29	48 (29; 67)	Begränsat ⊕⊕○○	–1 Överförbarhet<br>–1 Precision
Specificitet	834 (1)	29	80 (77; 83)	Måttligt starkt ⊕⊕⊕○	–1 Överförbarhet
Sammanlagd evidens				Begränsat ⊕⊕○○	

KI = Konfidensintervall; **SPS** = Suicide probability scale

Konsekvens

Då sensitiviteten var långt ifrån vårt förutbestämda krav på >80 procent, innebär detta att SPS sannolikt inte kan anses användbart.

Instrument använda i akutmedicinsk vård för triage till psykiatrisk bedömning

MSHR – Manchester Self Harm Rule

Beskrivning av ingående studier

MSHR är ett kort screeninginstrument för somatisk akutvård som innehåller fyra frågor: om tidigare självskada, tidigare psykiatrisk behandling, om bensodiazepin har använts vid aktuellt försök och om det finns en pågående psykiatrisk kontakt. En av dessa frågor ska kunna bejakas.

Detta instrument har utvärderats i fem studier [14,23–26]. Originalstudien från Storbritannien omfattade 9 086 händelser som följts under sex månader. Vid uppföljning identifierades 1 538 nya självskadetillfällen varav 22 med dödlig utgång [23]. En annan brittisk studie gällde 18 680 personer från fem olika centra, varav 8 109 utförde en ny självskada inom uppföljningstiden som var minst sex månader [14]. I en betydligt mindre studie från Kanada ingick 157 personer över 18 år, varav 34 personer utförde en ny självskada inom 3 månader. Denna studie hade stort bortfall. Instrumentet MSHR har också prövats i två svenska studier. Den första gjordes på två sjukhus i Stockholm och omfattade 325 personer över 18 år, varav 80 gjorde en ny självskada [25]. Den andra gjordes av samma forskarteam på ett av dessa Stockholmssjukhus men omfattade 1 524 personer i samma ålder, varav 309 gjorde en ny självskada (med eller utan fatal utgång) under samma uppföljningstid på sex månader [26]. Totalt omfattade studierna 29 772 individer.

Bedömning av sensitivitet och specificitet

Vi bedömde att studierna var tillräckligt lika för att deras resultat skulle kunna vägas samman. Det sammanvägda resultatet visade en sensitivitet på 97 procent och specificitet på 20 procent (Figur 5.3).

Studie, år, referens	Sensitivitet (95% KI)	Specificitet (95% KI)
Bilén 2013 [26]	0,89 (0,85; 0,92)	0,21 (0,19; 0,24)
Bilén 2013 [25]	0,94 (0,85; 0,98)	0,18 (0,14; 0,24)
Cooper 2006 [23]	0,97 (0,94; 0,98)	0,26 (0,24; 0,29)
Cooper 2006 [23]	0,94 (0,92; 0,95)	0,25 (0,24; 0,26)
Randall 2012 [24]	0,94 (0,80; 0,99)	0,15 (0,09; 0,22)
Steeg 2012 [14]	0,98 (0,98; 0,99)	0,17 (0,16; 0,17)
Steeg 2012 [14]	0,97 (0,96; 0,98)	0,20 (0,18; 0,21)

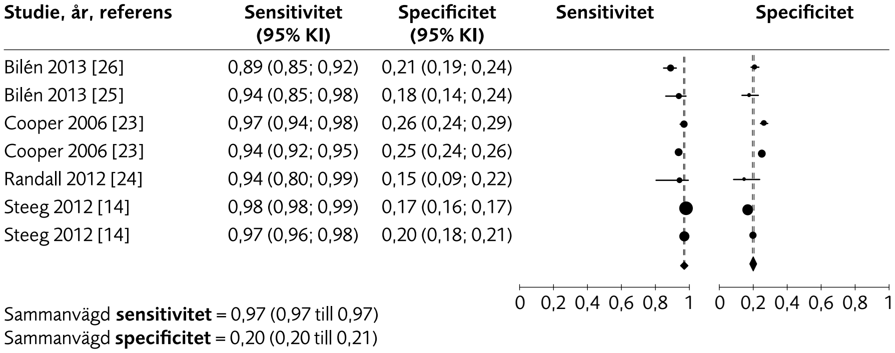

Sammanvägd **sensitivitet** = 0,97 (0,97 till 0,97)
Sammanvägd **specificitet** = 0,20 (0,20 till 0,21)

Figur 5.3 Sensitivitet och specificitet för skattningsinstrumentet MSHR.

Bedömning av vetenskapligt stöd

Det vetenskapliga underlaget sammanlagt för sensitivitet och specificitet bedömdes som starkt då inga avdrag gjordes (Tabell 5.9). Detta innebär att vi kan vara säkra på att sensitiviteten och specificiteten ligger runt 97 procent respektive 20 procent för personer med upprepat självskadebeteende på somatisk akutmottagning. Det är osannolikt att nya studier förändrar utfallet.

Tabell 5.9 Sammanfattande tabell för MSHR. Population: självskadebeteende.

	Antal patienter (studier)	Antal händelser	Poolat estimat (95% KI)	Vetenskapligt underlag	Avdrag
Sensitivitet	29 772 (5)	9 523	97 (97; 97)	Starkt ⊕⊕⊕⊕	
Specificitet	29 772 (5)	9 523	20 (20; 21)	Starkt ⊕⊕⊕⊕	
Sammanlagd evidens				Starkt ⊕⊕⊕⊕	

KI = Konfidensintervall; **MSHR** = Manchester self harm rule

Konsekvens

Instrumentet MSHR är prövat i somatisk akutvård på ett stort antal patienter. Med dess höga sensitivitet skulle MSHR kunna vara av värde för triage till psykiatrisk konsultation, trots sin låga specificitet med många falskt positiva. Om instrumentet används för denna form av screening kan det positiva prediktiva värdet (PPV) på 20–37 procent tala för instrumentets fördel. Alla som gjort suicidförsök bör enligt kliniska riktlinjer bli föremål för psykiatrisk konsultation. Frågan är därför om instrumentet bidrar med ytterligare information för beslutsstöd.

ReACT

Beskrivning av ingående studier

ReACT (Recent self-harm in the past year – Alone or homeless, Cutting used as a method of harm, Treatment for a psychiatric disorder) är en reviderad version av MSHR. Vi identifierade en studie som omfattade 29 571 tillfällen av självskada gjorda av 18 680 individer genomförd vid fem akutmottagningar på sjukhus i Storbritannien under 2003–2007 [14]. Uppföljningen var minst sex månader. Männen gjorde nya självskadehandlingar i 2 984 fall (24,9 %) och av kvinnorna gjorde 5 125 (29 %) nya självskadehandlingar under uppföljningstiden. I samma studie gjordes separata beräkningar för två olika geografiska områden (benämnt som 1 och 2 i Figur 5.4), varför man får två kalkyler för sensitivitet och specificitet.

Bedömning av sensitivitet och specificitet

Studien visade en sammanvägd sensitivitet på 94 procent och specificitet på 24 procent för personer med upprepat självskadebeteende (Figur 5.4).

Studie, år, referens	Sensitivitet (95% KI)	Specificitet (95% KI)
Steeg 2012 [14]	0,95 (0,94; 0,95)	0,21 (0,21; 0,22)
Steeg 2012 [14]	0,90 (0,89; 0,91)	0,34 (0,32; 0,35)

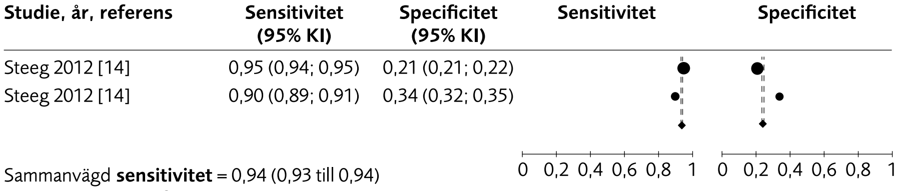

Sammanvägd **sensitivitet** = 0,94 (0,93 till 0,94)
Sammanvägd **specificitet** = 0,24 (0,23 till 0,25)

Figur 5.4 Sensitivitet och specificitet för ReACT.

Bedömning av vetenskapligt stöd

Vi bedömde det vetenskapliga underlaget sammanlagt för sensitivitet och specificitet vara måttligt starkt (Tabell 5.10). Vi gjorde avdrag för samstämmighet då resultaten inte var entydiga avseende specificitet. Måttligt starkt underlag betyder att vi bedömer resultaten som relativt tillförlitliga. Det är inte troligt att ytterligare forskning kan förändra bilden.

Tabell 5.10 Sammanfattande tabell för instrumentet REACT [14].

	**Antal patienter (studier)**	**Antal händelser**	**Estimat (95% KI)**	**Vetenskapligt underlag**	**Avdrag**
Sensitivitet	18 680 (1)	8 110	94 (93; 94)	Starkt ⊕⊕⊕⊕	
Specificitet	18 680 (1)	8 110	24 (23; 25)	Måttligt starkt ⊕⊕⊕○	–1 Samstämmighet
Sammanlagd evidens				Måttligt starkt ⊕⊕⊕○	

KI = Konfidensintervall; **ReACT** = Recent self-harm in the past year – alone or homeless, cutting used as a method of harm, treatment for at psychiatric disorder

Konsekvens

För att ett instrument ska anses tillförlitligt för prediktion, enligt de krav vi uppställt, måste sensitiviteten vara >80 procent och specificiteten >50 procent. Konsekvensen blir därför att ReACT inte är tillämpbart inom för prediktion av självskadebeteende, då specificiteten är för låg. Studierna hade positivt prediktivt värde (PPV) på 30–37 procent. Därför skulle det kunna vara användbart vid triagering inför psykiatrisk bedömning, men med samma reservation som ovan gällande MSHR.

SoS-4 – Södersjukhuset Self-Harm Rule

Beskrivning av ingående studier

Två studier har genomförts i Sverige av samma forskarteam med en modifikation av screeninginstrumentet MSHR. De ingående faktorerna är kön, antidepressiv behandling, tidigare självskada och pågående psykiatrisk kontakt. Vid den första studien rekryterades 325 personer på två sjukhus i Stockholm vilka sökt vård efter självskada. Inom sex månader gjorde 80 personer en ny självskada [25]. Den andra studien som var avsevärt större gjordes på ett av dessa Stockholmssjukhus och omfattade 1 524 personer [26], varav 309 gjorde en ny självskada under samma uppföljningstid på sex månader.

Bedömning av sensitivitet och specificitet

Studierna visade en sammanvägd sensitivitet på 90 procent och specificitet på 17 procent avseende upprepat självskadebeteende (Figur 5.5).

Studie, år, referens	Sensitivitet (95% KI)	Specificitet (95% KI)
Bilén 2013 [26]	0,90 (0,86; 0,93)	0,18 (0,16; 0,20)
Bilén 2013 [25]	0,89 (0,80; 0,95)	0,11 (0,07; 0,16)

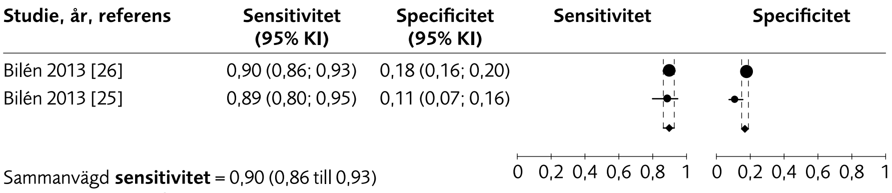

Sammanvägd **sensitivitet** = 0,90 (0,86 till 0,93)
Sammanvägd **specificitet** = 0,17 (0,15 till 0,19)

Figur 5.5 Sensitivitet och specificitet för SoS-4.

Bedömning av vetenskapligt stöd

Det vetenskapliga underlaget sammanlagt för sensitivitet och specificitet bedömdes vara starkt för de prediktionsmått som redovisats ovan (Tabell 5.11).

Tabell 5.11 Sammanfattande tabell för instrumentet SoS-4, tröskelvärde 0,14.

	**Antal patienter (studier)**	**Antal händelser**	**Poolat estimat (95% KI)**	**Vetenskapligt underlag**	**Avdrag**
Sensitivitet	1 849 (2)	389	90 (86; 93)	Starkt ⊕⊕⊕⊕	
Specificitet	1 849 (2)	389	17 (15; 19)	Starkt ⊕⊕⊕⊕	
Sammanlagd evidens				Starkt ⊕⊕⊕⊕	

KI = Konfidensintervall; **SoS-4** = Södersjukhuset self-harm rule

Konsekvens

I linje med resonemangen för MSHR och ReACT är SoS-4 inte tillämpbart för prediktion då specificiteten är för låg. Detta instrument hade positivt prediktivt värde (PPV) på 22–25 procent. Det skulle kunna användas vid triagering inför psykiatrisk bedömning inom somatisk akutvård, men med samma invändningar som för MSHR och ReACT.

Primärvård

PHQ-9 – Patient Health Questionnaire (fråga 9)

Beskrivning av ingående studier

Den diagnostiska skalan för diagnostik och symtomdjup PHQ-9 innehåller en fråga avseende dödstankar eller suicidal intention som studerades i en studie från nordvästra USA. Den omfattade 84 418 patienter som behandlades för depression inom ett integrerat sjukvårdssystem under perioden 2007–2011 [5].

Bedömning av sensitivitet och specificitet

Studien visade för suicidförsök en sensitivitet på 78 procent (95 % KI, 74; 81) och specificitet på 71 procent (95 % KI, 70; 71) för personer med depression.

Bedömning av vetenskapligt stöd

Det sammanlagda vetenskapliga underlaget för sensitivitet och specificitet blev måttligt starkt för angivna värden för sensitivitet och specificitet (Tabell 5.12). Vi gjorde avdrag för oklar resultatredovisning (studiekvalitet).

Sensitiviteten var 78 procent med ett relativt snävt konfidensintervall som inrymde 80 procent, vilket var den gräns som förutbestämts som acceptabel för screening inom primärvård. För screening i primärvård hade vi inga krav på specificitet, men denna var relativt hög, 70 procent. Måttligt starkt underlag betyder att vi bedömer resultaten som relativt tillförlitliga. Det är inte troligt att ytterligare forskning kan förändra bilden.

Tabell 5.12 Sammanfattande tabell för instrumentet PHQ-9 för patienter med depression inom primärvården. Tröskelvärde: flera dagar under de senaste 2 veckorna.

	**Antal patienter (studier)**	**Antal händelser**	**Estimat (95% KI)**	**Vetenskapligt underlag**	**Avdrag**
Sensitivitet	84 418 (1)	709	78 (74; 81)	Måttligt starkt ⊕⊕⊕○	–1 Studiekvalitet
Specificitet	84 418 (1)	709	71 (70; 71)	Måttligt starkt ⊕⊕⊕○	–1 Studiekvalitet
Sammanlagd evidens				Måttligt starkt ⊕⊕⊕○	

PHQ-9 = Patient health questionnaire-9

Konsekvens

Frågan i PHQ-9 för dödstankar eller suicidal intention har en sensitivitet som är något för låg för att instrumentet ska vara tillräckligt tillförlitligt. Detta i kombination med det mycket låga PPV-värdet innebär att instrumentet inte är tillförlitligt.

6. Resultat av granskningen: patientsäkerhet och patientens upplevelse

Blir patientsäkerheten bättre om bedömningsinstrument används?

Inga studier identifierades från den systematiska litteratursökningen som studerade om patientsäkerheten blir bättre om bedömningsinstrument används (Figur 6.1). Med patientsäkerhet avser man möjligheten att inom vården förhindra suicidhandling. I Bilaga 2 redovisas de exkluderade studierna.

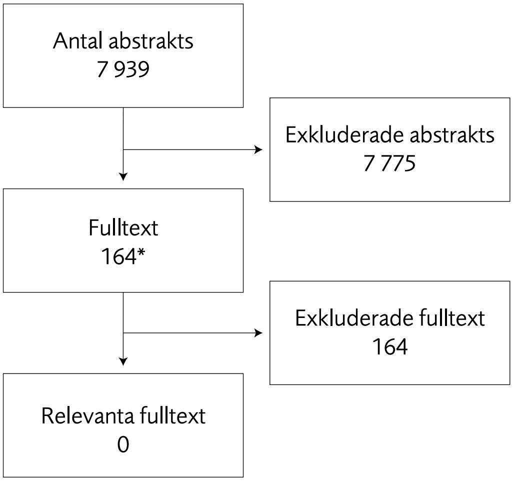

* I dessa 164 studier ingick även de studier som handlade om huruvida bedömningsinstrument på ett tillförlitligt sätt kan förutsäga suicidala handlingar (Kapitel 4 och 5).

Figur 6.1 Flödesschema över litteratursökningen.

Hur upplever patienter användningen av bedömningsinstrument?

Av de 360 artikelsammanfattningar som identifierades i litteratursökningen, beställdes 6 studier i fulltext (Figur 6.2). Endast en av dessa studier uppfyllde våra urvalskriterier medan övriga exkluderades (Bilaga 2) [27].

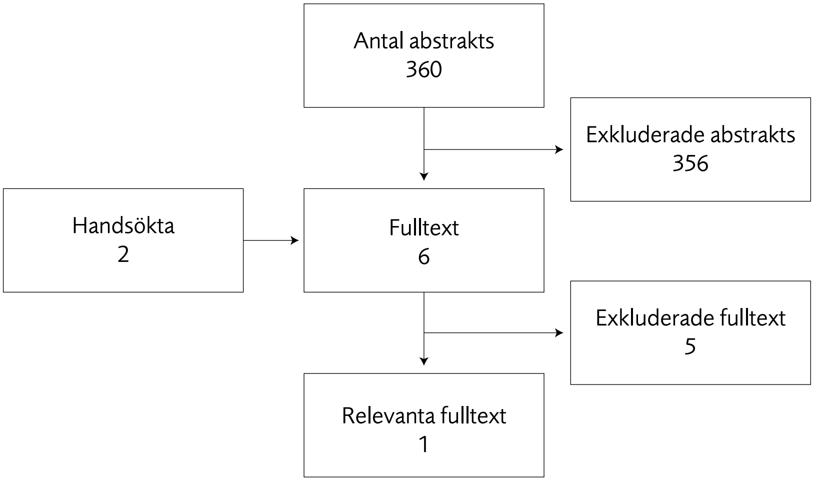

Figur 6.2 Flödesschema över litteratursökningen.

I den aktuella studien ingick 294 ungdomar i åldrarna 13 till 17 år och deras vårdnadshavare (totalt 300 personer) [30]. Studien genomfördes på en somatisk akutmottagning och ett specifikt självskattingsinstrument skapades för studien (The Mental Health Check-up in the Emergency Department–Adolescent/parent Questionnaire). Instrumentet bestod av fem frågor som innefattade psykiatriska sjukdomstillstånd, bland annat suicidrisk, samt attityder till frågor om psykisk hälsa. Studien hade ett bortfall på 11 procent för ungdomar och 9 procent för vårdnadshavare. Studien innehöll 48 procent kvinnor med en medelålder på 15,2 år. Resultaten i studien visade att ungdomar och framför allt kvinnor samt föräldrar var positiva till screening för suicidrisk och att just frågan om suicidrisk uppfattades vara den viktigaste frågan bland både män och kvinnor, tillsammans med frågan om missbruk.

7. Hälsoekonomi

För att underlätta det svåra prioriteringsarbetet i vården är hälsoekonomiska utvärderingar ett hjälpmedel för beslutsfattare. I utvärderingarna jämförs kostnaderna och effekterna för två eller fler alternativ på ett strukturerat sätt för att belysa relationen dem emellan.

När det gäller suicidhandlingar är en vanlig utgångspunkt i samhället att försök att minimera antalet ska göras. Ett sätt att arbeta mot detta mål är att fundera över:

- hur individer med risk för suicid bäst kan identifieras?
- hur vi kan förebygga suicid hos dessa individer?

Den här rapporten granskar enbart instrument för suicidriskbedömning. Dessa instrument används som komplement till den kliniska bedömningen för att identifiera individer med risk. Vi har inte granskat effekterna av olika förebyggande interventioner. Således är de intressanta hälsoekonomiska frågorna i en svensk vårdkontext dessa:

- Kan användandet av bedömningsinstrument som komplement till den kliniska bedömningen förbättra prediktionen av suicidrisken jämfört med enbart den kliniska bedömningen? Och, om så är fallet, står merkostnaden i rimlig proportion till nyttan och effekterna av använda instrumenten?
- Vilket bedömningsinstrument har lägst kostnadseffektkvot?

Ett instruments förmåga att förutsäga suicidrisk har två aspekter: sensitivitet och specificitet. Ju lägre sensitivitet ett instrument har desto fler individer med hög risk för suicid klassificeras fel, vilket leder till att de inte får adekvat förebyggande behandling. Likaså gäller att ju lägre specificitet ett instrument har desto fler individer med låg risk för suicid

klassificeras fel, vilket leder till övervård. Förutom att övervård inte är bra vare sig ur ett medicinskt eller etiskt perspektiv, är det heller inte bra ur ett kostnadsperspektiv. Sammantaget innebär detta att ur en hälsoekonomisk synvinkel förbättras ett instruments kostnadseffektivitet i takt med att instrumentets sensitivitet och/eller specificitet förbättras (i kombination med den kliniska bedömningen), allt annat lika.

Huvudslutsatsen i rapporten är att inga av de identifierade instrumenten har vetenskapligt stöd för tillräcklig tillförlitlighet, vilket delvis beror på att det saknas studier. Det innebär att vi inte vet om det finns bedömningsinstrument som förbättrar diagnostiken utöver den kliniska bedömningen. Det vi vet är att användningen av instrumenten är förknippat med begränsade kostnader i form av liten tidsåtgång för att fylla i och analysera resultaten. Sammantaget innebär rapportens resultat att det i dagsläget är föga meningsfullt att granska eller att göra egna kostnadseffektivitetsanalyser.

8. **Etiska aspekter**

Detta kapitel diskuterar etiska problem som är förknippade med att använda skattningsinstrument vid bedömning av risk för suicid. En utgångspunkt har varit att den systematiska litteraturöversikten visade att det inte fanns några av de identifierade instrumenten som hade vetenskapligt stöd för tillräcklig tillförlitlighet, vilket delvis berodde på att det saknades studier.

Under projekttiden bjöd vi in representanter från brukarorganisationerna inom området liksom representanter från hälso- och sjukvården till en diskussion om etiska aspekter. Mötet ägde rum innan granskningen av litteraturen var klar, vilket medförde att representanterna inte hade en fullständig bild av aktuella etiska aspekter. Analysen av diskussionen bygger på aktörsmodellen [28] och vi utgick från patienter, närstående, bedömare, vårdgivare och samhället som relevanta aktörer.

Användningen av instrument för bedömning av suicidrisk kan förknippas med både positiva och negativa etiska konsekvenser. Tre övergripande teman identifierades:

- påverkan på patient–bedömarrelationen,
- värde för diagnostiken,
- kvalitetssäkring.

Relationen patient – bedömare kan påverkas

Den kliniska suicidriskbedömningen baseras på samtal mellan patient och sjukvårdspersonal. Bedömningen utförs ofta av läkare, men även av andra personalgrupper som sjuksköterskor, kuratorer, psykologer eller mentalskötare. Det är dock läkaren som har det slutliga ansvaret för vilken åtgärd som vidtas efter den kliniska bedömningen. Beslutet fattas i regel av, eller i samråd med, en specialistläkare och en vanlig orsak till inläggning inom psykiatrin är risk för suicid eller annan självskada.

Samtalet bör innehålla värdering av bland annat suicidal intention, tidigare suicidala handlingar och aktuell situation, men utgör i sig också en professionell intervention [29]. Detta förutsätter att samtalsklimatet är präglat av intresse, respekt och empati.

Det är oklart hur användningen av skattningsinstrument som komplement i ett professionellt samtal påverkar relationen mellan patient och bedömare. Å ena sidan skulle instrumenten kunna erbjuda patienter ökad insyn i den diagnostiska processen, liksom ökade möjligheter till aktivt deltagande. Förutsättningen är att de används på ett korrekt sätt, det vill säga att bedömaren har en öppen kommunikation kring hur bedömningen kommer att gå till, vilken typ av frågor som kommer att ställas och vilket eller vilka instrument som kommer att användas. Patienten får därmed möjlighet att ta ställning till förfaringssättet, vilket kan gynna självbestämmandet [30]. Användningen av skattningsinstrument skulle även kunna bidra till att patient och närstående upplever att diagnostiken är noggrann, vilket i sin tur kan öka förtroendet för bedömaren liksom för sjukvården i stort. Skattningsinstrumenten skulle alltså kunna stärka patienternas självbestämmande, öka delaktigheten och bidra till ett alliansbyggande.

Representanterna för hälso- och sjukvården samt brukarorganisationerna uttryckte å andra sidan, en farhåga för att användning av instrument riskerar att utarma eller kanske till och med ersätta det professionella samtalet. Ett mekaniskt fråga–svar-förfarande baserat på ett skattningsinstrument, kan till exempel minska patientens involvering i processen. En annan risk är om samtalets innehåll styrs av bedömaren, så att patientens möjligheter till delaktighet och samtycke begränsas. Den redan ojämlika relationen mellan patient och bedömare kan då förstärkas med negativa effekter på självbestämmande, delaktighet och förtroende.

En annan aspekt att ta hänsyn till är patienters och sjukvårdspersonals attityder till användning av skattningsinstrument. I dagsläget saknas svenska forskningsdata på detta område, men studier från USA indikerar att såväl patienter inom psykiatrin som på somatiska akutmottagningar tycks ha en positiv inställning till att sjukvårdspersonal ställer frågor eller använder screeninginstrument rörande risk för suicid [27,31,32].

Hos sjukvårdspersonal kan det finnas en oro att själva förfarandet med att fråga efter riskfaktorer för suicid, skulle kunna öka risken för att personen senare försöker ta sitt liv. Det finns dock inget stöd för detta i den vetenskapliga litteraturen, tvärtom tyder studierna på att samtal med patienter om suicid faktiskt kan minska risken [33].

Värde för diagnostiken

Den kliniska bedömningen är förenad med två allvarliga problem: att risken för suicid under- eller övervärderas. En undervärdering innebär att en person med förhöjd risk att begå suicid erbjuds otillräckliga insatser från sjukvården, med den potentiella konsekvensen att personen tar sitt liv eller på annat sätt kommer till skada. En övervärdering å andra sidan, innebär risk för att alltför långtgående åtgärder. Ytterst kan det röra sig om psykiatrisk tvångsvård, vilken tar ifrån patienten möjligheten att själv besluta om eller att motsätta sig vård [30]. I ett större perspektiv kan överanvändning av tvångsvård även hota allmänhetens förtroende för sjukvården.

Det uppställda kravet i denna översikt, att sensitiviteten skulle överskrida 80 procent för respektive instrument, kan anses vara ett lågt ställt krav, det betyder att man ändå inte identifierar var femte individ som sedan gör en suicidhandling. Kravet på minst 50 procent specificitet är också lågt ställt när det gäller att värdera den prediktiva förmågan hos instrumentet. Det innebär att instrumentet bara är något bättre än slumpen. Ur ett etiskt perspektiv är resultatet av översikten, att inget instrument trots de måttliga kraven befanns ha tillfredsställande känslighet och träffsäkerhet, ett skäl att ifrågasätta användningen av skattningsinstrument. Instrumentens brister motiverar starkt att bedömningen vilar på den sammantagna kliniska intervjun och bedömningen.

Flera riktlinjer och vårdprogram rekommenderar att skattningsinstrument används som komplement till den kliniska bedömningen [34]. Skattningsinstrument har utvecklats i förhoppningen att de ska bidra till att skärpa diagnostiken men i och med att instrumenten saknar stöd för tillförlitlighet försvagas dock argumenten avsevärt för att de ska användas.

Kvalitetssäkring

En utgångspunkt för sjukvården i Sverige är att patienter med samma sjukdomstillstånd bör erbjudas samma utredning och behandling, oavsett individuella faktorer såsom ålder, kön, etnicitet, ställning i samhället eller sjukdomsorsak [35]. I praktiken varierar dock tillgången på sjukvård regionalt, men även inom en och samma enhet kan det finnas skillnader mellan bedömare, till exempel på grund av olika utbildning och erfarenhet.

Bedömarens kliniska kompetens och förmåga att skapa ett öppet samtalsklimat har avgörande betydelse för rätt beslut och/eller diagnos. När skattningsinstrument används som stöd utgör de en checklista för bedömaren. De säkerställer att vissa frågor blir ställda, oavsett bedömarens tidigare erfarenhet eller kompetens. Genom att införa krav på att den kliniska bedömningen ska kompletteras med ett visst skattningsinstrument, kan en vårdgivare försäkra sig om att bedömningen har ett visst innehåll, oavsett vem som utför den. Skattningsinstrumentet kan ur denna aspekt bli en kvalitetssäkrande åtgärd. Även för den enskilda bedömaren kan den dokumenterade användningen av skattningsinstrument utgöra en trygghet vid exempelvis ansvarsärenden, då instrumenten kan ses som ett relativt objektivt mått på hur bedömningsförfarandet gått till.

Användning av skattningsinstrument vid suicidriskbedömning skulle dels kunna motiveras utifrån ett verksamhetsorienterat perspektiv och dels utifrån den enskilda sjukvårdspersonalens perspektiv. Mot detta kan argumenteras att alltför styrande riktlinjer minskar bedömarens möjligheter att anpassa bedömningssituationen till den enskilda patientens möjligheter och behov.

Konsekvens för vården

Ur patientens perspektiv är det viktigt hur instrumenten tillämpas. Om de integreras i ett samtal där intervjuaren framför allt är angelägen om att ge utrymme för patientens beskrivning och uppfattning av situationen, kan de upplevas som en väg att få mer information med kvalitetssäkrat innehåll. Instrumenten behöver studeras vidare innan man kan ta ställning till deras betydelse för vården av suicidnära patienter.

9. Praxis

Användning av suicidskattnings-instrument i Sverige

Det finns idag ingen nationell konsensus när det gäller användning av specifika skattningsskalor för att bedöma suicidrisk. Rutiner för bedömning av suicidnära personer kan variera stort klinikerna emellan. Svenska Psykiatriska Föreningens riktlinjer för utredning och behandling av suicidnära patienter tar upp tre skalor:

- Beck's Suicide Intent Scale (SIS), för analys av omständigheter vid aktuellt suicidförsök.
- Suicide Assessment Scale (SUAS), för skattning av symtomatologi oberoende av psykiatrisk diagnos.
- Columbia Suicide Severity Rating Scale (C-SSRS) för analys av intention och beteende, inklusive icke-suicidal självskada.

Praxisundersökningen bygger dels på en retrospektiv genomgång av händelseanalyser av anmälningar enligt Lex Maria, om personer som haft vårdkontakter i anslutning till att de avlidit till följd av suicid och dels på enkäter till läkare.

Analys av Lex Maria-anmälningar om suicid

Enligt rådande lagstiftning ska alla suicid som sker i samband med sjukhusvård eller öppenvård, eller inom fyra veckor av sådan vård, rapporteras enligt Lex Maria. Händelseanalyser av dessa fall kan ge en detaljerad beskrivning av vårdkontakter under tiden närmast före dödsfallet. Under åren 2009–2013 inkom till Patientsäkerhetsenheten i Västra Götalandsregionen händelseanalyser för 114 fall av suicid som ägt rum i anslutning

till vuxen psykiatrisk vård. Nästan två tredjedelar var män och åldersspannet var 20–92 år, med en medelålder på 43 år hos båda könen. Diagnoser redovisades i 110 av fallen. Över hälften hade förstämningssyndrom (56 %), en tredjedel en beroendediagnos och en tiondel psykos. Drygt hälften (53 %) hade gjort ett eller flera tidigare suicidförsök.

De flesta suicid (69 %) ägde rum i samband med psykiatrisk öppenvård, medan en femtedel (19 %) skedde under slutenvårdsbehandling. Nio vårdades enligt lagen om psykiatrisk tvångsvård (LPT) vid tiden för dödsfallet. En liten grupp (5 %) hade haft kontakt enbart med psykiatrisk akutmottagning.

Händelseanalyserna visade att suicidskattningsinstrument hade använts vid sista vårdkontakt för sex patienter (5 %). De instrument som används var SIS för ett fall, SAD PERSONS Scale i två fall och för de övriga tre framgick inte vilket skattningsinstrument som hade använts. I drygt en tredjedel av händelseanalyserna (36 %) fanns en notering att ingen skattningsskala hade använts vid sista vårdkontakten. I nästan en tredjedel av händelseanalyserna (32 %) fanns en rekommendation om att använda suicidskattningsinstrument i framtiden. I detta kliniska material användes sällan skattningsinstrument. Attityder hos sjukvårdspersonalen kan ha spelat in liksom andra förklaringar som tidsbrist, bristande kunskap kring vilka instrument som är tillämpliga och bristande tillit till instrumentens prediktionsförmåga. Det senare framkom i en kvalitativ studie där 15 specialister i psykiatri intervjuades om sina erfarenheter av bedömning av suicidnära patienter [36]. Bedömningsinstrument betraktades av några som mindre användbara (”…det går liksom inte att göra en algoritm för det här…”). Den globala bedömningen framhölls som central ”hur man observerar en persons ögonkontakt, affekter alltihop…blir ett helhetsintryck…det är väl det som gör det här att vilken trovärdighet man ger till personens ord………det personen säger och hur den personen är i kontakten”.

Mindre erfarna läkare kan vara mer benägna att uppfatta skalorna som ett stöd i kliniskt arbete. I en enkätundersökning som riktades mot unga läkare som nyligen påbörjat jourarbete på en psykiatrisk akutmottagning höll 61 procent med om påståendet att "suicidskattningsskalor förbättrar klinisk bedömning". Frågan var generellt ställd; inga specifika skalor nämndes [37].

Enkätundersökningar

Vi genomförde en enkätundersökning för att få en uppfattning om i vilken utsträckning samt vilka instrument som används vid bedömning av suicidrisk av läkare inom specialistpsykiatri och primärvård. Enkäten (Bilaga 5) delades ut vid tre tillfällen: Fortbildningsdagar för ST-läkare i allmänmedicin som anordnades av Dagens Medicin, oktober 2014; ST- kongressen som anordnades av Sveriges Läkare under Utbildning i Psykiatri (SLUP) i januari 2015 samt vid Svenska Psykiatrikongressen i mars 2015. Enkäten skickades även ut till allmänläkare i Jämtland samt till läkare inom BUP, Stockholm.

Svarsfrekvensen från ST-kongressen var hög (svar från 151/154 deltagare) medan svarsfrekvensen från övriga tillfällen låg mellan 20 och 30 procent. Vi redovisar därför enbart resultaten från ST-kongressen, då det är osäkert hur representativt urvalet är för de övriga tillfällena.

Av de 151 personer som besvarade enkäten, hade 145 svarat på fråga 1: "Använder du bedömningsinstrument som tillägg till den kliniska bedömningen vid suicidriskbedömning?" (Figur 9.2). Nästan samtliga (142/145, vilket utgör 98 %) av ST-läkarna använde instrument, varav 66 procent uppgav att de använder dem ofta (Figur 9.1). Av de som inte hade svarat på frågan (6 personer) hade alla svarat på följdfrågan och angivit vilka instrument de använt. En slutsats man kan dra av detta är att de använder instrument men att de svarsalternativ som gavs sällan eller oftast inte stämde in på dem.

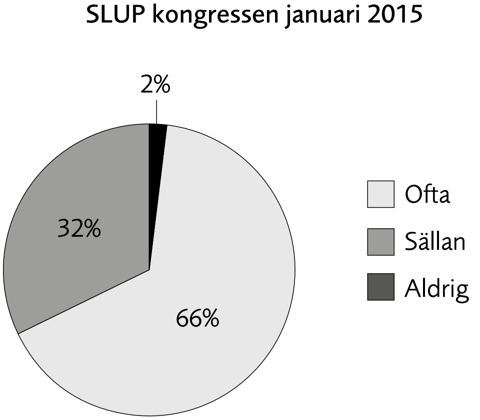

Figur 9.1 Andelen ST-läkare som använder instrument för suicidriskbedömning. Enkäten delades ut till 154 personer varav 145 personer svarade på frågan "Använder du skattningsskalor?". Svarsalternativen som gavs var ofta, sällan och aldrig.

Det framkom från enkäten att det var vanligare att använda formulär som inte är specifikt utvecklade för suicidriskbedömning (Figur 9.2). Nittiofem procent (143/153) uppgav att de använder depressionsskalan MADRS för suicidriskbedömning, det vill säga den fråga som mäter livslust. Näst vanligast var suicidmodulen i den strukturerade intervjun MINI (Figur 9.2). Detta mönster skilde sig inte åt mellan de läkare som ofta och de som sällan använde instrument för suicidriskbedömning. Cirka hälften uppgav att de använde instrument som är avsedda för suicidriskbedömning; SAD PERSONS Scale respektive SIS.

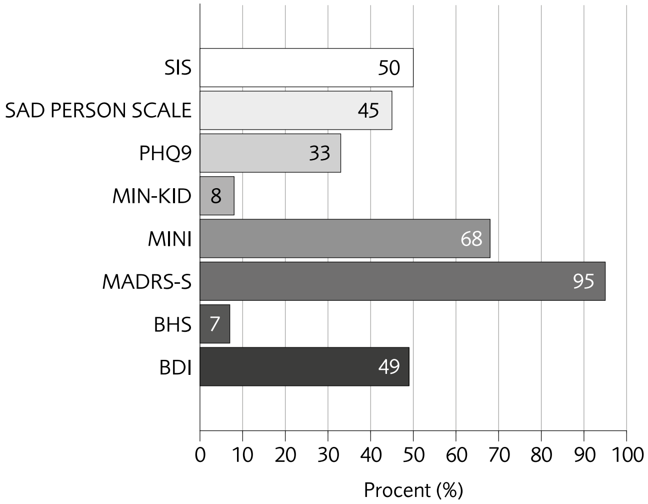

Figur 9.2 Fördelning av de instrument eller skalor som används av ST-läkare. På frågan "Har du använt följande skalor?" svarade 151 av 154 deltagare.

SUAS, HAD (Hospital Anxiety and Depression scale) och C-SSRS är andra instrument som verkar vanligt förekommande då de ofta nämndes i fritextrutan ”andra instrument” i enkäten.

Samma svarsmönster i andelen som använder sig av instrument vid suicidriskbedömning och vilka instrument som används kunde ses i enkätsvaren från övriga mätningar: Psykiatrikongressen, konferensen arrangerad av Dagens Medicin, BUP i Stockholm och distriktsläkare i Jämtland.

Sammantaget visar vår praxisundersökning att användningen av instrument för suicidriskbedömning bland ST-läkare är utbrett och att MADRS är det instrument som flest använder.

10. **Diskussion**

Metodfrågor

Vår översikt visar att det saknas vetenskapligt stöd för användning av suicidskattningsskalor för prediktion av suicidförsök och suicid. Innan resultaten diskuteras vidare finns ett antal metodfrågor som behöver belysas.

Deltagare

Deltagare i de ingående studierna rekryterades från vitt skilda vårdmiljöer. Inte sällan var deltagarna starkt selekterade, till exempel var de rekryterade från specialiserade avdelningar eller mottagningar. En del av studierna inkluderade endast tonåringar, andra fokuserade på vuxna personer med vissa personlighetsstörningar. Många av studierna inkluderade personer som skadat sig själva oavsett om det fanns en suicidavsikt eller inte; andra studier rekryterade deltagare bland de som vårdats i samband med allvarliga suicidförsök. För många av studierna framgick inte hur stor andel av de som tillfrågades som tackade ja, vilket gör det omöjligt att bedöma om studiegruppen var representativ.

Det är viktigt att komma ihåg att det inte finns någon ”typisk” studiedeltagare. Inom gruppen personer som gör suicidförsök eller dör genom suicid finns en hög grad av heterogenitet. I en och samma studie kan det ingå både personer som gör sitt livs första suicidala handling och personer som överlevt flera suicidförsök. En suicidal process kan pågå under några veckor eller månader eller under flera decennier. När det gäller psykiatriska diagnoser kan bilden variera stort både mellan och inom studierna. En del av rapporterna fokuserar specifikt på personer med egentlig depression, men ofta saknas information om samsjuklighet. Särskilt relevant i sammanhanget är alkoholmissbruk eller beroende, som är kopplat till en tiofaldig riskökning för död till följd av suicid jämfört med den allmänna befolkningen [38].

För att en studie skulle inkluderas i vår översikt krävdes minst 50 deltagare. Suicid är ett relativt ovanligt utfall och antalet deltagare som behövs för att upptäcka skillnader mellan grupper (i det här fallet de som i framtiden gör/inte gör en suicidal handling), beror delvis på om man utgår från en högriskpopulation eller inte. Vid analys av data från studier med relativt få deltagare såg man ofta breda konfidensintervall som talar för att osäkerheten är stor. För att handskas med denna problematik krävde vi att den nedre gränsen för konfidensintervallet för skalans tillförlitlighetsmått inte föll under vår förutbestämda värden för sensitivitet och specificitet.

Instrumenten

Ett stort antal skattningsskalor har granskats inom vårt arbete med översikten. Skalorna vilar på olika teoretiska ansatser, och det finns därmed en stor variation när det gäller vad som mäts och hur. För vissa skalor är det några enstaka statistiska riskfaktorer som undersökts, för andra kan det vara frågan om längre intervjuer som siktar på att fånga individens subjektiva tankar och känslor (Kapitel 2). Detta gör att en person kan bedömas ha en hög risk utifrån ett instrument och låg risk enligt ett annat, trots att instrumenten utåt sett handlar om samma sak. Detta framkom tydligt i en finsk studie där man använde fyra olika instrument för att identifiera suicidtankar hos personer med diagnosen egentlig depression [39]. Överensstämmelsen instrumenten emellan var bristfällig och problemet var ännu mer uttalat när man specifikt studerade den undergrupp som behandlades inom primärvården.

Instrumentens tillförlitlighet testades med avseende på ett visst tröskelvärde ("cut-off") och det är viktigt att komma ihåg att detta påverkar skalans prediktionsförmåga. Det kan vara svårt att jämföra resultat från två olika studier som utnyttjar samma instrument men som använder olika tröskelvärde. En del forskare använder sig av så kallade ROC-kurvor där det optimala tröskelvärdet bestäms av studiedata. Andra väljer en förutbestämd gräns. Hade författarna valt andra tröskelvärden är det möjligt att översiktens resultat skulle fått ett annat resultat.

Utfall

Våra resultat presenteras för två olika utfall: suicidförsök och suicid. En del av de ingående studierna använder ett kompositmått där suicidförsök med dödlig utgång inkluderas. Totalt analyserades data för 15 studier med data rörande risk för suicidförsök, och 10 med fokus på suicid. Vår översikt täcker inte studier som fokuserar enbart på självförvållad skada utan dödlig intention, till exempel självskada i affektreglerande syfte.

Uppföljningstid

Uppföljningstider varierar stort bland studierna i översikten, den kortaste gäller en månad och den längsta 19 år. Riskfaktorer för suicid bland personer som vårdas inom psykiatri skiljer sig i det korta och långa perspektivet [40]. Studier med kort uppföljningstid har större relevans för kliniska verksamheter. Ett år, som till exempel var uppföljningstid för PHQ-9-studien, är en relativt lång tid ur ett kliniskt perspektiv. Korttidsstudier är resurskrävande eftersom dessa kräver fler patienter på grund av utfallets låga bastal.

Krav på tillförlitlighet

I vår översikt var kravet att ett instrument för användning inom psykiatrisk vård skulle ha en sensitivitet >80 procent och en specificitet >50 procent. Vi valde en högre nivå för sensitivitet på grund av de allvarliga konsekvenser som ett falskt negativt utfall skulle innebära, nämligen att en person med hög risk för suicid inte identifieras som sådan. Med ett krav på sensitivitet >80 procent har vi ändå ”accepterat” att en femtedel av de som senare dör i suicid inte har kunnat identifieras i förväg med hjälp av instrumentet. För användning inom primärvården hade vi samma nivå för sensitivitet men inga krav på specificiteten. Detta motiveras av att man i screeningssammanhang kan acceptera fler falskt positiva fall (personer identifieras att vara i riskzonen utan att egentligen vara det). Hade vi valt andra nivåer för tillförlitligheten, eller låtit bli att ställa kravet att hela dess konfidensintervall skulle ligga inom acceptabelt nivå, är det möjligt att våra resultat hade sett annorlunda ut. Vidare bör nämnas att vi hade kunnat ställa krav på övriga precisionsmått (PPV, NPV, AUC) i denna översikt. Vi valde dock att inte

ställa krav på dessa mått då data saknades för många av instrumenten som ingick. Positivt prediktivt värde (PPV) vägdes dock in i konsekvensbedömningen av de korta instrumenten använda i akutmedicinsk vård, i huvudsak konstruerade för att identifiera vilka individer som skulle bedömas vid en psykiatrisk konsultation.

När vi diskuterar skattningsinstrumentens tillförlitlighet är det viktigt att komma ihåg att instrumentet i regel är testat under naturalistiska kliniska förhållanden, snarare än under kontrollerade former. Detta innebär att deltagarna i en och samma studie kan ha fått vitt skilda behandlingsinsatser. Det är rimligt att tro att mer intensiva behandlingar har initierats för personer som uppfattas som ”suicidnära” (Kapitel 2, Faktaruta 2.1). Detsamma gäller patientsäkerhetsåtgärder såsom inläggning och även i vissa fall behandling mot patientens vilja. Av etiska skäl kan instrumenten inte testas med en ”optimal” studiedesign; alla suicidnära erbjuds aktiva vårdinsatser.

Diskussion av fynden

Vår granskning av befintliga studier visar alltså att det saknas vetenskapligt stöd för användning av skattningsskalor för riskprediktion inom psykiatriska verksamheter, samt att det finns vetenskapligt stöd för att vissa instrument inte bör användas för prediktion såsom SAD PERSONS Scale, ERRS och Modified SAD PERSONS Scale. För vissa skalor, till exempel SAD PERSONS Scale kan vi konstatera att mer forskning sannolikt inte skulle förändra bilden. För andra skalor är det uppenbart att mer forskning behövs. Columbia Suicide Severity Rating Scale kan nämnas som särskilt intressant i sammanhanget då skalan på kort tid har fått en stor utbredning internationellt, både vid klinisk verksamhet och vid läkemedelsprövningar. Det vetenskapliga underlaget är dock som framgått ovan otillräckligt det vill säga, vi vet inte hur tillförlitlig den är.

Merparten av studierna kommer från Nordamerika och Europa, endast ett par genomfördes i Sverige. Det kan därför diskuteras om översiktens resultat är helt överförbart till svenska förhållanden. Suicidalt beteende är ett komplext fenomen; det kan finnas betydande kulturella skillnader när det gäller hur suicidalt beteende definieras och avseende samhällets

syn på suicidhandlingar. Skilda socioekonomiska faktorer påverkar både individens livsvillkor, vårdens utbud och tillgänglighet, och suicidpreventiva insatser på samhällsnivå. Allt detta kan bidra till de stora variationerna i förekomsten av suicidalt beteende runt om i världen. Trots detta har forskare kunnat konstatera att riskfaktorer för suicidalt beteende är snarlika i ett globalt perspektiv [41]. Det senare kan ge indirekt stöd för att översiktens resultat kan vara av intresse även för svenska förhållanden.

Under vårt arbete med översikten noterade vi att det saknades relevanta studier för utvärdering av MADRS/MADR-S suicidfråga. Här finns en viktig kunskapslucka då skalan används relativt ofta inom den psykiatriska vården. När det gäller instrument som utvecklats för barn- och ungdomspsykiatri saknades studier som utvärderar MINI KID-suicidmodulen. Samma resonemang gäller för utvärdering av suicidfrågan som ingår i Edinburgh Depression Scale, en skala som används rutinmässigt för screening vid många av landets mödravårdscentraler.

Jämförelser med andra systematiska översikter

I en sammanställning av riskfaktorer för upprepad självskada gjordes en genomgång av nio suicidskattningsinstrument [42]. Studiens omfattning var mindre än vår översikt; man undersökte endast ett urval av instrument som specifikt analyserade risk för repetition bland personer som sökte vård i samband självskada (förgiftning eller självtillfogad skada oavsett avsikt). Studier som fokuserade enbart på instrumentens förmåga att förutse fullbordade suicid exkluderades. Kompositskalor tenderade att ha hög sensitivitet men låg specificitet i denna analys. Trots metodologiska skillnader kan man konstatera att vår översikt gav ett snarlikt resultat.

I USA gjordes nyligen en omfattande genomgång över screeningsinstrument för suicidrisk lämpliga för användning i primärvården [43]. Man fann begränsat stöd för att några screeninginstrument kunde identifiera vuxna med ökad suicidrisk, men konstaterade att samtliga instrument med sensitivitet ≥80 hade ett positivt prediktivt värde under 40. I vår analys hade vi inget krav på specificiteten för screeningsinstrument,

varför vi bedömde att suicidfrågan inom depressionsskalan PHQ-9 kan vara relevant för screening i primärvården.

I en kanadensisk rapport presenterades en icke-systematisk kritisk granskning av 15 vanligt förekommande suicidskattningsinstrument [44]. Till skillnad från vår rapport gjordes ingen formell evidensgradering och ingen metaanalys. Varje instrument beskrivs med avseende på bland annat vad som mäts och hur, inklusive poängsättning, lämplig vårdnivå och instrumentets psykometriska egenskaper. Författarna poängterar att inga av de ingående instrumenten kan ersätta en klinisk suicidriskbedömning. Det som gör skalan till ett värdefullt komplement är dess förmåga att ibland kunna bidra med ny information, vilket kan vara av särskilt värde för oerfarna behandlare.

11. Konsekvenser och kunskapsluckor

Praktiska konsekvenser

Kunskapsläget är att inget av de undersökta instrumenten som hade tillräcklig vetenskapligt underlag (begränsat–starkt) har visat sig vara kliniskt användbart för prediktion inom specialistvården. För några av instrumenten som till exempel SUAS och C-SSRS var det vetenskapliga underlaget otillräckligt, vilket innebär att det i nuläget är oklart om de är tillförlitliga och därmed kliniskt användbara. De instrument som värderats har använts inom olika kliniska populationer, som till exempel personer som sökt psykiatrisk vård eller, mer selekterat, sökt efter att ha gjort självskada/suicidförsök. Utfallet som studerats prospektivt har varit suicidförsök, upprepat suicidförsök eller suicid. Med de rimliga kraven för bedömning av suicidrisk som ställdes för specialistvård (att ett instrument inte ska missa mer än 20 procent av fallen), fanns det inget instrument som har tillräckligt goda prediktiva egenskaper. Studiens resultat visar med all tydlighet hur svårt det är att ”träffa rätt” med skalorna, en kunskap som är viktigt inte bara för vårdgivare utan även för tillsynsmyndigheter som kopplas in när suicid sker i samband med vården.

En praktisk konsekvens kan gälla ett av de instrument som används på flera kliniker inom psykiatri, SAD PERSONS Scale. I de två studierna av detta instrument fann man en mycket låg sensitivitet (tröskelvärde >7) och det vetenskapliga underlaget för detta var starkt. Detta är allvarligt på det sätt att en stor majoritet av de personer som senare begår suicidhandlingar inte identifieras. Konsekvensen blir att starkt ifrågasätta relevansen av att använda denna skala för prediktion. Samma skeptiska bedömning görs i en färsk systematisk översikt av SAD PERSONS Scale, men författarna till den översikten menar att bristen på evidens betyder att det inte finns tillräcklig information för att bestämt avråda från använda av detta instrument [45]. För undervisning kan instrumentet ha ett berättigande.

För triagering (i det här fallet att identifiera patienter med suicidrisk inom akutmedicinsk vård), fanns några korta instrument med hög sensitivitet. Dessa instrument (Manchester Self Harm Rule, MSHR, med en modifierad variant kallad ReACT och Södersjukhuset Self Harm Rule, SoS-4), hade en sensitivitet på 90 procent eller mer, men däremot mycket låg specificitet. De hade positivt prediktivt värde (PPV) på 20–37 procent avseende upprepad självskada i de populationer de studerats men saknade visserligen större värde för prediktion. Nyttan av dem skulle dock vara, att i det ofta stora inflödet av akutmedicinska patienter, kunna identifiera de personer som bör bedömas av psykiaterkonsult på grund av suicidrisk. Man kan dock överväga om instrumenten tillför något för triagering då gällande kliniska riktlinjer rekommenderar att alla som gjort suicidförsök eller självskada bör bli föremål för en psykiatrisk bedömning [46].

För patienter i primärvård undersöktes ett depressionsinstrument avseende förmåga att predicera en suicidhandling, PHQ-9, vilket innehåller en fråga om suicidtankar eller suicidal intention. Här fanns ett måttligt vetenskapligt stöd för att personer som svarade ja på denna fråga utförde ett suicidförsök under det följande året. Tolkningen av denna studie är att det finns ett samband mellan depression med suicidtankar och senare suicidhandling. Detta betyder att frågan är relevant för screening av deprimerade patienters risk för suicidförsök och suicid. Däremot räcker det inte, vid depression, med ett positivt svar på denna enda fråga för att kunna förutsäga en suicidhandling. Det krävs naturligtvis en mer ingående undersökning av patientens symtombild, sociala situation, och tidigare och aktuella suicidala beteende. För den ofta använda depressionsskalan MADRS fanns inga studier som gällde instrumentets motsvarande fråga om döds- eller suicidtankar, vilket i i detta avseende saknar evidens.

Flera andra ofta rekommenderade skattningsinstrument som SIS, SSI och SUAS har inte visat sig ha tillräckligt vetenskapligt stöd. Dessa instruments värde har belysts i de riktlinjer som utvecklats av National Institute for Health and Clinical Excellence (NICE) i Storbritannien [47]. Här påtalas denna typ av instruments oförmåga att predicera suicid och andra former av självskador. Man menar att personer som söker vård

i samband med självskada istället bör genomgå en riskbedömning som fokuserar på kliniska och demografiska faktorer samt psykologiska fenomen såsom depression, hopplöshet och suicidal intention samt att man bör göra en inventering av personens individuella behov. Riskfaktorer och psykosociala behov integreras i bedömningsprocessen som ligger till grund för fortsatt behandling.

Bristen på värde för prediktion behöver i sig inte betyda att man ska utmönstra dessa instrument från användning i sjukvården. De kan ha ett särskilt utbildningsvärde, till exempel att lära ny eller oerfaren personal att efterfråga viktiga riskfaktorer för suicidhandling [48].

En av de aktuella frågor vi velat belysa är patientsäkerheten. Med detta menas att man i sjukvården förmår bedöma patientens tillstånd och risken för suicidhandling på ett adekvat sätt för att förhindra att suicid inträffar. Att värdera risken är också att identifiera de faktorer som man kan behandla. Det saknades dock studier som visar om patientsäkerheten förbättras om de granskade instrumenten används.

Det är viktigt att poängtera att denna systematiska översikt inte allmänt kan uttala sig om värdet av en suicidriskbedömning för de patienter som kommer till vården. Översiktens slutsatser är att intervju- och skattningsinstrument av den typ som undersökts, inte enbart själva kan identifiera de personer som sedan gör en mer eller mindre allvarlig suicidhandling. Framför allt bör man betona att översiktens resultat inte ifrågasätter värdet av att man gör en klinisk suicidriskbedömning. Tvärtom, eftersom intervju- och skattningsinstrument inte fungerar, blir det ännu viktigare att göra en omsorgsfull klinisk bedömning av den aktuella personens livssituation och sociala nätverk, tidigare och aktuella suicidavsikt samt av den kliniska sjukdomsbilden, inklusive missbruksproblematik.

Forskningskonsekvenser

Kunskapsläget gör att det vetenskapliga underlaget är otillräckligt eller begränsat för de studier som undersökt instrument för skattning av suicidrisk i psykiatriska populationer. Det är ett fåtal av de undersökta

studierna som klart anger var i den suicidala processen undersökningspersonen befinner sig vid inklusion i studien. Det kan vara en avsevärd skillnad för det fortsatta beteendet om en person gör sitt första suicidförsök eller redan utfört ett flertal.

Det finns visserligen några relativt stora studier men oftast bara enstaka studier för varje instrument. Flertalet har också en lång observationstid vilket kan tyckas värdefullt (flera års observation), men när det gäller bedömning av suicidrisk är ett instrument mer värdefullt om det betyder något för värderingen av risken för suicidhandling på kort sikt. Detta kan anses som en kunskapslucka, det vill säga, det behövs större studier med om möjligt kortare tid mellan indexförsök och uppföljningstid. Flera av de granskade intervju- eller skattningsinstrumenten är vidare otillräckligt undersökta. Detta gäller de vanligen använda SSI, SIS, SUAS, BHS och det nyare och nu ofta rekommenderade C-SSRS.

Kunskapsläget är bättre för de mycket korta instrumenten som identifierar patienter med suicidrisk i akutmedicinsk vård som MSHR och SoS-4, och det finns inte lika stort behov av ytterligare studier av de angivna instrumenten för triagering.

En annan kunskapslucka gäller huruvida de ingående, eller eventuellt andra instrument, är tillämpliga i särskilda diagnostiska grupper. Endast ett fåtal av de studier som igår i denna översikt handlar om huruvida ett instrument har särskild relevans för till exempel gruppen av patienter med depressions-, psykossjukdom eller alkoholberoende. I detta avseende finns en kunskapslucka. Möjligen är det så att ett instrument kan fungera i vissa grupper och förutom diagnostiska grupper behöver man därför studera om de fungerar bättre i olika köns- och åldersgrupper.

Få studier handlar om patientperspektivet och det är därför värdefullt att undersöka hur patienter upplever hur det är att genomgå en strukturerad skattning av suicidrisk. Vidare identifierades inga studier som handlade om huruvida patientsäkerheten förbättras om instrument används som komplement till den kliniska bedömningen. Det är därför viktigt att framtida studier granskar detta område.

12. **Tabeller**

Table 12 Instruments for suicide risk assessment.

First author<br>Year<br>Country<br>Reference	Design<br>Setting<br>Population<br>Evaluation time	Index test<br>Reference test<br>Drop-out rate	Results<br>(sensitivity, specificity, PPV, NPV)	Comments<br>Risk of bias
**BDI**				
Beck et al<br>1990<br>USA<br>[6]	**Design**<br>Cohort<br><br>**Setting**<br>Center for cognitive therapy<br><br>**Population**<br>N=1 958, outpatients who were evaluated at intake. Excluded severe risk of suicide<br><br>**Participation rate**<br>NR<br><br>**Evaluation time**<br>Mean 43±20.71 months (until December 1985)<br><br>**Study period**<br>September 1978 to February 1985	**Index test**<br>BDI<br>n=1 958<br>Female: 58%<br>Mean age: 36.12±12.39 years (range 15–84)<br><br>**Reference test**<br>Suicide<br>n=17<br>Female: 47.1%<br>Mean age: 40.35±11.61 years (range 18–59)<br><br>**Drop-out rate**<br>14/1 958 (0.7%)<br>Natural death: 12	**Cut-off ≥23**<br>Sensitivity: 0.76 (0.50; 0.93)<br>Specificity: 0.62 (0.60; 0.65)<br>PPV: 1.75%<br>NPV: 99%	**Sampling method**<br>Consecutive patients<br><br>**Handling of missing data**<br>Two drop-outs were excluded from all further analyses<br><br>**Other comments**<br>10 of the 17 patient that committed suicide received cognitive therapy. Only one patient was in treatment at the time of his suicide
**BHS**				
Beck et al<br>1990<br>USA<br>[6]	**Design**<br>Cohort<br><br>**Setting**<br>Center for cognitive therapy<br><br>**Population**<br>N=1 958, outpatients who were evaluated at intake. Excluded severe risk of suicide<br><br>**Participation rate**<br>ND<br><br>**Evaluation time**<br>Mean 43±20.71 months (until December 31, 1985)<br><br>**Study period**<br>September 1978 to February 1985	**Index test**<br>BHS<br>n=1 958<br>Female: 58%<br>Mean age: 32.16±12.39 years (range 15–84)<br><br>**Reference test**<br>Suicide<br>n=17<br>Female: 47.1%<br>Mean age: 40.35±11.61 years (range 18–59)<br><br>**Drop-out rate**<br>2/1 958 (0.1%)<br>Natural death: 12	**Cut-off ≥9**<br>Sensitivity: 0.94 (0.71; 1.00)<br>Specificity: 0.41 (0.39; 0.43)<br>PPV: 1.38%<br>NPV: 99.9%	**Sampling method**<br>Consecutive patients<br><br>**Handling of missing data**<br>Two drop-outs were excluded from all further analyses<br><br>**Other comments**<br>10 of the 17 patient that committed suicide received cognitive therapy Only one patient was in treatment at the time of his suicide

The table continues on the next page

Table 12 continued

First author<br>Year<br>Country<br>Reference	Design<br>Setting<br>Population<br>Evaluation time	Index test<br>Reference test<br>Drop-out rate	Results<br>(sensitivity, specificity, PPV, NPV)	Comments<br>Risk of bias
Beck et al<br>1999<br>USA<br>[7]	**Design**<br>Cohort<br><br>**Setting**<br>Center of cognitive therapy, University of Pennsylvania<br><br>**Population**<br>N=3 701, outpatients who were evaluated at intake, 65% history of suicide attempts<br><br>**Participation rate**<br>ND<br><br>**Evaluation time**<br>1979–1994<br><br>**Study period**<br>1975–1994	**Index test**<br>BHS<br>n=3 701<br>Female: 56.7%<br>Mean age: NR<br><br>**Reference test**<br>Suicide<br>n=30<br>Female: 40%<br>Mean age: 41.1±13.68 years<br><br>**Drop-out rate**<br>128/3 701 (3%)	Cut-off: Derived from ROC analysis<br><br>**Cut-off for higher risk ≥8**<br>Sensitivity: 0.90 (0.73; 0.98)<br>Specificity: 0.42 (0.40; 0.44)<br>PPV: 1.3%<br>NPV: 100%	**Sampling method**<br>Few inpatients in the sample
Keller et al<br>1993<br>Germany<br>[8]	**Design**<br>Cohort<br><br>**Setting**<br>Depression ward, Psychiatric Hospital<br><br>**Population**<br>N=76, depressive illness, admitted to the ward, none with a secondary psychiatric diagnosis<br><br>**Participation rate**<br>NR<br><br>**Evaluation time**<br>1 year<br><br>**Study period**<br>February to October 1986	**Index test**<br>BHS<br>n=61<br>Female: 65.6%<br>Mean age: 47<br><br>**Reference test**<br>Suicide<br>n=2<br>% female: NR<br>Mean age: NR<br><br>Suicide attempts<br>n=8<br>% female: NR<br>Mean age: NR<br><br>**Drop-out rate**<br>15/76 (20%)	**Cut-off ≥9**<br>***Suicide acts***<br>Sensitivity: 0.80 (0.44; 0.97)<br>Specificity: 0.27 (0.16; 0.42)<br>PPV: 18%<br>NPV : 88%<br><br>***Suicide***<br>Sensitivity: 1.00 (0.16; 1.00)<br>Specificity: 0.27 (0.16; 0.40)<br>PPV: 4.4%<br>NPV: 100%	**Handling of missing data**<br>Unclear

The table continues on the next page

Table 12 continued

First author<br>Year<br>Country<br>Reference	Design<br>Setting<br>Population<br>Evaluation time	Index test<br>Reference test<br>Drop-out rate	Results<br>(sensitivity, specificity, PPV, NPV)	Comments<br>Risk of bias
Klonsky et al<br>2012<br>USA<br>[10]	**Design**<br>Cohort<br><br>**Setting**<br>12 inpatient facilities<br><br>**Population**<br>N=675<br>Patients with 1st admission of psychosis, 29% with a history of attempted suicide<br><br>**Participation rate**<br>72%<br><br>**Evaluation time**<br>Up to 10 years<br><br>**Study period**<br>1989–1995	**Index test**<br>BHS<br>n=414<br>Female: 43%<br>Mean age: 29.1±9.6 years<br><br>**Reference test**<br>Suicide<br>10 years: n=6<br>Female: 33%<br><br>**Drop-out rate**<br>261/675 (39%) at 10 years	**Cut-off ≥9**<br>Sensitivity: 0.33 (0.04; 0.78)<br>Specificity: 0.88 (0.84; 0.91)<br>PPV: 3.92%<br>NPV: 98.9%	**Other comments**<br>For the 6 persons that completed suicide, hopelessness at baseline ranged from 0–11, with a median of 4
Niméus<br>1997<br>Sweden<br>[9]	**Design**<br>Cohort<br><br>**Setting**<br>Suicide research unit at university hospital<br><br>**Population**<br>N=212,<br>Suicide attempters 18+,<br>84% history of suicide attempts<br><br>**Participation rate**<br>NR<br><br>**Evaluation time**<br>Range 1 month to 8 years<br><br>**Study period**<br>NR	**Index test**<br>BHS<br>n=212<br>Female: 56.6%<br>Mean age: 37.7±13.6 years (range 18–80)<br><br>**Reference test**<br>Suicide<br>n=13<br>Female: 69%<br>Mean age: NR<br><br>**Drop-out rate**<br>0	**Cut-off 9**<br>Sensitivity: 0.77 (0.46; 0.95)<br>Specificity: 0.42 (0.35; 0.49)<br>PPV: 8%<br>NPV: 96.5%<br><br>**Cut-off 13**<br>Sensitivity: 0.77<br>Specificity: 0.61<br>PPV: 13%	**Other comments**<br>Includes only patients referred to the suicide research unit<br><br>Suicides identified by local dept of forensic medicine, persons who moved from the area cannot be followed up

The table continues on the next page

Table 12 continued

First author<br>Year<br>Country<br>Reference	Design<br>Setting<br>Population<br>Evaluation time	Index test<br>Reference test<br>Drop-out rate	Results<br>(sensitivity, specificity, PPV, NPV)	Comments<br>Risk of bias
Sidley et al<br>1999<br>United Kingdom<br>[15]	**Design**<br>Cohort<br><br>**Setting**<br>Accident department and ED<br><br>**Population**<br>Parasuicide patients<br>N=66<br><br>**Participation rate**<br>ND<br><br>**Evaluation time**<br>12 months<br><br>**Study period**<br>NR	**Index test**<br>BHS<br>n=66<br>Female: 45.5%<br>Mean age: 33.6 years<br>(range 19–58)<br><br>**Reference test**<br>Parasuicide<br>1 months: n=10<br>6 months. n= 20<br>12 months: n=25<br>Female: NR<br>Mean age: NR<br><br>**Drop-out rate**<br>2/66 (3%) at 1 month<br>3/66 at 12 months	**Cut-off 19 or 20, 1 month**<br>Sensitivity: 0.60 (0.26; 0.88)<br>Specificity: 0.91 (0.80; 0.97)<br>PPV: 54.35%<br>NPV: 92.72%<br><br>**Cut-off ≥2, 12 months**<br>Sensitivity: 0.58 (0.37; 0.78)<br>Specificity: 0.87 (0.73; 0.96)<br>PPV: 73.12%<br>NPV: 77.26%	
**C-SSRS**				
Posner et al<br>2011<br>USA<br>[11]	**Design**<br>Cohort<br><br>**Setting**<br>NR<br><br>**Population**<br>N=124<br>Adolescent suicide attempters<br>Female: NR<br>Mean age: NR<br><br>**Evaluation time**<br>6–24 weeks<br><br>**Study period**<br>NR	**Index test**<br>C-SSRS<br>n=124<br><br>**Reference test**<br>Suicide<br>n=1<br><br>Suicide attempts<br>n=15 patients, 24 episodes<br><br>**Drop-out rate**<br>ND	Sensitivity: 100%<br>Specificity: 96%	

The table continues on the next page

Table 12 continued

First author<br>Year<br>Country<br>Reference	Design<br>Setting<br>Population<br>Evaluation time	Index test<br>Reference test<br>Drop-out rate	Results<br>(sensitivity, specificity, PPV, NPV)	Comments<br>Risk of bias
**ERRS**				
Carter et al<br>2002<br>Australia<br>[16]	**Design**<br>Cohort<br><br>**Setting**<br>Centralised referral center<br><br>**Population**<br>N=1 331<br>Self-poisoning patients/adults<br><br>**Evaluation time**<br>12 months<br><br>**Study period**<br>3 year period<br>1996–1998	**Index test**<br>ERRS<br>n=1 317<br>Female: 62.3%<br>Median age (F/M): 32/33 years<br><br>**Reference test**<br>Repeated DSP<br>n=180<br>Female: 63%<br><br>**Drop-out rate**<br>14/1 331 (1.1%)	**Cut-off Clinical scoring**<br>High risk, men ≥8<br>High risk, women ≥6<br>Sensitivity: 0.26 (0.20; 0.33)<br>Specificity: 0.84 (0.82; 0.86)<br>PPV: 21%<br>NPV: 88%<br><br>**Cut-off: research scoring**<br>Sensitivity: 0.27 (0.21; 0.34)<br>Specificity: 0.86 (0.84; 0.88)<br>PPV: 23%<br>NPV: 88%	**Sampling method**<br>Consecutive
**IAT**				
Randall et al<br>2013<br>Canada<br>[17]	**Design**<br>Cohort<br><br>**Setting**<br>2 ED<br><br>**Population**<br>>17 years<br>N=180<br><br>**Participation rate**<br>67%<br><br>**Evaluation time**<br>3 months<br><br>**Study period**<br>August 2009 to May 2010	**Index test**<br>Death/life IAT<br>n=127<br>Female: 47.8%<br>Mean age: NR<br><br>**Reference test**<br>Self-harm<br>n=29<br>Female: NR<br>Mean age: NR<br><br>**Drop-out rate**<br>20/127 (15.8%)	**Cut-off 0**<br>Sensitivity: 43.3% (0.255; 0.626)<br>Specificity: 78.8% (68.2; 87.1)<br>PPV: 37.7%<br>NPV: 82.5%<br><br>*According to RevMan:*<br>Sensitivity: 0.45 (0.26; 0.64)<br>Specificity: 0.78 (0.67; 0.87)<br>PPV: 43%<br>NPV: 79%<br>(n=29 of 107)	

The table continues on the next page

Table 12 continued

First author<br>Year<br>Country<br>Reference	Design<br>Setting<br>Population<br>Evaluation time	Index test<br>Reference test<br>Drop-out rate	Results<br>(sensitivity, specificity, PPV, NPV)	Comments<br>Risk of bias
**MINI**				
Roaldset et al<br>2012<br>Norway<br>[18]	**Design**<br>Cohort<br><br>**Setting**<br>Acute psychiatric unit<br><br>**Population**<br>N=489, all acutely admitted patients<br><br>**Participation rate**<br>411/489<br><br>**Evaluation time**<br>1 year<br><br>**Study period**<br>March 2006 to July 2008	**Index test**<br>MINI suicide item<br>n=411<br>Mean age: ND<br><br>**Reference test**<br>Suicide behaviour or NSSI<br>3 months: n=71<br>12 months: n=112<br>Suicide attempts or NSSI: n=64<br>Female: NR<br>Mean age: NR<br><br>**Drop-out rate**<br>1 year: 104/411 (25%)	**Self harm cut-off ≥10**<br>Sensitivity: 61%<br>Specificity: 75%<br>PPV: 38%<br>NPV: 86%	**Other comments**<br>Unclear whether the 2 suicides were included among the attempts in the index test. For multiple admissions, "index" subgroup was defined in retrospect as the most serious episode
**MSHR**				
Bilén et al<br>2013<br>Sweden<br>[25]	**Design**<br>Cohort<br><br>**Setting**<br>2 large ED<br><br>**Population**<br>Adults, DSH<br>N=328<br><br>**Participation rate**<br>ND<br><br>**Evaluation time**<br>6 months<br><br>**Study period**<br>Mars 2011 to June 2011	**Index test**<br>MSHR<br>n=325<br>Female: 70%<br>Mean age: NR<br><br>**Reference test**<br>Repeated DSH/ suicide attempts<br>n=80<br>Female: NR<br>Mean age: NR<br><br>**Drop-out rate**<br>3/328 (1%)	**Cut-off 0.14**<br>Sensitivity: 0.94 (0.85; 0.98)<br>Specificity: 0.18 (0.14; 0.24)<br>PPV: 27%<br>NPV: 90%	**Sampling method**<br>Consecutive

The table continues on the next page

Table 12 continued

First author<br>Year<br>Country<br>Reference	Design<br>Setting<br>Population<br>Evaluation time	Index test<br>Reference test<br>Drop-out rate	Results<br>(sensitivity, specificity, PPV, NPV)	Comments<br>Risk of bias
Bilén et al<br>2013<br>Sweden<br>[26]	**Design**<br>Cohort<br><br>**Setting**<br>ED<br><br>**Population**<br>N=1 524<br>Adults, DSH<br>History of DSH: 35%<br>Treated with antidepressants: 57%<br><br>**Evaluation time**<br>6 months<br><br>**Study period**<br>January 2003 to December 2005	**Index test**<br>MSHR<br>n=1 524<br>Female: 65%<br>Mean age: NR<br><br>**Reference test**<br>Suicide + repeated DSH<br>n=309<br>Female: 70%<br>Mean age: 39.5 years<br>(range 18–91 years)<br><br>Suicide<br>n=12<br>Repeated DSH<br>n=297<br><br>**Drop-out rate**<br>0%	**Cut off: 0.14**<br>Repeated DSH<br>Sensitivity: 0.89 (0.85; 0.92)<br>Specificity: 0.21 (0.19; 0.24)<br>PPV: 22% (275/1 230)<br>NPV: 88% (260/294)	**Sampling method**<br>Consecutive sample
Cooper et al<br>2006<br>United Kingdom<br>[23]	**Design**<br>Cohort<br><br>**Setting**<br>5 ED<br><br>**Population**<br>N=11 819 episodes<br>Self harm<br><br>**Evaluation time**<br>6 months<br><br>**Study period**<br>September 1997 to February 2001	**Index test**<br>MSHR<br>n=6 933 patients,<br>9 086 episodes<br>Derivation set:<br>n=6 933 episodes<br>Validation set:<br>n=2 153 episodes<br>Mean age: NR<br><br>**Reference test**<br>Suicide attempts/self-harm<br>n=1 538 episodes<br>Median age: 32 years<br><br>Suicide<br>6 months: n=22<br>3 years: n=59<br><br>**Drop-out rate**<br>Derivation data set: 203 (3%)<br>Validation data set: 58 (3%)	**Cut-off Episodes**<br>Derivation data set<br>Sensitivity: 0.94 (0.92; 0.95)<br>Specificity: 0.25 (0.24; 0.26)<br>PPV: 20 (19; 21)<br>NPV: 95 (94; 96)<br><br>Validation data set<br>Sensitivity: 0.97 (0.94; 0.98)<br>Specificity: 0.26 (0.24; 0.29)<br>PPV: 22 (19; 21)<br>NPV: 97 (96; 99)	**Other comments**<br>The study used internal validation

The table continues on the next page

Table 12 continued

First author<br>Year<br>Country<br>Reference	Design<br>Setting<br>Population<br>Evaluation time	Index test<br>Reference test<br>Drop-out rate	Results<br>(sensitivity, specificity, PPV, NPV)	Comments<br>Risk of bias
Randall et al<br>2012<br>Canada<br>[24]	**Design**<br>Cohort<br><br>**Setting**<br>2 ED<br><br>**Population**<br>N=181<br>Adult patients presenting with self harm or suicidal ideation<br><br>**Evaluation time**<br>3 months<br><br>**Study period**<br>August 2009 to May 2010	**Index test**<br>MSHR<br>n=157<br>Female: 48.9%<br>Mean age: 37.2 years<br><br>**Reference test**<br>Self-harm<br>n=34<br>Female: NR<br>Mean age: NR<br><br>**Drop-out rate**<br>29/157 (18.5%)	**Cut-off 1**<br>Sensitivity: 95.1%<br>Specificity: 0.15 (0.09; 0.22)<br>PPV: 23.70%<br>NPV: 91.6%	
Steeg et al<br>2012<br>United Kingdom<br>[14]	**Design**<br>Cohort<br><br>**Setting**<br>5 ED<br><br>**Population**<br>Adults, self-harm<br>N=18 680 patients,<br>29 571 presentations<br><br>**Evaluation time**<br>6 months<br><br>**Study period**<br>January 2003 and June 2007	**Index test**<br>MSHR<br>n=24 779 presentations<br>Female: NR<br>Median age: NR<br><br>**Reference test**<br>Suicide<br>n=76<br>Female: 39%<br>Mean age: NR<br><br>Suicide attempts/self-harm<br>n=7 606 episodes<br>Female: NR<br>Mean age: NR<br><br>**Drop-out rate**<br>Unclear	**Suicide attempts**<br>***Derivation centre***<br>Sensitivity: 0.98 (0.98; 0.99)<br>Specificity: 0.17 (0.16; 0.17)<br>PPV: 33.5%<br>NPV: 95.2%<br><br>***Test centre***<br>Sensitivity: 0.97 (0.96; 0.98)<br>Specificity: 0.20 (0.18; 0.21)<br>PPV: 37.5%<br>NPV: 93%	

The table continues on the next page

Table 12 continued

First author<br>Year<br>Country<br>Reference	Design<br>Setting<br>Population<br>Evaluation time	Index test<br>Reference test<br>Drop-out rate	Results<br>(sensitivity, specificity, PPV, NPV)	Comments<br>Risk of bias
**PHQ-9**				
Simon et al<br>2013<br>USA<br>[5]	**Design**<br>Cohort<br><br>**Setting**<br>Primary care and mental health clinics within an integrated health care system<br><br>**Population**<br>Depression, age 13+<br>N=84 418 patients<br>188 611 (90%) of patients were receiving current/recent mental health treatment<br><br>**Participation rate**<br>Unclear<br>Data collected at 5% of depression visits in 2007 and 48% in 2011<br><br>**Evaluation time**<br>Range 1–1 703 days, median 360, mean 477<br><br>**Study period**<br>January 2007 to December 2011	**Index test**<br>PHQ-9 item 9<br>n=84 418<br><br>**Reference tests**<br>Suicide<br>n=46<br>Female: NR<br>Mean age: NR<br><br>Suicide attempts<br>n=709<br>Female: NR<br>Mean age: NR<br><br>**Drop-out rate**<br>24%	**Cut-off Several days during the last 2 weeks**<br>Suicide<br>Sensitivity: 0.80 (0.66; 0.91)<br>Specificity: 0.70 (0.70; 0.71)<br>PPV: 0.15%<br>NPV: 97.8%<br><br>Suicide attempts<br>Sensitivity: 0.78 (0.74; 0.81)<br>Specificity: 0.71 (0.70; 0.71)<br>PPV: 2%<br>NPV: 99.76%	**Other comments**<br>Multiple ratings (207 265 records), analyses are based on questionnaire completed closest to event date.<br>Events determined by electronic records, insurance claims and death certificates<br><br>29% of suicide attempts missed by E codes

The table continues on the next page

Table 12 continued

First author<br>Year<br>Country<br>Reference	Design<br>Setting<br>Population<br>Evaluation time	Index test<br>Reference test<br>Drop-out rate	Results<br>(sensitivity, specificity, PPV, NPV)	Comments<br>Risk of bias
**ReACT**				
Steeg et al<br>2012<br>United Kingdom<br>[14]	**Design**<br>Cohort<br><br>**Setting**<br>5 hospital EDs<br><br>**Population**<br>Adults, Self-harm<br>N=18 680 patients,<br>29 571 presentations<br>Female: 59%<br>Median age: 32 years<br>(range 16–97)<br><br>**Evaluation time**<br>6 months<br><br>**Study period**<br>January 2003 and June 2007	**Index test**<br>ReACT Self-Harm Rule<br>Derivation set:<br>n=22 532 episodes<br>External test data:<br>n=7 039 episodes<br><br>**Reference test**<br>Suicide<br>Derivation set: n=66<br>External test data: n=26<br>Total: n=92<br>Female: 39%<br><br>Suicide attempts/self-harm<br>Derivation set:<br>n=6 014 episodes<br>External test data:<br>n=2 096 episodes<br><br>In total:<br>Female: 63%<br>Mean age: NR<br><br>**Drop-out rate**<br>NR	**Any repetition/suicide attempts**<br>***Derivation data***<br>Sensitivity: 0.95 (0.94; 0.95)<br>Specificity: 0.21 (0.21; 0.22)<br>PPV: 30%<br>NPV: 91%<br><br>***External test data***<br>Sensitivity: 0.90 (0.89; 0.91)<br>Specificity: 0.34 (0.32; 0.35)<br>PPV: 37%<br>NPV: 89%<br><br>**Suicide**<br>***Derivation data***<br>Sensitivity: 0.91 (0.81; 0.97)<br>Specificity: 0.15 (0.15; 0.16)<br>PPV: 0.4%<br>NPV: 99.8%<br><br>***External test data***<br>Sensitivity: 0.88 (0.70; 0.98)<br>Specificity: 0.24 (0.23; 0.25)<br>PPV: 0.5%<br>NPV: 99.6%	

The table continues on the next page

Table 12 continued

First author<br>Year<br>Country<br>Reference	Design<br>Setting<br>Population<br>Evaluation time	Index test<br>Reference test<br>Drop-out rate	Results<br>(sensitivity, specificity, PPV, NPV)	Comments<br>Risk of bias
**SAD PERSONS Scale**				
Bolton et al<br>2012<br>Canada<br>[19]	**Design**<br>Prospective cohort<br>Consecutive sample<br><br>**Setting**<br>Psychiatric services at ED<br>2 tertiary care hospitals<br><br>**Population**<br>Adults<br>N=4 019 presentations<br><br>**Evaluation time**<br>6 months<br><br>**Study period**<br>January 1999 to December 2010	**Index test**<br>SAD PERSONS Scale<br>n=2 846<br>Female: NR<br>Mean age: NR<br><br>**Reference test**<br>Suicide attempts<br>n=87 individuals,<br>80 completed<br>SAD PERSONS Scale<br>Female: 52.9%<br>Mean age: NR<br><br>**Drop-out rate**<br>Unclear	**Cut-off High vs moderate + low**<br>Sensitivity: 0.13 (0.06; 0.22)<br>Specificity: 0.94 (0.93; 0.94)<br>PPV: 5.3%<br>NPV: 97.4%	**Sampling method**<br>Consecutive sample<br><br>**Blinding**<br>NR<br><br>**Handling of missing data**<br>Removed from the analysis (15–19%)<br><br>**Other comments**<br>No investigation of completed suicides. Drop-out rate uncertain<br><br>**Risk of bias**<br>Moderate
Saunders et al<br>2013<br>United Kingdom<br>[20]	**Design**<br>Cohort<br><br>**Setting**<br>Self harm service at major general hospital ED<br><br>**Population**<br>Self harm, N=126<br><br>**Participation rate**<br>100% of referrals, approximately 60% of self-harmers presenting to ED<br><br>**Evaluation time**<br>6 months<br><br>**Study period**<br>June 2011 to August 2011	**Index test**<br>SAD PERSONS SCALE<br>n=126<br>Female: 57.1%<br>Mean age: 33±14.5 years<br><br>**Reference test**<br>Self-harm<br>6 months: n=31<br>Female: 61%<br>Mean age: NR<br><br>**Drop-out rate**<br>0	**Cut-off ≥7 (high risk)**<br>Sensitivity: 0.06 (0.01; 0.21)<br>Specificity: 0.97 (0.91; 0.99)<br>PPV: 40%<br>NPV: 76%	**Other comments**<br>Only self-harm patients referred to specialist self-harm services were included, and these persons might have had higher scores than those seen in the ED as a whole

The table continues on the next page

Table 12 continued

First author<br>Year<br>Country<br>Reference	Design<br>Setting<br>Population<br>Evaluation time	Index test<br>Reference test<br>Drop-out rate	Results<br>(sensitivity, specificity, PPV, NPV)	Comments<br>Risk of bias
**Modified SAD PERSONS Scale**				
Bolton et al<br>2012<br>Canada<br>[19]	**Design**<br>Prospective cohort<br><br>**Setting**<br>Psychiatric services at ED<br>two tertiary care hospitals<br><br>**Population**<br>Adults<br>N=4 019 presentations<br><br>**Evaluation time**<br>6 months<br><br>**Study period**<br>January 2009 to December 2010	**Index test**<br>Modified SAD PERSONS Scale<br>n=2 713<br>Female: NR<br>Mean age: NR<br><br>**Reference test**<br>Suicide attempts<br>n=87 individuals,<br>76 completed modified<br>SAD PERSONS Scale<br>Female: NR<br>Mean age: NR<br><br>**Drop-out rate**<br>Uncertain	**Cut-off High vs moderate and low**<br>Sensitivity: 0.29 (0.19; 0.40)<br>Specificity: 0.89 (0.88; 0.90)<br>PPV: 7.4%<br>NPV: 97.7%	**Sampling method**<br>Consecutive sample<br><br>**Blinding**<br>NR<br><br>**Handling of missing data**<br>Removed from the analysis (15–19%)<br><br>**Other comments**<br>No investigation of completed suicides.<br>Drop-out rate uncertain
**SIS**				
Harriss et al<br>2005<br>UK<br>[12]	**Design**<br>Cohort<br><br>**Setting**<br>ED, general hospital<br><br>**Population**<br>N=4 156 patients self-harm<br>N=6 316 episodes of deliberate self-harm<br><br>**Participation rate**<br>NR<br><br>**Evaluation time**<br>3–7 years<br><br>**Study period**<br>January 1993 to December 1997	**Index test**<br>SIS<br>n= 2 719 patients,<br>n=3 339 episodes<br>Female: 47.4%<br>Mean age: NR<br><br>**Reference test**<br>Suicide<br>n=54<br>Female: 44.4%<br>Mean age: NR<br><br>**Drop-out rate**<br>230 (11%)	**Cut-off 10**<br>Sensitivity: 0.76 (0.62; 0.87)<br>Specificity: 0.49 (0.47; 0.51)<br>PPV: 4.2%<br>NPV: 99%<br><br>**Cut-off 14**<br>Sensitivity: 0.67 (0.53; 0.79)<br>Specificity: 0.75 (0.74; 0.77)<br>PPV: 4%<br>NPV: 99%	**Handling of missing data**<br>NR

The table continues on the next page

Table 12 continued

First author<br>Year<br>Country<br>Reference	Design<br>Setting<br>Population<br>Evaluation time	Index test<br>Reference test<br>Drop-out rate	Results<br>(sensitivity, specificity, PPV, NPV)	Comments<br>Risk of bias
Niméus et al<br>2002<br>Sweden<br>[13]	**Design**<br>Cohort<br><br>**Setting**<br>Medical ICU<br><br>**Population**<br>Suicide attempters<br>N=674<br><br>**Participation rate**<br>555/674<br><br>**Evaluation time**<br>Mean 4.5 years<br>(range 10 months to 8.8 years)<br><br>**Study period**<br>NR	**Index test**<br>SIS<br>n=555<br>Female: 62.7%<br>Mean age: 38.8±16.1 years<br>Violent suicide attempts:<br>26 (4.7%)<br>Repeaters: 246 (44.7%)<br><br>**Reference test**<br>Suicide<br>n=22<br>Female: 50%<br>Mean age: 54.8 years<br><br>**Drop-out rate**<br>0	**Cut-off 19**<br>Sensitivity: 0.59 (0.36; 0.79)<br>Specificity: 0.77 (0.74; 0.81)<br>PPV: 9.7%<br>NPV: 98%	**Other comments**<br>Suicides determined by local dept of forensic medicine and national bureau of statistics (high data quality)
**SoS-4**				
Bilén et al<br>2013<br>Sweden<br>[25]	**Design**<br>Cohort<br><br>**Setting**<br>2 large ED<br><br>**Population**<br>Adults, DSH<br>N=328<br><br>**Evaluation time**<br>6 months<br><br>**Study period**<br>Mars 2011 to June 2011	**Index test**<br>SoS-4<br>n=325<br>Female: 70%<br>Mean age: NR<br><br>**Reference test**<br>Repeated DSH/<br>suicide attempts<br>n=80<br>Female: NR<br>Mean age: NR<br><br>**Drop-out rate**<br>3/328 (1%)	**Cut-off 0.14**<br>Sensitivity: 0.89 (0.80; 0.95)<br>Specificity: 0.11 (0.07; 0.16)<br>PPV: 25%<br>NPV: 98%	**Sampling method**<br>Consecutive sample

The table continues on the next page

Table 12 continued

First author<br>Year<br>Country<br>Reference	Design<br>Setting<br>Population<br>Evaluation time	Index test<br>Reference test<br>Drop-out rate	Results<br>(sensitivity, specificity, PPV, NPV)	Comments<br>Risk of bias
Bilén et al<br>2013<br>Sweden<br>[26]	**Design**<br>Cohort<br><br>**Setting**<br>ED<br><br>**Population**<br>N= 1 524<br>Adults, DSH<br>History of DSH: 35%<br>Treated with antidepressants: 57%<br><br>**Participation rate**<br>NR<br><br>**Evaluation time**<br>6 months<br><br>**Study period**<br>January 2003 to December 2005	**Index test**<br>SoS-4<br>n=1 524<br>Female: 65%<br>Mean age: NR<br><br>**Reference test**<br>Suicide + repeated DSH<br>n=309<br>Female: 70%<br>Mean age: 39.5 years<br>(range 3318–91 years)<br><br>Suicide<br>n=12<br>Repeated DSH<br>n=297<br><br>**Drop-out rate**<br>0%	**Cut-off 0.14**<br>Repeated DSH<br>Sensitivity: 0.90 (0.86; 0.93)<br>Specificity: 0.18 (0.16; 0.20)<br>PPV: 22%<br>NPV: 88%	**Sampling method**<br>Consecutive sample
**SPS**				
Larzelere et al<br>1996<br>USA<br>[21]	**Design**<br>Cohort<br><br>**Setting**<br>Group home<br><br>**Population**<br>N=840<br>Adolescents<br><br>**Evaluation time**<br>Mean 15.5 months<br>(follow-up during group home treatment only, range 4–41.8 months)<br><br>**Study period**<br>1988 to March 1993	**Index test**<br>SPS<br>n=840 patients<br>Female: 34%<br><br>**Reference test**<br>Suicide attempts during group home stay<br>n=29<br><br>**Drop-out rate**<br>6/840 (0.7%)	**Cut-off ≥78**<br>Sensitivity: 48.3%<br>Specificity: 80.3%<br>PPV: 8.8%	**Sampling method**<br>Sample enriched with 11 self-harmers

The table continues on the next page

Table 12 continued

First author<br>Year<br>Country<br>Reference	Design<br>Setting<br>Population<br>Evaluation time	Index test<br>Reference test<br>Drop-out rate	Results<br>(sensitivity, specificity, PPV, NPV)	Comments<br>Risk of bias
**SSI-C**				
Beck et al<br>1999<br>USA<br>[7]	**Design**<br>Cohort<br><br>**Setting**<br>Center of cognitive therapy<br><br>**Population**<br>N=3 701, outpatients who were evaluated at intake, 65% history of suicide attempts<br><br>**Evaluation time**<br>1979–1994<br><br>**Study period**<br>1975–1994	**Index test**<br>SSI-C<br>n=3 701<br>Female: 56.7%<br>Mean age: NR<br><br>**Reference test**<br>Suicide<br>n=30<br>Female: 40%<br>Mean age: 41.1±13.68 years<br><br>**Drop-out rate**<br>128 (3%)	Cut-off derived from ROC analysis<br>**Cut-off for higher risk ≥2**<br>Sensitivity: 0.53 (0.34; 0.72)<br>Specificity: 0.83 (0.82; 0.84)<br>PPV: 2.4%<br>NpV: 99.5%	**Sampling method**<br>Few inpatients in the sample
**SSI-W**				
Beck et al<br>1999<br>USA<br>[7]	**Design**<br>Cohort<br><br>**Setting**<br>Center of cognitive therapy<br><br>**Population**<br>N=3 701, outpatients who were evaluated at intake, 65% history of suicide attempts<br><br>**Evaluation time**<br>1979–1994<br><br>**Study period**<br>1975–1994	**Index test**<br>SSI-W<br>n=3 701<br>Female: 56.7%<br>Mean age: NR<br><br>**Reference test**<br>Suicide<br>n=30<br>Female: 40%<br>Mean age: 41.1±13.68 years<br><br>**Drop-out rate**<br>128 (3%)	Cut-off derived from ROC analysis<br>**Cut-off for higher risk ≥16**<br>Sensitivity: 0.80 (0.61; 0.92)<br>Specificity: 0.78 (0.77; 0.79)<br>PPV: 2.8%<br>NPV: 99.8%	**Sampling method**<br>Few patients in the sample

The table continues on the next page

Table 12 continued

First author<br>Year<br>Country<br>Reference	Design<br>Setting<br>Population<br>Evaluation time	Index test<br>Reference test<br>Drop-out rate	Results<br>(sensitivity, specificity, PPV, NPV)	Comments<br>Risk of bias
**SUAS**				
Waern et al<br>2010<br>Sweden<br>[22]	**Design**<br>Prospective cohort<br><br>**Setting**<br>Emergency ward general hospital<br><br>**Population**<br>Adults (18–68 years) with suicide attempts<br>N=206 fulfilled study criteria and 165 accepted participation; 162 of these completed the SUAS interview (79% of those who met study criteria)<br><br>**Evaluation time**<br>3 years<br><br>**Study period**<br>NR	**Index test**<br>SUAS at baseline<br>n=162<br>Female: NR<br>Mean age: NR<br><br>**Reference test**<br>Repeat suicidal behaviour (fatal or non-fatal)<br>n=61<br>Female: NR<br>Mean age: NR<br><br>**Drop-out rate**<br>0	**Cut-off ≥24**<br>Sensitivity<br>BL: 61%<br>Specificity<br>BL: 60%	**Sampling method**<br>Unclear patient flow<br><br>**Other comments**<br>"High SUAS" (score above 30) was defined after the fact, after perusal of data. It is preferable to show sensitivity and specificity as determined for the optimal cut off by the ROC analysis (24)

AUC = Area under curve; **BDI** = Beck depression inventory; **BHS** = Beck's hoplessness scale; **BL** = baseline; **C-SSRS** = Colombia-suicide severity rating scale; **DSH** = Deliberate self-harm; **DSP** = Deliberate self-poisoning; **E codes** = External causes of injury codes; **ED** = Emergency department; **ERRS** = Edinburgh risk of repetition scale; **IAT** = Implicit association test; **ICU** = Intensive care unit; **MINI** = International neuropsychiatric interview; **MSHR** = Manchester self harm rule; **N** = number; **NPV** = Negative predictive value; **NR** = not reported; **NSSI** = Non-suicidal self-injury; **PHQ-9** = Patient health questionnaire-9; **PPV** = Positive predictive value; **ReACT** = Recent self-harm in the past year – alone or homeless, cutting used as a method of harm, treatment for at psychiatric disorder; **ROC** = Receiver operating characteristics; **SAD** = Acronym (sex, age, depression); **SIS** = Suicide intent scale; **SoS-4** = Södersjukhuset self-harm rule; **SPS** = Suicide probability scale; **SSI-C** = Scale for suicide ideation-Current; **SSI-W**= Scale for suicide ideation-Worst; **SUAS** = Suicide assessment scale

13. **Ordförklaringar och förkortningar**

**AUC (area under the curve)**	Ett statistiskt begrepp för summering av data från ett antal mätningar på en individ
**Bias**	Systematiskt fel
**Cut-off**	Tröskelvärde. Vilken sensitivitet (se sensitivitet) respektive specificitet (se specificitet) ett test har beror på var man sätter ”cut-off”, alltså gränsen mellan positivt och negativt
**DSM (Diagnostic and Statistical Manual of Mental Disorders)**	DSM-manualen används över hela världen för att diagnostisera psykiska sjukdomar och utvecklingsrelaterade tillstånd
**Heterogenitet**	Olikheter, variationer eller skillnader
**ICD (International Classification of Diseases)**	WHO:s internationella sjukdomsklassifikation
**KI (Konfidensintervall)**	Ett talintervall som med viss angiven sannolikhet innefattar det sanna värdet av till exempel ett medeltal eller en oddskvot
**Kognitiv psykoterapi**	Metod att tankemässigt få patienten att styra sina känslor och reagera rationellt
**Kompositmått**	Sammanräkning av flera olika effektmått för att uppnå högre statistisk styrka i en studie

**Lex Maria**	Regel inom hälso- och sjukvården som innebär att vårdgivaren ska anmäla händelser som har medfört eller hade kunnat medföra en allvarlig vårdskada till Inspektionen för vård och omsorg (IVO)
**Likerttyp**	Skala som utformats av den amerikanske psykologen Rensis Likert och som används i frågeformulär för att mäta olika attityder hos den svarande
**Neurokognitiva test**	Test som används för att fastställa störningar i de områden och funktioner i centrala nervsystemet som återspeglas i förändringar och försvagningar i människors sätt att uppfatta, lära, minnas och tänka. Hit räknas funktioner som koncentration och uppmärksamhet
**Nominalskala**	Kategoriseringsskala med vars hjälp man ordnar en viss typ av företeelser i delgrupper: människor kan indelas i män, kvinnor och barn, i vita och färgade, i österlänningar och västerlänningar
**NPV (Negativt prediktivt värde)**	Talar om hur pass bra ett test är på att förutsäga hälsa/att patienten är frisk
**Ordinalskala**	Mellanform mellan kvalitativ kategorisering och rent kvantitativ mätning. Eftersom rangordningen kan bygga på så kvalitativa egenskaper som social ställning (vid rangordning av människor i socio-ekonomiska grupper), räknas ofta variabler som uttrycks i ordinalskala som kvalitativa (ibland som semikvantitativa)
**PPV**	Positivt prediktivt värde
**Prediktion**	Förutsägelse, ofta grundad på ingående kännedom om de faktorer som är av betydelse i sammanhanget

**Prospektiv studie**	Studie som går framåt i tiden där man börjar samla in data om deltagarna vid tidpunkten för undersökningen
**Psykometri**	Läran om psykologisk mätning. Att mäta tankar, känslor och beteende
**ROC (receiver operating characteristic)**	För att hitta en optimal cut-off (se cut-off) måste man ta hänsyn till både hur farlig en sjukdom är (och om en hög sensitivitet eller specificitet är mest angelägen) men också hur vanlig en sjukdom är ("prevalensen"). Ofta används så kallade ROC-kurvor för att sätta en bra cut-off
**Sensitivitet**	Andelen sjuka som identifieras med ett test För att inte missa en sjukdom bör man välja ett test med hög sensitivitet
**Somatisk vård**	Vård för kroppslig ohälsa
**Specitivitet**	Andelen friska som friskförklaras med ett test För att vara helt säker på ett en person verkligen lider av en sjukdom (t ex innan start av en livshotande behandling), är specificiteten det viktigaste
**Triagering**	Process för att sortera och prioritera patienter med utgångspunkt från sjukdomshistoria, symtom och i akut somatisk vård vitalparametrar såsom andningsfrekvens, puls och kroppstemperatur, i syftet att de mest allvarliga eller brådskande fallen ska behandlas först. Ordet kommer från franskans trier, sortera

14. **Personer som medverkat till denna rapport**

Projektgrupp

Bo Runeson (ordförande)
Professor, överläkare, Karolinska Institutet, Stockholm

Ingalill Jildevik Adamsson
Överläkare, Ungdomspsykiatriska mottagningen (UPM), Östersund

Tobias Edbom
Med dr, leg sjuksköterska, BUP/Karolinska Institutet, Stockholm

Anna Lindblad
Leg läkare, Institutionen för lärande, informatik, management och etik (LIME), Karolinska Institutet

Margda Waern
Professor, överläkare, Göteborgs universitet, Göteborg

Kansli

Elisabeth Gustafsson
Projektadministratör, SBU, Stockholm

Harald Gyllensvärd
Hälsoekonom, SBU, Stockholm

Jenny Odeberg
Projektledare, med dr, SBU, Stockholm

Hanna Olofsson
Informationsspecialist, SBU, Stockholm

Agneta Pettersson
Biträdande projektledare, SBU, Stockholm

Externa granskare

SBU anlitar externa granskare av sina rapporter. Dessa har kommit med värdefulla kommentarer, som i hög grad bidragit till att förbättra rapporten. I slutversionen av rapporten har SBU dock inte kunnat tillgodose alla ändrings- eller tilläggsförslag från de externa granskarna, bland annat därför att de inte alltid varit samstämmiga. De externa granskarna står därför inte nödvändigtvis bakom samtliga slutsatser eller andra texter i rapporten.

Externa granskare har varit:

Lil Träskman-Bendz
Professor emerita, Avdelningen för psykiatri, Medicinska fakulteten, Lunds Universitet

Øivind Ekeberg
Professor emeritus, Akutmedisinsk avedeling, Oslo Universitetssykehus Ullevål, Norge

Sandra af Winklerfelt Hammarberg
Leg läkare, APC, Karolinska Institutet, Stockholm

Referensgrupp

Vi har under projektets gång använt en referensgrupp för att stämma av och få synpunkter på etiska aspekter på området. Referensgruppen[1] har bestått av:

Jenny Molin
Leg psykiatrisjuksköterska, doktorand,
Psykiatriska riksföreningen för sjuksköterskor

Ullakarin Nyberg
Med dr, överläkare, Norra Stockholms Psykiatri

Bengt Sprowede
Ambassadör, Föreningen Hjärnkoll

Palle Storm
Ambassadör, Föreningen Hjärnkoll

Bindningar och jäv

Sakkunniga och granskare har i enlighet med SBU:s krav inlämnat deklaration rörande bindningar och jäv. Dessa dokument finns tillgängliga på SBU:s kansli. SBU har bedömt att de förhållanden som redovisas där är förenliga med kraven på saklighet och opartiskhet.

---

1 Representant för NSPH var inbjuden men förhindrad att delta.

15. Referenser

1. Ahmedani BK, Simon GE, Stewart C, Beck A, Waitzfelder BE, Rossom R, et al. Health care contacts in the year before suicide death. J Gen Intern Med 2014;29:870-7.

2. Tidemalm D, Langstrom N, Lichtenstein P, Runeson B. Risk of suicide after suicide attempt according to coexisting psychiatric disorder: Swedish cohort study with long term follow-up. BMJ 2008;337:a2205.

3. Reutfors J, Brandt L, Ekbom A, Isacsson G, Sparen P, Osby U. Suicide and hospitalization for mental disorders in Sweden: a population-based case-control study. J Psychiatr Res 2010;44:741-7.

4. SBU. Utvärdering av metoder i hälso- och sjukvården – En handbok. Tillgänglig från www.sbu.se/metodbok. ISBN 978-91-85413-55-3 (tryck). ISBN 978-91-85413-56-0 (PDF).

5. Simon GE, Rutter CM, Peterson D, Oliver M, Whiteside U, Operskalski B, et al. Does response on the PHQ-9 Depression Questionnaire predict subsequent suicide attempt or suicide death? Psychiatr Serv 2013; 64: 1195-202.

6. Beck AT, Brown G, Berchick RJ, Stewart BL, Steer RA. Relationship between hopelessness and ultimate suicide: a replication with psychiatric outpatients. Am J Psychiatry 1990;147:190-5.

7. Beck AT, Brown GK, Steer RA, Dahlsgaard KK, Grisham JR. Suicide ideation at its worst point: a predictor of eventual suicide in psychiatric outpatients. Suicide Life Threat Behav 1999;29:1-9.

8. Keller F, Wolfersdorf M. Hopelessness and the tendency to commit suicide in the course of depressive disorders. Crisis 1993;14:173-7.

9. Nimeus A, Traskman-Bendz L, Alsen M. Hopelessness and suicidal behavior. J Affect Disord 1997;42:137-44.

10. Klonsky D, Kotov R, Bakst S, Rabinowitz J, Bromet EJ. Hopelessness as a predictor of attempted suicide among first admission patients with psychosis: a 10-year cohort study. Suicide Life Threat Behav 2012;42:1-10.

11. Posner K, Brown GK, Stanley B, Brent DA, Yershova KV, Oquendo MA, et al. The Columbia-Suicide Severity Rating Scale: initial validity and internal consistency findings from three multisite studies with adolescents and adults. Am J Psychiatry 2011;168:1266-77.

12. Harriss L, Hawton K. Suicidal intent in deliberate self-harm and the risk of

suicide: the predictive power of the Suicide Intent Scale. J Affect Disord 2005;86:225-33.

13. Niméus A, Alsén M, Träskman-Bendz L. High suicidal intent scores indicate future suicide. Archives of Suicide Research 2002;6:211-19.

14. Steeg S, Kapur N, Webb R, Applegate E, Stewart SLK, Hawton K, et al. The development of a population-level clinical screening tool for self-harm repetition and suicide: The ReACT Self-Harm Rule. Psychol Med 2012;42:2383-94.

15. Sidley GL, Calam R, Wells A, Hughes T, Whitaker K. The prediction of parasuicide repetition in a high-risk group. Br J Clin Psychol 1999;38 (Pt 4):375-86.

16. Carter GL, Clover KA, Bryant JL, Whyte IM. Can the Edinburgh Risk of Repetition Scale predict repetition of deliberate self-poisoning in an Australian clinical setting? Suicide Life Threat Behav 2002;32:230-9.

17. Randall JR, Rowe BH, Dong KA, Nock MK, Colman I. Assessment of self-harm risk using implicit thoughts. Psychol Assess 2013;25:714-21.

18. Roaldset JO, Linaker OM, Bjorkly S. Predictive validity of the MINI suicidal scale for self-harm in acute psychiatry: a prospective study of the first year after discharge. Arch Suicide Res 2012; 16:287-302.

19. Bolton JM, Spiwak R, Sareen J. Predicting suicide attempts with the SAD PERSONS scale: a longitudinal analysis. J Clin Psychiatry 2012;73: e735-41.

20. Saunders K, Brand F, Lascelles K, Hawton K. The sad truth about the SADPERSONS Scale: an evaluation of its clinical utility in self-harm patients. Emerg Med J 2013;0:1-3.

21. Larzelere RE, Smith GL, Batenhorst LM, Kelly DB. Predictive validity of the suicide probability scale among adolescents in group home treatment. J Am Acad Child Adolesc Psychiatry 1996;35:166-72; discussion 172.

22. Waern M, Sjostrom N, Marlow T, Hetta J. Does the suicide assessment scale predict risk of repetition? A prospective study of suicide attempters at a hospital emergency department. Eur Psychiatry 2010;25:421-6.

23. Cooper J, Kapur N, Dunning J, Guthrie E, Appleby L, Mackway-Jones K. A clinical tool for assessing risk after self-harm. Ann Emerg Med 2006;48:459-66.

24. Randall JR, Rowe BH, Colman I. Emergency department assessment of self-harm risk using psychometric questionnaires. Can J Psychiatry 2012;57:21-8.

25. Bilen K, Ponzer S, Ottosson C, Castren M, Owe-Larsson B, Ekdahl K, et al. Can repetition of deliberate self-harm be predicted? A prospective multicenter study validating clinical decision rules. J Affect Disord 2013;149:253-8.

26. Bilen K, Ponzer S, Ottosson C, Castren M, Pettersson H. Deliberate self-harm patients in the emergency department:

who will repeat and who will not? Validation and development of clinical decision rules. Emerg Med J 2013;30:650-6.

27. O'Mara RM, Hill RM, Cunningham RM, King CA. Adolescent and parent attitudes toward screening for suicide risk and mental health problems in the pediatric emergency department. Pediatr Emerg Care 2012;28:626-32.

28. Hermerén G. Kunskapens pris (The cost of knowledge). Stockholm: HSFR; 1986.

29. Psykiatri stöd. Stockholms läns landsting. Tillgänglig från http://www1.psykiatristod.se/Psykiatristod/Psykiatriprogram/Suicidnara-patienter/. Nedladdad 2015-03-10.

30. Bernert RA, Roberts LW. Ethics Commentary: Suicide Risk: Ethical Considerations in the Assessment and Management of Suicide Risk. FOCUS 2012;10:467-72.

31. Lang M, Uttaro T, Caine E, Carpinello S, Felton C. Implementing routine suicide risk screening for psychiatric outpatients with serious mental disorders: I. Qualitative results. Arch Suicide Res 2009;13:160-8.

32. Ballard ED, Bosk A, Snyder D, Pao M, Bridge JA, Wharff EA, et al. Patients' opinions about suicide screening in a pediatric emergency department. Pediatr Emerg Care 2012;28:34-8.

33. Dazzi T, Gribble R, Wessely S, Fear NT. Does asking about suicide and related behaviours induce suicidal ideation? What is the evidence? Psychol Med 2014;44:3361-3.

34. Runeson B. Stockholms Läns Landsting. Regionalt vårdprogram. Suicidnära patienter 2010. ISBN 91-85211-72-9. RV 2010:02. Tillgänglig från www1.psykiatristod.se/Global/vardprogram_fulltext/RV_Suicidnara_patienter_2010.pdf. Nedladdad 2015-03-13.

35. Lynöe N, Juth N. Medicinska etikens ABZ. Liber AB, 2009. ISBN 978-91-47-09413-4. s 332.

36. Kaiser. N, Waern. M, Salander RE. Psychiatrists' experiences of suicide risk assessment: what do they actually do and what are the challenges? In: Proceedings of the 47th conference of the Am Assoc Suicidology Am Assoc Suicidology. Los Angeles, April 2014

37. Steingrimsson. S, Gonzales A, Waern M, Dellepian M, Joannou M. Attitudes to suicide risk assessment among young physicians. 14th European Symposium on Suicidal Behavior, Tallinn 2014.

38. Lönnqvist J. Major psychiatric disorders in suicide and suicide attempters. In: Wasserman D, Wasserman C, editors. Oxford textbook of suicidology and suicide prevention: a global perspective. Oxford: Oxford University Press; 2009. p 281-2.

39. Vuorilehto M, Valtonen HM, Melartin T, Sokero P, Suominen K, Isometsa ET. Method of assessment determines prevalence of suicidal ideation among patients with depression. Eur Psychiatry 2014;29:338-44.

40. Cassells C, Paterson B, Dowding D, Morrison R. Long- and short-term risk

factors in the prediction of inpatient suicide: review of the literature. Crisis 2005;26:53-63.

41. Nock MK, Borges G, Bromet EJ, Cha CB, Kessler RC, Lee S. Suicide and suicidal behavior. Epidemiol Rev 2008;30:133-54.

42. Larkin C, Di Blasi Z, Arensman E. Risk factors for repetition of self-harm: a systematic review of prospective hospital-based studies. PLoS One 2014;9:e84282.

43. O'Connor E, Gaynes BN, Burda BU, Soh C, Whitlock EP. Screening for and treatment of suicide risk relevant to primary care: a systematic review for the U.S. Preventive Services Task Force. Ann Intern Med 2013;158:741-54.

44. Perlman C. Suicide risk assessment inventory: A resource guide for Canadian health care organisations. Ontario Hospital Association and Canadian Patient Safety Institute. ISBN 978-0-88621-335-0. Publication Number: 332. 2011.

45. Warden S, Spiwak R, Sareen J, Bolton JM. The SAD PERSONS scale for suicide risk assessment: a systematic review. Arch Suicide Res 2014;18:313-26.

46. Suicidnära patienter. Kliniska riktlinjer för utredning och vård. svensk psykiatri, nr 12, Svenska psykiatriska föreningen och Gothia fortbildning; 2013.

47. NICE. National Institute for Health and Care Excellence. Tillgänglig från www.nice.org.uk. Nedladdad 2015-03-13.

48. Cochrane-Brink KA, Lofchy JS, Sakinofsky I. Clinical rating scales in suicide risk assessment. Gen Hosp Psychiatry 2000;22:445-51.
