# Supplementary material for: Instruments for the assessment of suicide risk: A systematic review evaluating the certainty of the evidence
Source: PLoS One. 2017 Jul 19;12(7):e0180292. doi: 10.1371/journal.pone.0180292 (PMC5517300; doi:10.1371/journal.pone.0180292)
Supplement: S2 File — (DOCX) [file pone.0180292.s002.docx]

**S2 File. Explanation of the submission and its relation to the Swedish report**

The report (S1 File) is in the Swedish language. It is aimed at clinicians, not a peer-reviewed publication. It targeted primarily doctors and other care professionals who meet and treat suicidal persons. Content covered other aspects including the epidemiology of suicidal behavior in Sweden, definitions of different types of suicidal behaviors, principles of testing of diagnostic scale, a description of the items and rating procedures for each scale, a section on assessment praxis in Sweden today, as well as a section on health economics and ethics.

While the introduction in the Swedish report is geared at clinicians, the introduction of this manuscript has a more academic focus, citing a somewhat different body of literature, and stressing that previous review articles have not described selection procedures in detail, and risk of bias was not considered. We make a case for the application of the GRADE procedure for the evaluation of studies that assess the predictive ability of suicide risk instruments.

The discussion section in the Swedish report was not geared to a scientifically schooled readership, as it is in the current manuscript. Additional information has been added including a discussion regarding the large number of articles that could not be included due to high degree of bias and the implications of this for future research. We suggest that more research is needed to determine a given instrument’s ability to generate conceptually valid and consistent data. The manuscript should thus not be regarded as a dual publication.
