# Supplementary material for: Instruments for the assessment of suicide risk: A systematic review evaluating the certainty of the evidence
Source: PLoS One. 2017 Jul 19;12(7):e0180292. doi: 10.1371/journal.pone.0180292 (PMC5517300; doi:10.1371/journal.pone.0180292)
Supplement: S3 File — (PDF) [file pone.0180292.s003.pdf]

Date
April 25, 2017

The systematic litterature review report *"Instrument för bedömning av suicidrisk. En systematisk litteraturöversikt". Stockholm: Statens beredning för medicinsk och social utvärdering (SBU); 2015, no 242* is published by the Swedish Agency for Health Technology Assessment and Assessment of Social Services (SBU), who has the copyright to the report/review.

SBU hereby permit that the paper, based on the review, is published under the CC BY 4.0 licence in PLOS one.

Charlotte Hall
Head of department
Swedish Agency for Health Technology Assessment and Assessment of Social Services
25th of April, 2017
Stockholm Sweden

SBU Statens beredning för medicinsk och social utvärdering • www.sbu.se
Telefon 08-412 32 00 • **Fax** 08-411 32 60 • **Organisationsnummer** 202100-4417
Besöksadress S:t Eriksgatan 117, Stockholm • **Postadress** Box 6183, 102 33 Stockholm
