# Supplementary material for: Instruments for the assessment of suicide risk: A systematic review evaluating the certainty of the evidence
Source: PLoS One. 2017 Jul 19;12(7):e0180292. doi: 10.1371/journal.pone.0180292 (PMC5517300; doi:10.1371/journal.pone.0180292)
Supplement: S1 Table — (DOCX) [file pone.0180292.s005.docx]

## S 1 Table. Search strategy

## Cinahl via EBSCO 16 December 2014 - Suicide Risk Assessment Tools

| Search terms | | Items found |
| --- | --- | --- |
| Intervention: screening, test, scales | | |
|  | MM "Scales" OR MM "Psychological Tests" OR MM "Psychometrics" OR MM "Severity of Illness Indices" OR MM "Questionnaires" OR TI (Scale* OR Instrument OR Instruments OR Tool OR tools OR Measurement* OR (Clinical W0 predictor*) OR "Risk assessment" OR "risk screening" OR Questionnaire* OR inventory OR psychometric* OR "status form" Checklist* OR score*) OR ((MH "Scales" OR MH "Psychological Tests" OR MH "Psychometrics" OR MH "Severity of Illness Indices" OR MH "Questionnaires") *AND* (MH "Measurement Issues and Assessments+" OR MH "Validation Studies" OR MH "Reproducibility of Results" OR MH "Sensitivity and Specificity" OR MH "Instrument Validation" OR MH "Test-Retest Reliability" OR TI evaluat* OR TI predict*)) | 116,086 |
|  | MH "Beck Hopelessness Scale" OR MH "Hamilton Rating Scale for Depression" OR TI ("Adult Suicide Ideation Questionnaire" OR "Suicide Severity Rating scale" OR (Ducher* N1 suicidal N1 risk N0 assessment N0 scale) OR (Firestone* N0 Assessment N1 Self-Destructive N0 Thought*) OR (Geriatric N1 Scale N1 suicide N0 intent*) OR ("High-Risk Construct Scale") OR "interRAI Mental Health Severity of Self-Harm Scale" OR "Manchester Self Harm Rule" OR "Montgomery Asberg depression rating scale" OR MADRS OR "Mental Health Environment of Care Checklist" OR "Modified scale for Suicide Ideation" OR "Nurses Global Assessment of Suicide Risk" OR "Parasuicide History Inventory" OR "Paykel Suicide Items" OR PHQ-9 OR "Positive and Negative Suicide Ideation Inventory" OR PANSI OR (pictorial representation N1 illness and self-measurement N1 suicid*) OR PRISM-S OR "Quiz on Depression and Suicide in Late Life" OR "Reasons for Living Inventory" OR "risk-rescue" OR "SAD PERSONS scale" OR "Scale for Suicide Ideation" OR "Schizophrenia Suicide Risk Scale" OR "Self-Inflicted Injury Severity form" OR "Self-Injury Implicit Association Test" OR "Self-Monitoring Suicide Ideation Scale" OR "Stroop" OR "Suicidal Behaviour Questionnaire" OR "Suicide Behavior Interview" OR "suicide Implicit Association Test" OR "Suicide Intent Scale" OR "Suicide opinion Questionnaire" OR "Suicide Potential Lethality Scale" OR "Suicide Probability Scale" OR "Suicidal Ideation Questionnaire" OR "Suicidal Ideation Screening Questionnaire" OR "Suicidal Behaviours Questionnaire" OR (suicide W0 intent W0 scale*) OR "Suicide Intervention Response Inventory" OR "Suicide Ideation Scale" OR "Suicide Probability Scale" OR "Suicide Status Form" OR "Suicide Risk Assessment Scale" OR "Tool for the Assessment of Suicide" OR "Concise Health Risk Tracking Scale" OR "suicide assessment scale" OR "Karolinska Interpersonal Violence Scale") OR TI ((("symptom Driven Diagnostic System" W2 Suicide N0 Item*)) OR ((“Scale for Impact of Suicidality”) W1 (“Management, assessment and Planning of care”)) OR ((Hamilton*) W2 ("rating scale" OR "depression scale" OR "depression rating")) OR ((Beck*) W2 (”Suicide Scale” OR ”Hopelessness Scale” OR ”Anxiety Inventory” OR ”suicidal ideation scale” OR ”Suicidal Intent Scale” OR ”suicide intent scale” OR ”suicide ideation” OR ”Depression Inventory”)) OR (Beck* W0 scale* W5 suicid*)) | 1,897 |
|  | 1 OR 2 | 117,433 |
| Outcome: Suicide, Self-harm | | |
|  | MM "Suicide" OR MM "Suicide, Attempted" OR MM "Suicidal Ideation" OR TI (suicid* OR parasuicid* OR self-harm*) | 9,206 |
| Combined sets | | |
|  | 3 AND 4 | 571 |

The search result, usually found at the end of the documentation, forms the list of abstracts.

AB = Abstract

AU = Author

DE = Term from the thesaurus

MH = Term from the “Cinahl Headings” thesaurus

MM = Major Concept

TI = Title

TX = All Text. Performs a keyword search of all the  database's searchable fields

ZC = Methodology Index

* = Truncation

“ “ = Citation Marks; searches for an exact phrase

Cochrane Library via Wiley 16 December 2014 (CDSR, DARE & CENTRAL, EED)

Suicide Risk Asessment Tools

| Search terms | | Items found |
| --- | --- | --- |
| Intervention: screening, test, scales | | |
|  | Mass Screening[MeSH:NoExp] OR "Questionnaires"[MeSH] OR "Risk Assessment"[MeSH Terms] OR "Psychiatric Status Rating Scales"[Mesh] OR "Psychological Tests"[Mesh] OR "Psychometrics"[Mesh] OR (Scale* OR Instrument OR Instruments OR Tool OR tools OR Measurement* OR Clinical predictor* OR Risk assessment OR risk screening OR Questionnaire* OR inventory OR psychometric* OR status form OR Checklist* OR score*):ti | 53883 |
|  | ("Adult Suicide Ideation Questionnaire" OR "Columbia-Suicide Severity Rating Scale" OR "Ducher's suicidal risk assessment scale" OR "Firestones Assessment of Self-Destructive Thoughts" OR "Geriatric Scale for suicide intent" OR "High-Risk Construct Scale" OR "interRAI Mental Health Severity of Self-Harm Scale" OR "Manchester Self Harm Rule" OR "Montgomery Asberg depression rating scale" OR "Mental Health Environment of Care Checklist" OR "Modified scale for Suicide Ideation" OR "Nurses Global Assessment of Suicide Risk" OR "Parasuicide History Inventory" OR "Paykel Suicide Items" OR "PHQ-9" OR "Positive and Negative Suicide Ideation Inventory" OR "PRISM-S" OR "Quiz on Depression and Suicide in Late Life" OR "Reasons for Living Inventory" OR "risk-rescue" OR "SAD PERSONS scale" OR "Scale for Suicide Ideation" OR "Schizophrenia Suicide Risk Scale" OR "Self-Inflicted Injury Severity form" OR "Self-Injury Implicit Association Test" OR "Self-Monitoring Suicide Ideation Scale" OR "Stroop test" OR "Suicidal Behaviour Questionnaire" OR "Suicide Behavior Interview" OR "suicide Implicit Association Test" OR "Suicide Intent Scale" OR "suicide opinion Questionnaire" OR "Suicide Potential Lethality Scale" OR "Suicide Probability Scale" OR "Suicidal Ideation Questionnaire" OR "Suicidal Ideation Screening Questionnaire" OR "Suicidal Behaviours Questionnaire" OR "suicide intent scale" OR "Suicide Intervention Response Inventory" OR "Suicide Ideation Scale" OR "Suicide Probability Scale" OR "Suicide Status Form" OR "Suicide Risk Assessment Scale" OR "suicide assessment scale" OR "Tool for the Assessment of Suicide Risk" OR "symptom Driven Diagnostic System" OR "Scale for Impact of Suicidality" OR (Hamilton* AND ("rating scale" OR "depression scale")) OR ((Beck OR Beck') AND ("Suicide Scale" OR "Hopelessness Scale" OR "Anxiety Inventory" OR "suicidal ideation scale" OR "Suicidal Intent Scale" OR "Depression Inventory"))):ti, ab, kw | 5629 |
|  | 1 OR 2 | 56999 |
| Outcome: Suicide, Self-harm | | |
|  | "Suicide"[Mesh:NoExp] OR "Suicidal Ideation"[Mesh] OR "Suicide, Attempted"[Mesh] OR "Self-Injurious Behavior"[Mesh:NoExp] OR (suicid* OR parasuicid* OR self-harm*):ti | 1172 |
| Combined sets | | |
|  | 3 AND 4 | CDSR/0DARE/11Central/326HTA/10EED/3 |

The search result, usually found at the end of the documentation, forms the list of abstracts.

[AU] = Author

[MAJR] = MeSH Major Topic

[MeSH] = Term from the Medline controlled vocabulary, including terms found below this term in the MeSH hierarchy

[MeSH:NoExp] = Does not include terms found below this term in the MeSH hierarchy

Systematic[SB] = Filter for retrieving systematic reviews

[TI] = Title

[TIAB] = Title or abstract

[TW] = Text Word

* = Truncation

“ “ = Citation Marks; searches for an exact phrase

CDSR = Cochrane Database of Systematic Review

CENTRAL = Cochrane Central Register of Controlled Trials, “trials”

CRM = Method Studies

DARE = Database Abstracts of Reviews of Effects, “other reviews”

EED = Economic Evaluations

HTA = Health Technology Assessments

Embase via Elsevier 16 December 2014 - Suicide Risk Assessment Tools

| Search terms | | Items found |
| --- | --- | --- |
| Intervention: screening, test, scales | | |
|  | 'psychological rating scale'/mj OR 'questionnaire'/mj OR 'rating scale'/mj OR 'psychologic test'/mj OR 'psychometry'/mj OR (('sensitivity and specificity'/de OR 'validity'/exp OR'reproducibility'/exp OR 'receiver operating characteristic'/de OR 'probability'/de) AND ('psychological rating scale'/de OR 'questionnaire'/de OR 'rating scale'/de OR 'psychologic test'/de OR 'psychometry'/de)) | 73,998 |
|  | 'named inventories, questionnaires and rating scales'/de OR 'Beck Depression Inventory'/mj OR 'Beck Hopelessness Scale'/mj OR 'Hamilton Anxiety Scale'/mj OR 'Montgomery Asberg Depression Rating Scale'/mj OR 'Stroop test'/mj | 14,585 |
|  | ("Adult Suicide Ideation Questionnaire" OR "Suicide Severity Rating scale" OR "Firestone Assessment of Self-Destructive Thoughts" OR "High-Risk Construct Scale" OR "interRAI Mental Health Severity of Self-Harm Scale" OR "Manchester Self Harm Rule" OR "Montgomery Asberg depression rating scale" OR MADRS OR "Mental Health Environment of Care Checklist" OR "Modified scale for Suicide Ideation" OR "Nurses Global Assessment of Suicide Risk" OR "Parasuicide History Inventory" OR "Paykel Suicide Items" OR PHQ-9 OR "Positive and Negative Suicide Ideation Inventory" OR PANSI OR ("pictorial representation of illness and self-measurement" NEAR/1 suicid*) OR PRISM-S OR "Quiz on Depression and Suicide in Late Life" OR "Reasons for Living Inventory" OR "risk-rescue" OR "SAD PERSONS scale" OR "Scale for Suicide Ideation" OR "Schizophrenia Suicide Risk Scale" OR "Self-Inflicted Injury Severity form" OR "Self-Injury Implicit Association Test" OR "Self-Monitoring Suicide Ideation Scale" OR "Stroop" OR "Suicidal Behaviour Questionnaire" OR "Suicide Behavior Interview" OR "suicide Implicit Association Test" OR "Suicide Intent Scale" OR "Suicide opinion Questionnaire" OR "Suicide Potential Lethality Scale" OR "Suicide Probability Scale" OR "Suicidal Ideation Questionnaire" OR "Suicidal Ideation Screening Questionnaire" OR "Suicidal Behaviours Questionnaire" OR "suicide intent scale" OR "Suicide Intervention Response Inventory" OR "Suicide Ideation Scale" OR "Suicide Probability Scale" OR "Suicide Status Form" OR "Suicide Risk Assessment Scale" OR "Tool for the Assessment of Suicide" OR "Concise Health Risk Tracking Scale" OR "suicide assessment scale" OR "Karolinska Interpersonal Violence Scale" OR "Scale for Impact of Suicidality"):ti | 1,606 |
|  | (hamilton* NEAR/2 ('rating scale' OR 'depression scale' OR 'depression rating')):ti OR ((Beck*) NEAR/2 ("Suicide Scale" OR "Hopelessness Scale" OR "Anxiety Inventory" OR "suicidal ideation scale" OR "Suicidal Intent Scale" OR "suicide intent scale" OR "suicide ideation" OR "Depression Inventory")):ti | 657 |
|  | 1 OR 2 OR 3 OR 4 | 88,735 |
| Outcome: suicide, suicide attempts | | |
|  | 'suicide'/mj OR 'suicide attempt'/mj OR 'suicidal behavior'/exp/mj OR suicid*:ti OR parasuicid*:ti OR selfharm*:ti OR (self NEXT/0 harm*):ti | 44,855 |
| Combined sets | | |
|  | 5 AND 6 | 562 |
|  | 7 AND ([article]/lim OR [article in press]/lim) AND ([danish]/lim OR [english]/lim OR [french]/lim OR [german]/lim OR [norwegian]/lim OR [swedish]/lim) AND [1990-2014]/py | 420 |

/de= Term from the EMTREE controlled vocabulary

/exp= Includes terms found below this term in the EMTREE hierarchy

/mj = Major Topic

:ab = Abstract

:au = Author

:ti = Article Title

:ti:ab = Title or abstract

* = Truncation

“ “ = Citation Marks; searches for an exact phrase “ “ = Citation Marks; searches for an exact phrase

PsycInfo via EBSCO 16 December 2014 - Suicide Risk Assessment Tools

| Search terms | | Items found |
| --- | --- | --- |
| Intervention: screening, test, scales | | |
|  | DE "Rating Scales" OR DE "Psychometrics" OR DE "Test Reliability" OR DE "Test Validity" OR DE "Test Construction" OR DE "Screening Tests" OR DE "Symptom Checklists" OR DE "Measurement" OR TI (Scale* OR Instrument OR Instruments OR Tool OR tools OR Measurement* OR (Clinical W0 predictor*) OR "Risk assessment" OR "risk screening" OR Questionnaire* OR inventory OR psychometric* OR "status form" Checklist* OR score*) | 184,515 |
|  | TI ("Adult Suicide Ideation Questionnaire" OR "Suicide Severity Rating scale" OR (Ducher* N1 suicidal N1 risk N0 assessment N0 scale) OR (Firestone* N0 Assessment N1 Self-Destructive N0 Thought*) OR (Geriatric N1 Scale N1 suicide N0 intent*) OR ("High-Risk Construct Scale") OR "interRAI Mental Health Severity of Self-Harm Scale" OR "Manchester Self Harm Rule" OR "Montgomery Asberg depression rating scale" OR MADRS OR "Mental Health Environment of Care Checklist" OR "Modified scale for Suicide Ideation" OR "Nurses Global Assessment of Suicide Risk" OR "Parasuicide History Inventory" OR "Paykel Suicide Items" OR PHQ-9 OR "Positive and Negative Suicide Ideation Inventory" OR PANSI OR (pictorial representation N1 illness and self-measurement N1 suicid*) OR PRISM-S OR "Quiz on Depression and Suicide in Late Life" OR "Reasons for Living Inventory" OR "risk-rescue" OR "SAD PERSONS scale" OR "Scale for Suicide Ideation" OR "Schizophrenia Suicide Risk Scale" OR "Self-Inflicted Injury Severity form" OR "Self-Injury Implicit Association Test" OR "Self-Monitoring Suicide Ideation Scale" OR "Stroop" OR "Suicidal Behaviour Questionnaire" OR "Suicide Behavior Interview" OR "suicide Implicit Association Test" OR "Suicide Intent Scale" OR "Suicide opinion Questionnaire" OR "Suicide Potential Lethality Scale" OR "Suicide Probability Scale" OR "Suicidal Ideation Questionnaire" OR "Suicidal Ideation Screening Questionnaire" OR "Suicidal Behaviours Questionnaire" OR (suicide W0 intent W0 scale*) OR "Suicide Intervention Response Inventory" OR "Suicide Ideation Scale" OR "Suicide Probability Scale" OR "Suicide Status Form" OR "Suicide Risk Assessment Scale" OR "Tool for the Assessment of Suicide" OR "Concise Health Risk Tracking Scale" OR "suicide assessment scale" OR "Karolinska Interpersonal Violence Scale") OR TI ((("symptom Driven Diagnostic System" W2 Suicide N0 Item*)) OR (("Scale for Impact of Suicidality") W1 ("Management, assessment and Planning of care")) OR ((Hamilton*) W2 ("rating scale" OR "depression scale" OR "depression rating")) OR ((Beck*) W2 ("Suicide Scale" OR "Hopelessness Scale" OR "Anxiety Inventory" OR "suicidal ideation scale" OR "Suicidal Intent Scale" OR "suicide intent scale" OR "suicide ideation" OR "Depression Inventory")) OR (Beck* W0 scale* W5 suicid*)) | 2,473 |
|  | 1 OR 2 | 185,888 |
| Outcome: Suicide, Self-harm | | |
|  | MM "Suicide" OR MM "Attempted Suicide" OR MM "Suicidal Ideation" OR MM "Self Injurious Behavior" OR TI (suicid* OR parasuicid* OR self-harm*) | 30,276 |
| Combined sets | | |
|  | 3 AND 4 | 1,104 |
|  | 5 AND Limiters - Publication Year: 1990-; Peer Reviewed; Language: Danish, English, French, German, Norwegian, Swedish | 729 |

The search result, usually found at the end of the documentation, forms the list of abstracts.

AB = Abstract

AU = Author

DE = Term from the thesaurus

MH = Term from the “Cinahl Headings” thesaurus

MM = Major Concept

TI = Title

TX = All Text. Performs a keyword search of all the  database's searchable fields

ZC = Methodology Index

* = Truncation

“ “ = Citation Marks; searches for an exact phrase

PubMed via NLM 16 December 2014 - Suicide Risk Asessment Tools

| Search terms | | Items found |
| --- | --- | --- |
| Intervention: screening, test, scales | | |
|  | Mass Screening[MeSH:NoExp] OR "Questionnaires"[MeSH] OR "Risk Assessment"[MeSH Terms] OR "Psychiatric Status Rating Scales"[Mesh] OR "Psychological Tests"[Mesh] OR "Psychometrics"[Mesh] OR Scale*[ti] OR Instrument[ti] OR Instruments[ti] OR Tool[ti] OR tools[ti] OR Measurement*[ti] OR Clinical predictor*[ti] OR Risk assessment[ti] OR risk screening[ti] OR Questionnaire*[ti] OR inventory[ti] OR psychometric*[ti] OR status form[ti] OR Checklist*[ti] OR score*[ti] | 1036182 |
|  | Adult Suicide Ideation Questionnaire[tiab] OR Columbia-Suicide Severity Rating Scale[tiab] OR Ducher's suicidal risk assessment scale[tiab] OR Firestones Assessment of Self-Destructive Thoughts[tiab] OR Geriatric Scale for suicide intent[tiab] OR High-Risk Construct Scale[tiab] OR interRAI Mental Health Severity of Self-Harm Scale[tiab] OR Manchester Self Harm Rule[tiab] OR Montgomery Asberg depression rating scale[tiab] OR Mental Health Environment of Care Checklist[tiab] OR Modified scale for Suicide Ideation[tiab] OR Nurses Global Assessment of Suicide Risk[tiab] OR Parasuicide History Inventory[tiab] OR Paykel Suicide Items[tiab] OR PHQ-9[tiab] OR Positive and Negative Suicide Ideation Inventory[tiab] OR PRISM-S[tiab] OR Quiz on Depression and Suicide in Late Life[tiab] OR Reasons for Living Inventory[tiab] OR risk-rescue[tiab] OR SAD PERSONS scale[tiab] OR Scale for Suicide Ideation[tiab] OR Schizophrenia Suicide Risk Scale[tiab] OR Self-Inflicted Injury Severity form[tiab] OR Self-Injury Implicit Association Test[tiab] OR Self-Monitoring Suicide Ideation Scale[tiab] OR Stroop test[tiab] OR Suicidal Behaviour Questionnaire[tiab] OR Suicide Behavior Interview[tiab] OR suicide Implicit Association Test[tiab] OR Suicide Intent Scale[tiab] OR Suicide opinion Questionnaire[tiab] OR Suicide Potential Lethality Scale[tiab] OR Suicide Probability Scale[tiab] OR Suicidal Ideation Questionnaire[tiab] OR Suicidal Ideation Screening Questionnaire[tiab] OR Suicidal Behaviours Questionnaire[ti] OR suicide intent scale*[ti] OR Suicide Intervention Response Inventory[tiab] OR Suicide Ideation Scale[tiab] OR Suicide Probability Scale[tiab] OR Suicide Status Form[tiab] OR Suicide Risk Assessment Scale[tiab] OR suicide assessment scale[tiab] OR Tool for the Assessment of Suicide Risk[tiab] | 3884 |
|  | ((symptom Driven Diagnostic System[ti]) AND (Suicide Items[ti])) OR ((Scale for Impact of Suicidality[ti]) AND (Management, assessment and Plannng of care[ti])) OR (Hamilton*[tiab] AND (rating scale[tiab] OR depression scale[tiab])) OR ((Beck[tiab] OR Beck'[tiab]) AND (Suicide Scale[ti] OR Hopelessness Scale[ti] OR Anxiety Inventory[tiab] OR suicidal ideation scale[tiab] OR Suicidal Intent Scale[tiab] OR Depression Inventory[tiab])) | 15069 |
|  | 1 OR 2 OR 3 | 1043436 |
| Outcome: Suicide, Self-harm | | |
|  | "Suicide"[Mesh:NoExp] OR "Suicidal Ideation"[Mesh] OR "Suicide, Attempted"[Mesh] OR "Self-Injurious Behavior"[Mesh:NoExp] OR suicid*[ti] OR parasuicid*[ti] OR self-harm*[ti] | 53779 |
| Combined sets | | |
|  | 4 AND 5 | 7822 |

The search result, usually found at the end of the documentation, forms the list of abstracts.

[MeSH] = Term from the Medline controlled vocabulary, including terms found below this term in the MeSH hierarchy

[MeSH:NoExp] = Does not include terms found below this term in the MeSH hierarchy

[MAJR] = MeSH Major Topic

[TIAB] = Title or abstract

[TI] = Title

[AU] = Author

[TW] = Text Word

Systematic[SB] = Filter for retrieving systematic reviews

* = Truncation
