# Supplementary material for: Instruments for the assessment of suicide risk: A systematic review evaluating the certainty of the evidence
Source: PLoS One. 2017 Jul 19;12(7):e0180292. doi: 10.1371/journal.pone.0180292 (PMC5517300; doi:10.1371/journal.pone.0180292)
Supplement: S2 Table — (DOCX) [file pone.0180292.s006.docx]

## S 2 Table Excluded studies

| **Reference** | **Reason(s) for exclusion** |
| --- | --- |
| Aish AM, Wasserman D, Renberg ES. Does Beck's Hopelessness Scale really measure several components? Psychol Med 2001;31:367-72. | Incorrect study design |
| Akizuki N, Yamawaki S, Akechi T, Nakano T, Uchitomi Y. Development of an Impact Thermometer for use in combination with the Distress Thermometer as a brief screening tool for adjustment disorders and/or major depression in cancer patients. J Pain Symptom Manage 2005;29:91-9. | Incorrect study design |
| Asarnow J, McArthur D, Hughes J, Barbery V, Berk M. Suicide attempt risk in youths: utility of the Harkavy-Asnis suicide scale for monitoring risk levels. Suicide Life Threat Behav 2012;42:684-98. | Incorrect outcome mesasures |
| Baca-Garcia E, Diaz-Sastre C, Garcia R, Blasco H, Braquehais C, Oquendo MA, et al. Suicide attempts and impulsivity. Eur Arch Psychiatry Clin Neurosci 2005;255:152-6. | Incorrect outcome mesasures |
| Ballard ED, Horowitz LM, Jobes DA, Wagner BM, Pao M, Teach SJ. Association of positive responses to suicide screening questions with hospital admission and repeated emergency department visits in children and adolescents. Pediatric Emergency Care 2013;29:1070-1074. | Incorrect study design |
| Beck AT, Brown GK, Steer RA. Psychometric characteristics of the Scale for Suicide Ideation with psychiatric outpatients. Behav Res Ther 1997;35:1039-46. | Incorrect study design |
| Beck AT, Steer RA, Trexler LD. Alcohol abuse and eventual suicide: a 5- to 10-year prospective study of alcohol-abusing suicide attempters. Journal of studies on alcohol. 1989 May;50(3):202-9. | irrelavant PICO |
| Beck AT, Weishaar ME. Suicide risk assessment and prediction. Crisis 1990;11:22-30. | Incorrect study design |
| Bevans KB, Diamond G, Levy S. Screening for adolescents' internalizing symptoms in primary care: item response theory analysis of the behavior health screen depression, anxiety, and suicidal risk scales. J Dev Behav Pediatr 2012;33:283-90. | Incorrect study design |
| Bradvik L, Berglund M. Suidical ideation in severe depression. Eur Arch Psychiatry Clin Neurosci 2000;250:139-43. | Incorrect study design |
| Bullard MJ. Problems of suicide risk management in the emergency department without fixed, full-time emergency physician. Changgeng Yi Xue Za Zhi 1993;16:30-8. | Incorrect study design |
| Connor J, Rueter M. Predicting adolescent suicidality: comparing multiple informants and assessment techniques. J Adolesc 2009;32:619-31. | Incorrect outcome measures |
| Cooper J, Kapur N, Mackway-Jones K. A comparison between clinicians' assessment and the Manchester Self-Harm Rule: a cohort study. Emerg Med J 2007;24:720-1. | Dual publikation |
| Diaz FJ, Baca-Garcia E, Diaz-Sastre C, Garcia R, Blasco H, Braquehais C, et al. Dimensions of suicidal behavior according to patient reports. Eur Arch Psychiatry Clin Neurosci 2003;253:197-202. | Incorrect study design |
| Eisenberg MG, Hubbard KM, Epstein D. Detection of suicidal risk among hospitalized veterans: preliminary experience with a suicide prediction scale. Journal of Rehabilitation 1990;56:63-68. | Irrelevant population |
| Engström G, Nyman GE, Träskman-Bendz L. The Marke-Nyman Temperament (MNT) Scale in suicide attempters. Acta Psychiatrica Scandinavica 1996;94:320-325. | Incorrect outcome measures |
| Eyman JR, Eyman SK. Personality assessment in suicide prediction. Suicide Life Threat Behav 1991;21:37-55. | Irrelevant population |
| Gaillard M, Martel S, Reynaud P, Mercadal L, Liger C, Herve C. [Severe suicides: short- and long-term outcome. Assessment of their quality of life]. Agressologie 1990;31:749-52. | Irrelevant population |
| Granier E, Boulenger JP. [Suicidal Risk Scale]. Encephale 2002;28:29-38. | Incorrect outcome |
| Gutierrez Peter M, Osman A. Getting the best return on your screening investment: An analysis of the Suicidal Ideation Questionnaire and Reynolds Adolescent Depression Scale. School Psychology Review 2009;38:200-217. | Incorrect study design |
| Haight BK. Suicide risk in frail elderly people relocated to nursing homes. Geriatr Nurs 1995;16:104-7. | Incorrect study design |
| Handley Tonelle E, Attia John R, Inder Kerry J, Kay-Lambkin Frances J, Barker D, Lewin Terry J, et al. Longitudinal course and predictors of suicidal ideation in a rural community sample. Australian & New Zealand Journal of Psychiatry 2013;47:1032-1040. | Incorrect outcome |
| Harbauer G, Ring M, Schuetz C, Andreae A, Haas S. Suicidality assessment with PRISM-S - simple, fast, and visual: a brief nonverbal method to assess suicidality in adolescent and adult patients. Crisis 2013;34:131-6. | Incorrect study design |
| Harriss L, Hawton K, Zahl D. Value of measuring suicidal intent in the assessment of people attending hospital following self-poisoning or self-injury. Br J Psychiatry 2005;186:60-6. | Dual publication |
| Heisel MJ, Duberstein PR, Conner KR, Franus N, Beckman A, Conwell Y. Personality and reports of suicide ideation among depressed adults 50 years of age or older. J Affect Disord 2006;90:175-80. | Incorrect study design |
| Heisel MJ, Flett GL, Besser A. Cognitive functioning and geriatric suicide ideation: testing a mediational model. Am J Geriatr Psychiatry 2002;10:428-36. | Incorrect study design |
| Heisel MJ, Flett GL, Duberstein PR, Lyness JM. Does the geriatric depression scale (GDS) distinguish between older adults with high versus low levels of suicidal ideation? Am J Geriatr Psychiatry 2005;13:876-83. | Incorrect study design |
| Heisel MJ, Flett GL. A psychometric analysis of the Geriatric Hopelessness Scale (GHS): towards improving assessment of the construct. J Affect Disord 2005;87:211-20. | Incorrect study design |
| Herman Steven M. Is the SADPERSONS Scale accurate for the veterans affairs population? Psychological Services 2006;3:137-141. | Incorrect study design |
| Hintikka J, Antikainen R, Lehtonen J. Can postdischarge suicide attempts of nonpsychotic inpatients be predicted? Nordic Journal of Psychiatry 1997;51:409-413. | Irrelevant population |
| Hjelmeland H, Stiles T, Bille-Brahe U, Ostamo A, Renberg E, Wasserman D. Parasuicide: The value of suicidal intent and various motives as predictors of future suicidal behaviour. Archives of Suicide Research. 1998 1998/09/01;4(3):209-25. | Incorrect study design |
| Holdsworth N, Collis B, Allott R. The development and evaluation of a brief risk screening instrument for the psychiatric inpatient setting. Journal of Psychiatric & Mental Health Nursing 1999;6:43-52. | Incorrect study design |
| Holi MM, Pelkonen M, Karlsson L, Kiviruusu O, Ruuttu T, Heila H, et al. Psychometric properties and clinical utility of the Scale of Suicidal Ideation (SSI) in adolescents. BMC Psychiatry 2005;5. | Incorrect study design |
| Horesh N, Levi Y, Apter A. Medically serious versus non-serious suicide attempts: relationships of lethality and intent to clinical and interpersonal characteristics. J Affect Disord 2012;136:286-93. | Incorrect study design |
| Horowitz LM, Ballard ED, Pao M. Suicide screening in schools, primary care and emergency departments. Curr Opin Pediatr 2009;21:620-7. | Incorrect study design |
| Innamorati M, Pompili M, Serafini G, Lester D, Erbuto D, Amore M, et al. Psychometric properties of the suicidal history self-rating screening scale. Arch Suicide Res 2011;15:87-92. | Incorrect study design |
| Jobes DA, Jacoby AM, Cimbolic P, Hustead LAT. Assessment and treatment of suicidal clients in a university counseling center. Journal of Counseling Psychology 1997;44:368-377. | Incorrect study design |
| Juhnke Gerald A, Hovestadt Alan J. Using the SAD PERSONS Scale to promote supervisee suicide assessment knowledge. The Clinical Supervisor 1995;13:31-40. | Irrelevant population |
| Kaplan Z, Benbenishty R, Waysman M, Solomon Z, Bleich A. Clinicians' assessments of suicide risk: can self-report measures replace the experts? Isr J Psychiatry Relat Sci 1992;29:159-66. | Incorrect study design |
| Kapur N, Cooper J, Rodway C, Kelly J, Guthrie E, Mackway-Jones K. Predicting the risk of repetition after self harm: cohort study. Bmj 2005;330:394-5. | Incorrect study design |
| King CA, Hill RM, Wynne HA, Cunningham RM. Adolescent suicide risk screening: the effect of communication about type of follow-up on adolescents' screening responses. J Clin Child Adolesc Psychol 2012;41:508-15. | Irrelevant outcome |
| King CA, Jiang Q, Czyz EK, Kerr DC. Suicidal Ideation of Psychiatrically Hospitalized Adolescents has One-Year Predictive Validity for Suicide Attempts in Girls Only. J Abnorm Child Psychol 2013. | Irrelevant outcome |
| King CA, Jiang Q, Czyz EK, Kerr DC. Suicidal Ideation of Psychiatrically Hospitalized Adolescents has One-Year Predictive Validity for Suicide Attempts in Girls Only. J Abnorm Child Psychol 2013. | Irrelevant outcome |
| Koslowsky M, Bleich A, Greenspoon A, Wagner B, Apter A, Solomon Z. Assessing the validity of the Plutchik Suicide Risk Scale. J Psychiatr Res 1991;25:155-8. | Incorrect study design |
| Kumar CT, Mohan R, Ranjith G, Chandrasekaran R. Characteristics of high intent suicide attempters admitted to a general hospital. J Affect Disord 2006;91:77-81. | Incorrect study design |
| Lai YM, Ko SM. What you need to know--assessment of suicide risk. Singapore Med J 1999;40:379-80. | No test, general info |
| Large MM, Dall B, Nielssen OB. Risk assessment for violence and self-harm in first episode psychosis and the need for early psychosis intervention services. Aust N Z J Psychiatry 2013. | Letter |
| Larzelere RE, Andersen JJ, Ringle JL, Jorgensen DD. The child suicide risk assessment: a screening measure of suicide risk in pre-adolescents. Death Stud 2004;28:809-27. | Not prospective |
| Lejoyeux M, Le H, M F. [Suicide risk in children and adolescents. Identification and management]. Rev Prat 2002;52:791-6. | General information |
| Lester D. Derivation of a proxy measure of suicidal ideation from the Suicide Opinion Questionnaire. Percept Mot Skills 2004;99:1046. | Irrelevant population |
| Lester D. Self-monitoring, depression and suicidal ideation. Psychol Rep 1990;67:410. | Not prospective |
| Lindenmayer JP, Czobor P, Alphs L, Nathan AM, Anand R, Islam Z, et al. The InterSePT scale for suicidal thinking reliability and validity. Schizophr Res 2003;63:161-70. | Irrelevant outcome |
| Madsen T, Nordentoft M. Suicidal changes in patients with first episode psychosis: clinical predictors of increasing suicidal tendency in the early treatment phase. Early Interv Psychiatry 2012;6:292-9. | Incorrect study design |
| Mahgoub N, Avari J, Kalayam B, Klimstra S. Suicide risk-assessment in patients with primary progressive aphasia. J Neuropsychiatry Clin Neurosci 2012;24:E26-7. | Letter |
| Marfe E. Assessing risk following deliberate self harm. Paediatr Nurs 2003;15:32-4. | Guidlines |
| Marusic A, Marusic D. Suicidology--expanding its research tools. Crisis 2002;23:95-7. | Editorial |
| Mathias CW, Michael F, Sheftall AH, Hill-Kapturczak N, Crum P, Dougherty DM. What's the harm in asking about suicidal ideation? Suicide Life Threat Behav 2012;42:341-51. | Irrelevant outcome |
| Matson JL, Bamburg JW, Cherry KE, Paclawskyj TR. A validity study on the Questions About Behavioral Function (QABF) Scale: predicting treatment success for self-injury, aggression, and stereotypies. Res Dev Disabil 1999;20:163-75. | Irrelevant outcome |
| McMillan D, Gilbody S, Beresford E, Neilly L. Can we predict suicide and non-fatal self-harm with the Beck Hopelessness Scale? A meta-analysis. Psychol Med 2007;37:769-78. | Meta-analysis |
| McPherson A. An overview of the assessment tools available to mental health professionals to help determine patients at risk of suicide. Int J Psychiatr Nurs Res 2005;10:1129-42. | Review |
| Mills Jeremy F, Kroner Daryl G. Screening for suicide risk factors in prison inmates: Evaluating the efficiency of the Depression, Hopelessness and Suicide Screening Form (DHS). Legal and Criminological Psychology 2005;10:1-12. | No prospective |
| Morriss R, Kapur N, Byng R. Assessing risk of suicide or self harm in adults. Bmj 2013;347:f4572. | Review |
| Myers K, McCauley E, Calderon R, Treder R. The 3-year longitudinal course of suicidality and predictive factors for subsequent suicidality in youths with major depressive disorder. J Am Acad Child Adolesc Psychiatry 1991;30:804-10. | Irrelevant outcome |
| Naud H, Daigle MS. How to improve testing when trying to predict inmate suicidal behavior. Int J Law Psychiatry 2013. | Irrelevant population |
| Neimeyer RA, Bonnelle K. The Suicide Intervention Response Inventory: a revision and validation. Death Stud 1997;21:59-81. | Irrelevant outcome |
| O'Connor E, Gaynes BN, Burda BU, Soh C, Whitlock EP. Screening for and treatment of suicide risk relevant to primary care: a systematic review for the U.S. Preventive Services Task Force. Ann Intern Med 2013;158:741-54. | Review |
| O'Connor SS, Jobes DA, Comtois KA, Atkins DC, Janis K, Chessen CE, et al. Identifying outpatients with entrenched suicidal ideation following hospitalization. Suicide Life Threat Behav 2012;42:173-84. | Too few participants n=40 |
| O'Donnell H. Suicide risk assessment: a challenge for all health care professionals. Nursing Review 1998;16:40-45. | Review |
| Ojehagen A, Danielsson M, Traskman-Bendz L. Deliberate self-poisoning: treatment follow-up of repeaters and nonrepeaters. Acta Psychiatr Scand 1992;85:370-5. | Missing data |
| O'Mara RM, Hill RM, Cunningham RM, King CA. Adolescent and parent attitudes toward screening for suicide risk and mental health problems in the pediatric emergency department. Pediatr Emerg Care 2012;28:626-32. | Irrelevant study, |
| Osgood NJ, Brant BA. Suicidal behavior in long-term care facilities. Issues Law Med 1990;6:153-62. | Irrelevant poulation |
| Osman A, Bagge CL, Gutierrez PM, Konick LC, Kopper BA, Barrios FX. The Suicidal Behaviors Questionnaire-Revised (SBQ-R): validation with clinical and nonclinical samples. Assessment 2001;8:443-54. | No prospective outcome |
| Osman A, Barrios FX, Gutierrez PM, Wrangham JJ, Kopper BA, Truelove RS, et al. The Positive and Negative Suicide Ideation (PANSI) inventory: psychometric evaluation with adolescent psychiatric inpatient samples. J Pers Assess 2002;79:512-30. | No prospective outcome |
| Osman A, Gutierrez PM, Barrios FX, Bagge CL, Kopper BA, Linden S. The Inventory of Suicide Orientation-30: further validation with adolescent psychiatric inpatients. J Clin Psychol 2005;61:481-97. | No prospective outcome |
| Osman A, Gutierrez PM, Muehlenkamp JJ, Dix-Richardson F, Barrios FX, Kopper BA. Suicide Resilience Inventory-25: development and preliminary psychometric properties. Psychol Rep 2004;94:1349-60. | No prospective outcome |
| Perry AE, Marandos R, Coulton S, Johnson M. Screening tools assessing risk of suicide and self-harm in adult offenders: a systematic review. Int J Offender Ther Comp Criminol 2010;54:803-28. | Review |
| Petrie K, Brook R. Sense of coherence, self-esteem, depression and hopelessness as correlates of reattempting suicide. Br J Clin Psychol 1992;31 ( Pt 3):293-300. | Missing data |
| Pfeiffer PN, Brandfon S, Garcia E, Duffy S, Ganoczy D, Myra K, et al. Predictors of suicidal ideation among depressed veterans and the interpersonal theory of suicide. J Affect Disord 2013. | Outcome is ideation |
| Phillips NL, Stargatt R, Brown A. Risk assessment of self- and other-directed aggression in adolescent psychiatric inpatient units. Aust N Z J Psychiatry 2012;46:40-6. | Retrospetive |
| Piersma HL, Boes JL. Utility of the Inventory of Suicide Orientation-30 (ISO-30) for adolescent psychiatric inpatients: linking clinical decision making with outcome evaluation. J Clin Psychol 1997;53:65-72. | Irrelevant outcome |
| Pinninti N, Steer RA, Rissmiller DJ, Nelson S, Beck AT. Use of the Beck Scale for suicide ideation with psychiatric inpatients diagnosed with schizophrenia, schizoaffective, or bipolar disorders. Behav Res Ther 2002;40:1071-9. | Irrelevant outcome |
| Posner K, Oquendo MA, Gould M, Stanley B, Davies M. Columbia Classification Algorithm of Suicide Assessment (C-CASA): classification of suicidal events in the FDA's pediatric suicidal risk analysis of antidepressants. Am J Psychiatry 2007;164:1035-43. | No prospective outcome |
| Potter LB, Kresnow M-j, Powell KE, O'Carroll PW, Lee RK, Frankowski RF, et al. Identification of nearly fatal suicide attempts: Self-Inflicted Injury Severity Form. Suicide and Life-Threatening Behavior 1998;28:174-186. | No prospective outcome |
| Preston E, Hansen L. A systematic review of suicide rating scales in schizophrenia. Crisis 2005;26:170-80. | Review |
| Range Lillian M. The Family of Instruments That Assess Suicide Risk. Journal of Psychopathology and Behavioral Assessment 2005;27:133-140. | Review |
| Range LM, Knott EC. Twenty suicide assessment instruments: evaluation and recommendations. Death Stud 1997;21:25-58. | Review |
| Razykov I, Ziegelstein RC, Whooley MA, Thombs BD. The PHQ-9 versus the PHQ-8--is item 9 useful for assessing suicide risk in coronary artery disease patients? Data from the Heart and Soul Study. J Psychosom Res 2012;73:163-8. | No prospective outcome |
| Righini NC, Narring F, Navarro C, Perret-Catipovic M, Ladame F, Jeannin A, et al. Antecedents, psychiatric characteristics and follow-up of adolescents hospitalized for suicide attempt of overwhelming suicidal ideation. Swiss Med Wkly 2005;135:440-7. | No suicidal scales |
| Romanowicz M, O'Connor SS, Schak KM, Swintak CC, Lineberry TW. Use of the Suicide Status Form-II to investigate correlates of suicide risk factors in psychiatrically hospitalized children and adolescents. J Affect Disord 2013;151:467-73. | No follow up |
| Rowlands RP. Assessing risk of suicide. Deal with self harm in prisons. Bmj 1995;310:127. | Letter |
| Ruengorn C, Sanichwankul K, Niwatananun W, Mahatnirunkul S, Pumpaisalchai W, Patumanond J. A risk-scoring scheme for suicide attempts among patients with bipolar disorder in a Thai patient cohort. Psychology Research and Behavior Management 2012;5. | Not prospective |
| Ryan CJ, Large MM, Callaghan S. Suicide risk assessment: where are we now? Med J Aust 2013;199:534. | Letter |
| Sakinofsky I, Heila H, Krishnan R. Estimating Suicidality as an Outcome Measure in Clinical Trials of Suicide in Schizophrenia. Schizophrenia Bulletin 2004;30:587-598. | No prospective outcome |
| Samuelsson M, Wiklander M, Asberg M, Saveman BI. Psychiatric care as seen by the attempted suicide patient. J Adv Nurs 2000;32:635-43. | Rating scale is not the topic |
| Schennach-Wolff R, Jager M, Seemuller F, Obermeier M, Schmauss M, Laux G, et al. Outcome of suicidal patients with schizophrenia: results from a naturalistic study. Acta Psychiatr Scand 2010;121:359-70. | Irrelevant outcomes |
| Schlenger WE, Williams RL, Blitstein JL. Iatrogenic risk of screening for youth suicide. Jama 2005;294:2578-9; author reply 2579. | Letter |
| Schulberg HC, Lee PW, Bruce ML, Raue PJ, Lefever JJ, Williams JW, et al. Suicidal ideation and risk levels among primary care patients with uncomplicated depression. Ann Fam Med 2005;3:523-8. | Irrelevant population |
| Scocco P, Marietta P, Tonietto M, Dello B, De L. The role of psychopathology and suicidal intention in predicting suicide risk: a longitudinal study. Psychopathology 2000;33:143-50. | Irrelevant outcome |
| Shaffer D. 'Predictive validity of the Suicide Probability Scale among adolescents in group home treatment': Discussion. Journal of the American Academy of Child & Adolescent Psychiatry 1996;35:172-174. | Discussion, no result |
| Shah A, Hoxey K, Mayadunne V. Some predictors of mortality in acutely medically ill elderly inpatients. Int J Geriatr Psychiatry 2000;15:493-9. | Irrelevant outcome |
| Shultz E, Pandya M, Mehta N. Technology and teaching: suicide risk assessment. Med Educ 2013;47:1132-3. | Irrelevant teaching intervention |
| Simon RI. Suicide risk assessment: what is the standard of care? J Am Acad Psychiatry Law 2002;30:340-4. | Editorial |
| Simpson S, Stacy M. Avoiding the malpractice snare: documenting suicide risk assessment. J Psychiatr Pract 2004;10:185-9. | Irrelevant PICO |
| Skarbo T, Rosenvinge JH, Holte A. Suicide attempts and life events 5-9 years after referral for emergency psychiatric outpatient treatment. Nord J Psychiatry 2005;59:264-71. | Retrospective study |
| Smith Eric G, Kim Hyungjin M, Ganoczy D, Stano C, Pfeiffer Paul N, Valenstein M. Suicide risk assessment received prior to suicide death by veterans health administration patients with a history of depression. Journal of Clinical Psychiatry 2013;74:226-232. | Retrospective study |
| Sonneck G, Horn W. Contribution to suicide risk assessment: I. A simple method to predict crises after suicide attempts (parasuicides). Crisis 1990;11:31-3. | Statiscal model |
| Sorvaniemi M. [A suicidal risk]. Duodecim 2000;116:1011-3. | Not included langugate |
| Spijker J, de G, Ten H, Nolen WA, Speckens A. Predictors of suicidality in depressive spectrum disorders in the general population: results of the Netherlands Mental Health Survey and Incidence Study. Soc Psychiatry Psychiatr Epidemiol 2010;45:513-21. | No scale tested |
| Stoppe G, Sandholzer H, Huppertz C, Duwe H, Staedt J. Family physicians and the risk of suicide in the depressed elderly. J Affect Disord 1999;54:193-8. | Irrelevant design |
| Suominen K, Isometsa E, Ostamo A, Lonnqvist J. Level of suicidal intent predicts overall mortality and suicide after attempted suicide: a 12-year follow-up study. BMC Psychiatry 2004;4:11. | Irrelevant outcome |
| Szmukler G. Risk assessment for suicide and violence is of extremely limited value in general psychiatric practice. Aust N Z J Psychiatry 2012;46:173-4. | Letter |
| Taiminen T, Huttunen J, Heila H, Henriksson M, Isometsa E, Kahkonen J, et al. The Schizophrenia Suicide Risk Scale (SSRS): development and initial validation. Schizophr Res 2001;47:199-213. | Retrospective outcome |
| Tejedor MC, Diaz A, Castillon JJ, Pericay JM. Attempted suicide: repetition and survival--findings of a follow-up study. Acta psychiatrica Scandinavica. 1999 Sep;100(3):205-11. | Incorrect study design |
| Thienhaus OJ, Piasecki M. Assessment of suicide risk. Psychiatr Serv 1997;48:293-4. | General clinical management |
| Tredget J. Suicide risk assessment and the use of patient contracts. Nurs Times 1999;95:50-1. | No original research, no testing of instruments in clinical population. |
| Tuisku V, Pelkonen M, Kiviruusu O, Karlsson L, Marttunen M. Alcohol use and psychiatric comorbid disorders predict deliberate self-harm behaviour and other suicidality among depressed adolescent outpatients in 1-year follow-up. Nord J Psychiatry 2012;66:268-75. | Irrelevant outcome |
| Tuisku V, Pelkonen M, Kiviruusu O, Karlsson L, Marttunen M. Alcohol use and psychiatric comorbid disorders predict deliberate self-harm behaviour and other suicidality among depressed adolescent outpatients in 1-year follow-up. Nord J Psychiatry 2012;66:268-75. | Irrelevant outcome |
| Turner Ralph M, Korslund Kathryn E, Barnett Belinda E, Josiassen Richard C. Assessment of suicide in schizophrenia: Development of the Interview for Suicide in Schizophrenia. Cognitive and Behavioral Practice 1998;5:139-169. | No prospective outcome |
| Valente SM. Assessing patients for suicide risk. Nursing 2010;40:36-40; quiz 40. | General clinical management |
| Van G, Schotte C, Maes M. The prediction of suicidal intent in depressed patients. Acta Psychiatr Scand 1997;96:254-9. | Irrelevant study design, no follow up |
| Weeks SK, Andreson MA, Harmon LS, Michaels TK. Getting inside depression and suicide ideation. One comprehensive screening approach targets patients 4 years and older. Holistic nursing practice 2005;19:5-9. | Irrelevant PICO |
| Werry JS. Youth suicide report 1997. N Z Med J 1999;112:167. | Letter |
| White GL, Jr, Murdock RT, Richardson GE, Ellis GD, Schmidt LJ. Development of a tool to assess suicide risk factors in urban adolescents. Adolescence 1990;25:655-66. | No follow up |
| Wiktorsson S, Marlow T, Runeson B, Skoog I, Waern M. Prospective cohort study of suicide attempters aged 70 and above: one-year outcomes. J Affect Disord 2011;134:333-40. | Missing data |
| Vining RM. Assessing risk of suicide. Samaritans' scoring system helps develop judgment. Bmj 1995;310:126-7. | Letter |
| Winters NC, Myers K, Proud L. Ten-year review of rating scales. III: scales assessing suicidality, cognitive style, and self-esteem. J Am Acad Child Adolesc Psychiatry 2002;41:1150-81. | Review |
| Witte TK, Fitzpatrick KK, Joiner TE, Jr, Schmidt NB. Variability in suicidal ideation: a better predictor of suicide attempts than intensity or duration of ideation? J Affect Disord 2005;88:131-6. | Incorrect population |
| Yaseen ZS, Gilmer E, Modi J, Cohen LJ, Galynker, II. Emergency room validation of the revised Suicide Trigger Scale (STS-3): a measure of a hypothesized suicide trigger state. PLoS One 2012;7:e45157. | Prospective outcome for only 18 out of 183 |
| Yen S, Shea MT, Walsh Z, Edelen MO, Hopwood CJ, Markowitz JC, et al. Self-harm subscale of the Schedule for Nonadaptive and Adaptive Personality (SNAP): predicting suicide attempts over 8 years of follow-up. J Clin Psychiatry 2011;72:1522-8. | Irrelevant population |
