# Supplementary material for: Instruments for the assessment of suicide risk: A systematic review evaluating the certainty of the evidence
Source: PLoS One. 2017 Jul 19;12(7):e0180292. doi: 10.1371/journal.pone.0180292 (PMC5517300; doi:10.1371/journal.pone.0180292)
Supplement: S3 Table — (DOCX) [file pone.0180292.s007.docx]

# S3 Table Characteristics of the included studies

| **Tool** | **First author**  **Year**  **Country** | **Design**  **Setting**  **Population**  **Evaluation time**  **Study period** | **Index test**  **Reference test**  **Drop-out rate** | **Results**  **(Sensitivity, specificity, PPV, NPV)** | **Comments** |
| --- | --- | --- | --- | --- | --- |
| ***BDI*** | Beck, 1990  USA [1] | Design: Cohort  Setting: Center for cognitive therapy  Population: N=1958, outpatients who were evaluated at intake. Excluded severe risk of suicide  Participation rate: ND  Evaluation time: Mean 43±20.71 months (until December 1985)  Study period: September 1978 to February 1985 | Index test  N=1958, 58% female  Mean age:36.12±12.39  (range 15–84)  Reference test  Suicide, N=17, 47.1% female  Mean age: 40.35±11.61  (range 18–59)  Drop-out rate: 14/1958 (0.7%), (natural death: 12) | Cut off:≥23  Sensitivity: 0.76 [0.50, 0.93] Specificity: 0.62 [0.60, 0.65]  PPV:1.75%  NPV: 99% | Sampling method  Consecutive patients  Handling of missing data  Two drop-outs were excluded.  Other comments  10 of the 17 patients who died by suicide received cognitive therapy; only one was in treatment at the time of death. |
| ***BHS*** | Beck, 1990  USA [1] | Design: Cohort  Setting: Center for cognitive therapy  Population: N=1958, outpatients who were evaluated at intake.  Excluded severe risk of suicide  Participation rate: ND  Evaluation time: Mean 43±20.71 months (until December 31, 1985)  Study period: September 1978 to February 1985 | Index test  N=1958, 58% female  Mean age: 32.16±12.39 (range 15–84)  Reference test  Suicide, n=17, 47.1% female  Mean age: 40.35±11.61  (range 18–59)  Drop-out rate: 2/1958 (0.1%)  Natural death: 12 | Cut off: ≥9  Sensitivity: 0.94 [0.71, 1.00] Specificity:0.41 [0.39, 0.43]  PPV: 1.38%  NPV: 99.9% | Sampling method  Consecutive patients  Handling of missing data  Two dropouts were excluded from analyses.  Other comments  10 of the 17 patients who died by suicide received cognitive therapy; only one was in treatment at the time of death. |
| ***BHS*** | Beck, 1999  USA [2] | Design: Cohort  Setting: Center of cognitive therapy  Population: N=3701, outpatients who were evaluated at intake, 65% history of suicide attempts  Participation rate: ND  Evaluation time: 1979–1994  Study Period: 1975–1994 | Index test  N=3701, female:56.7%  Mean age: ND  Reference test  Suicide, n=30, female: 40%  Mean age: 41.1±13.68  Drop-out rate: 128/3701 (3%) | Cut off: derived from ROC analysis  Cut off for higher risk≥8  Sensitivity: 0.90 [0.73, 0.98] Specificity: 0.42 [0.40, 0.44]  PPV: 1.3%  NPV: 100% | Few inpatients in the sample |
| ***BHS*** | Keller, 1993  Germany [3] | Design: Cohort  Setting: Depression ward, Psychiatric Hospital  Population: N=76, depressive illness, admitted to the ward, none with a secondary psychiatric diagnosis.  Participation rate: ND  Evaluation time: 1 year  Study period: February to October 1986 | Index test  N=61, 65.6% female  Mean age: 47  Reference test  Suicide, n=2  % female and mean age: ND  Suicide attempts, n=8  % female: ND, mean age: ND  Drop-out rate: 15/76 | Cut off:≥9  *Suicide*  Sensitivity: 1.00 [0.16, 1.00]  Specificity: 0.27 [0.16, 0.40] PPV: 4.4%  NPV: 100%  *Suicide attempts*  Sensitivity: 0.80 [0.44, 0.97]  Specificity:0.27 [0.16, 0.42]  PPV: 18%  NPV: 88% | Handling of missing data  Unclear |
| ***BHS*** | Klonsky  2012  USA  [4] | Design: Cohort  Setting: 12 Inpatient facilities  Population: N=675  Patients with 1^st^ admission of psychosis, 29% with a history of attempted suicide  Participation rate: 72%  Evaluation time: Up to 10 years  Study Period: 1989-1995 | Index test  n=414, female: 43%  Mean age: 29.1±9.6 years  Reference test  Suicide, 10 years: n=6  Female:33%, Mean age: ND  Drop-out rate  261/675 (39%) at ten years | Cut off: ≥9  Sensitivity: 0.33 [0.04, 0.78] Specificity:0.88 [0.84, 0.91]  PPV: 3.92%  NPV: 98.9% | Other comments  For the 6 persons who completed suicide, hopelessness at baseline ranged from 0–11, with a median of 4. |
| ***BHS*** | Niméus  1997  Sweden [5] | Design: Cohort  Setting: Suicide research unit at university hospital  Population: N=212, Suicide attempters age>18, 84% history of suicide attempts  Participation rate: ND  Evaluation time: Range 1 month to 8 years  Study Period: ND | Index test  n=212, female: 56.6%  Mean age: 37.7±13.6 (range 18-80)  Reference test  Suicide, n=13, female: 69%  Mean age: ND  Drop-out rate: 0 | Cut off:9  Sensitivity: 0.77 [0.46, 0.95] Specificity: 0.42 [0.35, 0.49]  PPV: 8%  NPV: 96.5%  Cut off:13  Sensitivity: 0.77  Specificity: 0.61  PPV: 13% | Other comments  Includes only patients referred to the suicide research unit  Suicides identified by local dept of forensic medicine; persons who moved from the area could not be followed up. |
| ***BHS*** | Sidley, 1999  UK [6] | Design: Cohort  Setting: Accident department and ED  Population: Parasuicide patients  N=66  Participation rate: ND  Evaluation time: 12 months  Study Period: ND | Index test  N=66, female: 45.5%  Mean age: 33.6 (range 19-58)  Reference test  parasuicide  1 month. n=10  6 months: n=20  12 months: n=25  % female and mean age: ND  Drop-out rate  2/66 (3%) at 1 month  3/66 at 12 months | Cut off: 19 or 20, 1 months  Sensitivity: 0.60 [0.26, 0.88] Specificity:0.91 [0.80, 0.97]  PPV: 54.35%  NPV :92.72%  Cut off ≥2, 12 months  Sensitivity: 0.58 [0.37, 0.78] Specificity:0.87 [0.73, 0.96]  PPV: 73.12%  NPV: 77.26% |  |
| ***C-SSRS*** | Posner  2011  USA [7] | Design: Cohort  Setting: ND  Population: N=124, Adolescent suicide attempters  % female and mean age: ND  Evaluation time: 6-24 weeks  Study Period: ND | Index test  N=124  Reference test  Suicide  Suicide attempts  n=15 patients, 24 episodes  Drop-out rate: ND | Sensitivity: 100%  Specificity: 96% |  |
| ***ERRS*** | Carter  2002  Australia  [8] | Design: Cohort  Setting: Centralized referral center  Population: N=1331 adults  Self-poisoning patients  Evaluation time: 12 months  Study Period  3 year period  1996-1998 | Index test  n=1317, female: 62.3%  Median age (F/M): 32/33  Reference test  Repeated self-poisoning  n=180, female: 63%  Mean age: ND  Drop-out rate: 14/1331 (1.1%) | Cut off: clinical scoring  High risk, men≥8  High risk, women≥6  Sensitivity: 0.26 [0.20, 0.33]  Specificity:0.84 [0.82, 0.86]  PPV: 21%  NPV: 88%  Cut-off: research scoring  Sensitivity: 0.27 [0.21, 0.34] Specificity:0.86 [0.84, 0.88]  PPV: 23%  NPV:88% | Sampling method  consecutive |
| ***IAT*** | Randall  2013  Canada  [9] | Design: Cohort  Setting: 2 ED  Population: >17 years, N=180  Participation rate: 67%  Evaluation time: 3 months  Study Period: August 2009-May 2010 | Index test  n=127, female: 47.8%  Mean age: ND  Reference test  Self-harm, n=29, female: ND  Mean age: ND  Drop-out rate: 20/127 (15.8%) | Cut off: 0  Sensitivity: 43.3% (0.255,0.626)  Specificity: 78.8% (68.2, 87.1)  PPV: 37.7%  NPV: 82.5% |  |
| ***MINI*** | Roaldset  2012  Norway [10] | Design: Cohort  Setting: Acute psychiatric unit  Population: N=489, all acutely admitted patients, %female: ND, mean age: ND  Participation rate: 411/489  Evaluation time: 1 year  Study period: March 2006 to July 2008 | Index test  n=411  Reference test  Suicide behaviour or NSSI  3 months n=71  12 months: n=112  Suicide attempts or NSSI n=64  Drop-out rate: 1 year: 104/411 (25%) | Self- harm Cut off: ≥10  Sensitivity: 61%  Specificity: 75%  PPV: 38%  NPV: 86% | Other comments  Unclear whether the 2 suicides were included among the attempts in the index test, For multiple admissions, “index” subgroup was defined in retrospect as the most serious episode. |
| ***MSHR*** | Bilén, 2013  Sweden  [11] | Design: Cohort  Setting: 2 large ED  Population: Adults, DSH  N=328  Participation rate: ND  Evaluation time: 6 months  Study Period: Mars- – June 2011 | Index test  n=325, female: 70%  Mean age: ND  Reference test  Repeated DSH/suicide attempt  n=80  female: ND  Mean age: ND  Drop-out rate: 3/328 (1%) | Cut off: 0.14  Sensitivity: 0.94 [0.85, 0.98] Specificity: 0.18 [0.14, 0.24]  PPV: 27%  NPV: 90% | Sampling method  Consecutive sample |
| ***MSHR*** | Bilén, 2013  Sweden  [12] | Design: Cohort  Setting: ED  Population: N= 1524, Adults, DSH  35% history of DSH  57% treated with antidepressants  Evaluation time: 6 months  Study Period: Jan 2003 to Dec 2005 | Index test  n=1524, female: 65%  Mean age: ND  Reference test  Suicide +Repeated DSH  n=309, female: 70%  Mean age: 39.5 (18–91 years)  Suicide  n=12  Repeated DSH  n=297  Drop-out rate: 0% | Cut off: 0.14  Repeated DSH  Sensitivity: 0.89 [0.85, 0.92] Specificity:0.21 [0.19, 0.24]  PPV: 22% (275/1230)  NPV: 88% (260/294) | Sampling method  Consecutive sample |
| ***MSHR*** | Cooper  2006  UK  [13] | Design: Cohort  Setting: 5 ED  Population: N=11819 episodes of self-harm  Evaluation time: 6 months  Study Period: September 1997 to February 2001. | Index test  n=6933 patients, 9086 episodes  Derivation set: n=6933 episodes  Validation set: n=2153 episodes  Mean age: ND  Reference test  Suicide attempt/self-harm  n=1538 episodes  Median age: 32  Suicide  6 months n=22  3 years n=59  Drop-out rate  Derivation data set: 203 (3%)  Validation data set: 58 (3%) | Episodes  Derivation data set  Sensitivity: 0.94 [0.92, 0.95] Specificity: 0.25 [0.24, 0.26]  PPV: 20 (19, 21)  NPV: 95 (94, 96)  Validation data set  Sensitivity: 0.97 [0.94, 0.98] Specificity: 0.26 [0.24, 0.29]  PPV: 22 (19, 21)  NPV: 97 (96–99) | Other comments  The study used internal validation |
| ***MSHR*** | Randall  2012  Canada  [14] | Design: Cohort  Setting: 2 ED  Population: N=181, Adults presenting with self-harm or suicidal ideation  Evaluation time: 3 months  Study Period: August 2009 to May 2010 | Index test  n= 157, female: 48.9%  Mean age: 37.2 years  Reference test  Self-harm  n:34, % female: ND  Mean age: ND  Drop-out rate: 29/157 (18.5%) | Cut off: 1  Sensitivity: 95.1%  Specificity: 0.15 [0.09, 0.22]  PPV: 23.65%  NPV: 91.6% |  |
| ***MSHR*** | Steeg  2012  UK [15] | Design: Cohort  Setting: five EDs  Population: Adults, self-harm  N=18680 patients, 29571 presentations  Evaluation time: 6 months  Study Period: January 2003 and June 2007 | Index test  n= 24779 presentations  female: ND  Mean age: ND  Reference test  Suicide  n=76, 39% females  Mean age: ND  Suicide attempts/self-harm  n=7606 episode  female: ND  Mean age: ND  Drop-out rate: Unclear | Suicide attempts:  Derivation centre  Sensitivity: 0.98 (0.98, 0.99)  Specificity: 0.17 (0.16, 0.17)  PPV: 33.5%  NPV: 95.2%  Test centre  Sensitivity: 0.97 (0.96, 0.98)  Specificity: 0.20 (0.18, 0.21)  PPV: 37.5%  NPV: 93% |  |
| ***PHQ-9 item 9*** | Simon  2013  USA [16] | Design: Cohort  Setting: Primary care and mental health clinics within an integrated health care system  Population: Depression, age 13+  N=84 418 patients  188 611 (90%) of patients were receiving current/recent mental health treatment  Participation rate: Unclear. Data collected at 5% of depression visits in 2007 and 48% in 2011.  Evaluation time: Range 1 - 1703 days, median 360, mean 477  Study period: January 2007 to December 2011 | Index test  n= 84 418  % Female and mean age: ND  Reference tests  Suicide  n=46  % Female and mean age: ND  Suicide attempts  n=709  % Female and mean age: ND  Drop-out rate: 24% | Cut off: several days during the last 2 weeks  Suicide  Sensitivity: 0.80 [0.66, 0.91] Specificity: 0.70 [0.70, 0.71]  PPV: 0.15%  NPV: 97.8%  Suicide attempts  Sensitivity: 0.78 [0.74, 0.81]  Specificity: 0.71 [0.70, 0.71]  PPV: 2%  NPV: 99.76% | Other comments  Multiple ratings  (207 265 records).  Analyses are based on questionnaire completed closest to event date.  Events determined by electronic records, insurance claims and death certificates.  29% of suicide attempts missed by E codes. |
| ***ReACT Self-Harm Rule*** | Steeg , 2012  UK [15] | Design: Cohort  Setting: Five hospital EDs  Population: Adults, Self-harm  N=18 680 patients, 29 571 presentations, Female:59%  Median age: 32 years (range 16–97)  Evaluation time: 6 months  Study Period: January 2003 and June 2007 | Index test  Derivation set:  n=22 532 episodes  External test data:  n=7039 episodes  Reference test  Suicide  Derivation set: n= 66  External test data:  n=26  Total: N=92  female: 39%  Suicide attempts/self-harm  Derivation set:  n=6014 episodes  External test data:  n=2096 episodes  In total: female: 63%  Drop-out rate: ND | episodes  Any repetition/suicide attempts  Derivation data  Sensitivity: 0.95 (0.94, 0.95)  Specificity: 0.21 (0.21, 0.22)  PPV: 30%  NPV: 91%  External test data  Sensitivity: 0.90 (0.89, 0.91)  Specificity: 0.34 (0.32, 0.35)  PPV: 37%  NPV: 89%  Suicide  *Derivation data*  Sensitivity: 0.91 (0.81, 0.97)  Specificity: 0.15 (0.15, 0.16)  PPV: 0.4%  NPV: 99.8%  *External test data*  Sensitivity: 0.88 (0.7, 0.98)  Specificity: 0.24 (0.23, 0.25)  PPV: 0.5%  NPV: 99.6% |  |
| ***SAD PERSONS Scale*** | Bolton et al  2012  Canada  [17] | Design: Prospective cohort  Setting: Psychiatric services at ED two tertiary care hospitals  Population: Adults, N=4019 presentations  Evaluation time: 6 months  Study period: Jan 1999 to December 2010 | Index test  n=2846,  % female: ND  Mean age: ND      Reference test  Suicide attempts  n= 87 individuals, 80 completed SAD PERSONS Scale  female: 52.9 %  Mean age: ND    Drop-out rate: Unclear | Cut off: High vs moderate+low  Sensitivity: 0.13 [0.06, 0.22]  Specificity: 0.94 [0.93, 0.94]  PPV: 5.3%  NPV: 97.4% | Sampling method  Consecutive sample    Blinding   NR  Handling of missing data  Removed from the analysis (15–19%)    Other comments  No investigation of completed suicides  Drop-out rate uncertain |
| ***SAD PERSONS Scale*** | Saunders  2013  UK [18] | Design: Cohort  Setting: Self-harm service at major general hospital ED  Population: Self-harm, N=126  Participation rate: 100% of referrals, approx 60% of self-harmers presenting to ED  Evaluation time: 6 months  Study Period: June 2011-Aug 2011 | Index test  n=126, female: 57.1%  Mean age: 33±14.5  Reference test  Self-harm  6 months; n=31  female: 61%  Mean age: ND  Drop-out rate: 0 | Cut off: ≥7 (high risk)  Sensitivity: 0.06 [0.01, 0.21]  Specificity: 0.97 [0.91, 0.99]  PPV: 40%  NPV: 76% | Other comments  Only self-harm patients referred to specialist self-harm services were included, and these persons might have had higher scores than those not referred. |
| ***Modified SAD PERSONS Scale*** | Bolton et al  2012  Canada  [17] | Design: Prospective cohort  Setting: Psychiatric services at ED, two tertiary care hospitals   Population: Adults, N=4019 presentations  Evaluation time: 6 months  Study period: Jan 1999 to December 2010 | Index test  n=2713  % female and mean age: ND  Reference test  Suicide attempt  n= 87 individuals, 76 completed modified SAD PERSONS Scale  % female and mean age: ND    Drop-out rate: Uncertain | Cut off: high vs moderate and low  Sensitivity: 0.29 [0.19, 0.40]  Specificity: 0.89 [0.88, 0.90]  PPV: 7.4 %  NPV: 97.7% | Sampling method  Consecutive sample    Blinding: ND  Handling of missing data  Removed from the analysis (15–19%)    Other comments  No investigation of completed suicides  Drop-out rate uncertain |
| ***SIS*** | Harriss  2005  UK  [19] | Design: Cohort  Setting: ED, General hospital  Population: N= 4156 patients with deliberate self-harm,  N=6316 episodes Participation rate: ND  Evaluation time: 3-7 years  Study period: Jan 1993-dec 1997 | Index test  n= 2719 patients,  n=3339 episodes  Female: 47.4%  Mean age: ND  Reference test  Suicide, n=54, 44.4 % females  Mean age: ND  Drop-out rate: 230 (11%) | Cut off:10  Sensitivity: 0.76 [0.62, 0.87]  Specificity: 0.49 [0.47, 0.51]  PPV: 4.2%  NPV: 99%  Cut-off:14  Sensitivity: 0.67 [0.53, 0.79]  Specificity 0.75 [0.74, 0.77]  PPV: 4%  NPV: 99% | Handling of missing data  Not reported |
| ***SIS*** | Niméus  2002  Sweden  [20] | Design: Cohort  Setting: Medical ICU  Population: Suicide attempters N=674  Participation rate: 555/674  Evaluation time: Mean 4.5 years (range 10 months to 8.8 years)  Study Period: ND | Index test  n=555, 62.7% females  Mean age: 38.8±16.1 years Violent suicide attempts= 26 (4.7%), repeaters= 246 (44.7%)  Reference test  Suicide, n=22, 50% female  Mean age: 54.8 years  Drop-out rate: 0 | Cut off: 19  Sensitivity: 0.59 [0.36, 0.79]  Specificity: 0.77 [0.74, 0.81]  PPV: 9.7%  NPV: 98% | Other comments  Suicides determined by local dept of forensic medicine and national bureau of statistics, (high data quality) |
| ***SoS-4*** | Bilén  2013  Sweden  [11] | Design  Cohort  Setting  2 large ED  Population  Adults, DSH  N=328  Evaluation time  6 months  Study Period  Mars 2011 - June2011 | Index test  SoS-4  n=325  female: 70%  Mean age: ND  Reference test  Repeated DSH/suicide attempt  n=80  female: ND  Mean age: ND  Drop-out rate  3/328 (1%) | Cut off:0.14  Sensitivity: 0.89 [0.80, 0.95]  Specificity: 0.11 [0.07, 0.16]  PPV: 25%  NPV: 98% | Sampling method  Consecutive sample |
| ***SoS-4*** | Bilén  2013  Sweden  [12] | Design: Cohort  Setting: ED  Population: N= 1524, Adults, DSH  35% history of DSH  57% treated with antidepressants  Participation rate: ND  Evaluation time: 6 months  Study Period: Jan 2003-dec 2005 | Index test  n=1524, female:65%  Mean age: ND  Reference test  Suicide + repeated DSH  n=309, female:70%  Mean age: 39.5 (18–91 years)  Suicide  n=12  Repeated DSH  n=297  Drop-out rate: 0% | Cut off: 0.14  Repeated DSH  Sensitivity: 0.90 (0.86, 0.93)  Specificity: 0.18 (0.16, 0.20)  PPV: 22%  NPV: 88% | Sampling method  Consecutive sample |
| ***SPS*** | Larzelere  1996  USA [21] | Design: Cohort  Setting: Group home  Population: N=840, Adolescents  Evaluation time: mean 15.5 months (follow-up during group home treatment only,  range 4–41.8 months)  Study Period: 1988 - March 1993 | Index test  n=840 patients, female: 34%  Mean age: ND  Reference test  Suicide attempts during group home stay  n=29  Drop-out rate: 6/840 (0.7%) | Cut-off ≥78  Sensitivity: 27.6%  Specificity: 89.7%  PPV: 8.8% | Sampling method  Sample enriched with 11 self-harmers |
| ***SSI-C*** | Beck 1999  USA [2] | Design: Cohort  Setting: Center of cognitive therapy  Population: N=3701, outpatients who were evaluated at intake, 65% history of suicide attempts  Evaluation time: 1979 to 1994  Study Period: 1975 to 1994 | Index test  N=3701, female:56.7%  Mean age: ND  Reference test  Suicide  n:30, female: 40%  Mean age: 41.1±13.68  Drop-out rate: 128 (3%) | Cut-off: derived from ROC analysis  Cut-off for higher risk≥2  Sensitivity: 0.53 [0.34, 0.72]  Specificity: 0.83 [0.82, 0.84]  PPV: 2.4%  NPV: 99.5% | Sampling method  Few inpatients in the sample |
| ***SSI-W*** | Beck  1999  USA  [2] | Design: Cohort  Setting: Center of cognitive therapy  Population: N=3701, outpatients who were evaluated at intake, 65% history of suicide attempts  Evaluation time: 1979 to 1994  Study Period: 1975 to 1994 | Index test  n:3701, female: 56.7%  Mean age: ND  Reference test  Suicide  n:30, female: 40%  Mean age: 41.1±13.68  Drop-out rate: 128 (3%) | Cut off: derived from ROC analysis  Cut off for Higher risk≥16  Sensitivity: 0.80 [0.61, 0.92] Specificity: 0.78 [0.77, 0.79]  PPV: 2.8%  NPV: 99.8% | Sampling method  Few patients in the sample |
| ***SUAS*** | Waern et al  2010  Sweden  [22] | Design: Prospective cohort  Setting: Emergency ward general hospital  Population: Adults (18-68 years) with suicide attempts, N=206 fulfilled study criteria and 165 accepted participation; 162 of these completed the SUAS interview (79% of those who met study criteria)  Evaluation time: 3 years  Study period: ND | Index test  n=162  % female and mean age: ND  Reference test  Repeat suicidal behaviour (fatal or non-fatal), n=61  % female and mean age: ND  Drop-out rate: 0 | Cut off≥24  Sensitivity  BL: 61%  Specificity  BL: 60% | Sampling method  Unclear patient flow  Other comments  “High SUAS” (score above 30) was defined after the fact, after perusal of data. It is preferable to show sensitivity and specificity for the optimal cut-off (24) as determined by the ROC analysis. |

Abbreviations

ND: Not declared; ED: Emergency Department; DSH: Deliberate Self-Harm; BL: Baseline

References

1. Beck AT, Brown G, Berchick RJ, Stewart BL, Steer RA. Relationship between hopelessness and ultimate suicide: a replication with psychiatric outpatients. Am J Psychiatry 1990;147:190-5.

2. Beck AT, Brown GK, Steer RA, Dahlsgaard KK, Grisham JR. Suicide ideation at its worst point: a predictor of eventual suicide in psychiatric outpatients. Suicide Life Threat Behav 1999;29:1-9.

3. Keller F, Wolfersdorf M. Hopelessness and the tendency to commit suicide in the course of depressive disorders. Crisis 1993;14:173-7.

4. David, Klonsky, Kotov R, Bakst S, Rabinowitz J, Bromet EJ. Hopelessness as a predictor of attempted suicide among first admission patients with psychosis: a 10-year cohort study. Suicide Life Threat Behav 2012;42:1-10.

5. Nimeus A, Traskman-Bendz L, Alsen M. Hopelessness and suicidal behavior. J Affect Disord 1997;42:137-44.

6. Sidley GL, Calam R, Wells A, Hughes T, Whitaker K. The prediction of parasuicide repetition in a high-risk group. Br J Clin Psychol 1999;38 ( Pt 4):375-86.

7. Posner K, Brown GK, Stanley B, Brent DA, Yershova KV, Oquendo MA, et al. The Columbia-Suicide Severity Rating Scale: initial validity and internal consistency findings from three multisite studies with adolescents and adults. Am J Psychiatry 2011;168:1266-77.

8. Carter GL, Clover KA, Bryant JL, Whyte IM. Can the Edinburgh Risk of Repetition Scale predict repetition of deliberate self-poisoning in an Australian clinical setting? Suicide Life Threat Behav 2002;32:230-9.

9. Randall JR, Rowe BH, Dong KA, Nock MK, Colman I. Assessment of self-harm risk using implicit thoughts. Psychological Assessment 2013;25:714-721.

10. Roaldset JO, Linaker OM, Bjorkly S. Predictive validity of the MINI suicidal scale for self-harm in acute psychiatry: a prospective study of the first year after discharge. Arch Suicide Res 2012;16:287-302.

11. Bilen K, Ponzer S, Ottosson C, Castren M, Owe-Larsson B, Ekdahl K, et al. Can repetition of deliberate self-harm be predicted? A prospective multicenter study validating clinical decision rules. J Affect Disord 2013;149:253-8.

12. Bilen K, Ponzer S, Ottosson C, Castren M, Pettersson H. Deliberate self-harm patients in the emergency department: who will repeat and who will not? Validation and development of clinical decision rules. Emerg Med J 2013;30:650-6.

13. Cooper J, Kapur N, Dunning J, Guthrie E, Appleby L, Mackway-Jones K. A clinical tool for assessing risk after self-harm. Ann Emerg Med 2006;48:459-66.

14. Randall JR, Rowe BH, Colman I. Emergency department assessment of self-harm risk using psychometric questionnaires. Can J Psychiatry 2012;57:21-8.

15. Steeg S, Kapur N, Webb R, Applegate E, Stewart SLK, Hawton K, et al. The development of a population-level clinical screening tool for self-harm repetition and suicide: The ReACT Self-Harm Rule. Psychological Medicine 2012;42:2383-2394.

16. Simon GE, Rutter CM, Peterson D, Oliver M, Whiteside U, Operskalski B, et al. Does Response on the PHQ-9 Depression Questionnaire Predict Subsequent Suicide Attempt or Suicide Death? Psychiatr Serv 2013.

17. Bolton JM, Spiwak R, Sareen J. Predicting suicide attempts with the SAD PERSONS scale: a longitudinal analysis. J Clin Psychiatry 2012;73:e735-41.

18. Saunders K, Brand F, Lascelles K, Hawton K. The sad truth about the SADPERSONS Scale: an evaluation of its clinical utility in self-harm patients. Emerg Med J 2013.

19. Harriss L, Hawton K. Suicidal intent in deliberate self-harm and the risk of suicide: the predictive power of the Suicide Intent Scale. J Affect Disord 2005;86:225-33.

20. Niméus A, Alsén M, Träskman-Bendz L. High suicidal intent scores indicate future suicide. Archives of Suicide Research 2002;6:211-219.

21. Larzelere RE, Smith GL, Batenhorst LM, Kelly DB. Predictive validity of the suicide probability scale among adolescents in group home treatment. J Am Acad Child Adolesc Psychiatry 1996;35:166-72; discussion 172.

22. Waern M, Sjostrom N, Marlow T, Hetta J. Does the Suicide Assessment Scale predict risk of repetition? A prospective study of suicide attempters at a hospital emergency department. Eur Psychiatry 2010;25:421-6.
